# On-Orbit Performance and Calibration of the Soft X-Ray Telescope on *Yohkoh*

**DOI:** 10.1007/s11207-015-0842-5

**Published:** 2016-02-17

**Authors:** Loren W. Acton

**Affiliations:** Department of Physics, Montana State University, P.O. Box 173840, Bozeman, MT 59717-3840 USA

**Keywords:** Corona, Data calibration, Data management, Flares, Instrumental effects, X-Ray bursts

## Abstract

This paper documents details of the on-orbit performance, data problem solving, and calibration of the Soft X-ray Telescope (SXT) experiment on *Yohkoh*. This information is important to a full understanding of the strengths and weaknesses of the SXT data set. The paper begins with summaries of SXT calibration issues and how they have been addressed, operational anomalies experienced during the mission, and a brief discussion of the SXT optical train. The following section on the accuracy of *Yohkoh* pointing determination provides information important for alignment of SXT images with each other and with other solar data. The remainder of the paper gives details of work by the experiment team to understand and ameliorate the many instrument anomalies and changes which impacted the scientific data.

## Introduction

Scientific operation of the Japan–US–UK solar activity mission *Yohkoh* extended from September 1991 to 14 December 2001. The *Yohkoh* mission (Ogawara *et al.*, 1991) and instrumentation are described in the collection of articles edited by Švestka and Uchida (1991). The individual papers are also available on the *Yohkoh* Legacy Archive (YLA) website (Takeda *et al.*, 2009; Takeda, 2015). The solar observations by *Yohkoh* are of continuing scientific importance and have been archived in their entirety in the YLA (http://solar.physics.montana.edu/ylegacy/). The user-friendly YLA includes extensive documentation on mission operations and data search, browse and reference resources as well as fully reduced and calibrated data, flare lists, movie makers, *etc*. The *Yohkoh* data archives at ISAS/JAXA in Japan (http://darts.isas.jaxa.jp/solar/yohkoh/) and at the Solar Data Analysis Center of NASA Goddard Space Flight Center (http://umbra.nascom.nasa.gov/yohkoh/) carry all mission data but without all of the ancillary resources available at the YLA.

The Soft X-ray Telescope (SXT) was a primary *Yohkoh* instrument (Tsuneta *et al.*, 1991). SXT operating modes were very flexible but the telescope normally acquired full Sun images of the quiet corona (full frame images or FFI) at five arcsec pixel and approximately 5 min temporal resolution through one of five thin-film X-ray analysis filters. In parallel, partial Sun images (partial frame images or PFI) were acquired of, usually, the brightest active region at full angular resolution ($2.455~\mbox{arcsec}\,\mbox{pixel}$) and about 30 s temporal resolution with automatic exposure control. In flare mode FFIs were not acquired so that the full SXT telemetry allocation could be devoted to PFI observations with a time resolution as short as 2.4 s, depending on the designated size of the observing region, also with automatic exposure control.

*Yohkoh* normally pointed at solar disk center with solar north up and solar east to the left on SXT images. This enabled imaging the solar corona out to $1.3~R_{\odot}$ on the $1024\times1024~\mbox{pixel}$ CCD for each FFI.

The SXT acquired over $8.2\times10^{5}$ FFIs and $5.9\times10^{6}$ PFIs between 3 September 1991 and 14 December 2001. The full-Sun data have had short and long exposures combined (level-2 products) into $3.0\times10^{5}$ composite images to increase the intensity range of each image. Each composite image has a corresponding statistical uncertainty image. The most cosmetically perfect 296 574 of the thin-filter level-2 composites have been processed to level-3 for cinematographic viewing and morphological analysis. The SXT observations were unprecedented in resolution and cadence and remain a valuable and unique record of high-energy solar activity from the peak of sunspot cycle 22 to the peak of cycle 23.

A compilation of *Yohkoh* publications may be found at http://www.lmsal.com/~aschwand/publications/yohkoh.html. Excellent examples of use and interpretation of SXT data are in papers by, *e.g.*, Shibata *et al.* (1992), Masuda *et al.* (1994), Tsuneta (1996), Sterling and Hudson (1997), and McKenzie (2000).

The layout of this paper provides, first, short summaries of important topics later followed by the analysis that support and illustrate the conclusions. Thus, tables and figures may be referenced well in advance of their appearance in the text.

## SXT Calibration

All elements of the SXT optical train (Section 4) were calibrated in the laboratory. End-to-end testing of the SXT under vacuum verified focus and, although not rigorously in flight configuration, were consistent with piecewise calibration results. *SXT Calibration Notes 5* (Lemen and Hudson, 1990), *29* (Acton, 1992), and *30* (Lemen, 1992), which are available in the YLA, detail aspects of pre-launch calibration and testing.

Following the launch of *Yohkoh* on 30 August 1991 regular sequences were run to enable tracking of changes in SXT calibration. The presentation of this post-launch calibration, using data acquired on orbit, is the purpose of this paper. The following items important to the analysis of SXT data are introduced in this section:

- X-ray scatter;
- X-ray vignetting;
- Entrance filter failures;
- Analysis filter failures;
- Contamination of the X-ray mirror;
- Contamination of the CCD;
- Gain of CCD camera amplifier;
- Ionizing radiation damage to the CCD;
- Damage to the CCD by high energy particles;
- Errors and uncertainties.

### X-ray Scatter

Figure imperfections and dust cause X-rays to scatter off of grazing incidence X-ray mirrors (Zhao and Van Speybroeck, 2003; Spiga, 2007). Most scattering goes into the axial plane, *i.e.*, perpendicular to the mirror surface (Aschenbach, 1985).

**Figure 1:**
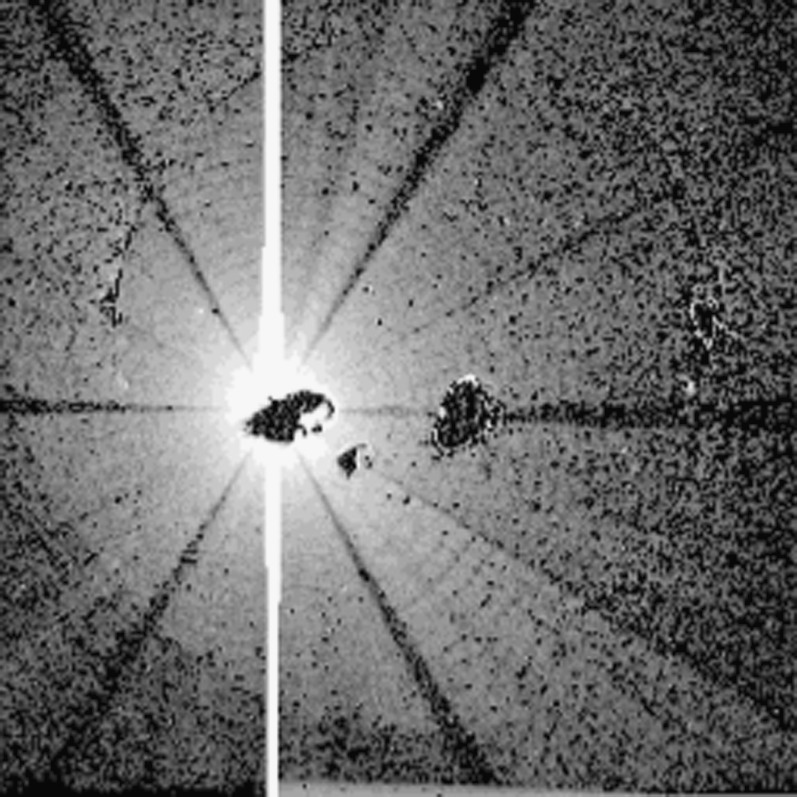
AlMg ‘starburst’ image (scaled to the power 0.3 to reveal faint parts) showing scattered X-rays from the C6.7 flare at 14 October 1995, 06:58:02 UT, with the pre-flare image of 06:53:22 subtracted. The radial dark spokes are shadows of the entrance filter frames. We believe the faint arc in the lower left is a vignetting effect. The thin quasi-circumferential arcs are shadows of the stainless steel filter support mesh.

Although the point spread function of the SXT was well characterized the laboratory data were inadequate to define the scattering wings (Martens, Acton, and Lemen, 1995). Because the *Yohkoh* flare flag is inoperative during predicted passages through the South Atlantic Anomaly (SAA) FFI images continue to be acquired. Flares occurring during such periods may have strong overexposure at the flare site but with scattering wings (‘starburst’ images) well recorded, such as is illustrated in Figure 1. Several studies of such starburst images are presented in http://solar.physics.montana.edu/ylegacy/observ_notes.html. The scatter correction algorithms have been used in studies of coronal holes and other faint coronal structures (*e.g.*, Hara *et al.*, 1994; Hara, 1997; Foley, Culhane, and Acton, 1997). The SolarSoft program *sxt_decon.pro* corrects by deconvolution, as best we know how, for scattering. Unfortunately, although the scattering goes approximately as $r^{-2}$ it is observed to vary with epoch (changes with failure of entrance filter sections), azimuth (entrance filter changes and shadowing from entrance filter frames), and photon energy (higher energy photons scatter more) (Acton, 1995).

McKenzie *et al.* (2002) evaluated the scattering fraction by analysis of 35 over-the-limb flares throughout the mission, and analysis of a spikey arcade from the 18 December 1998 event. His result for the scattering fraction of each SXT analysis filter is as follows.
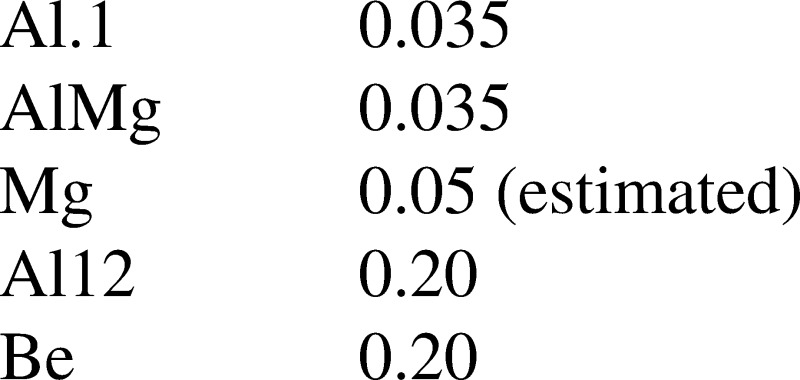
 These values are set as the default scattering fractions in *sxt_ psf.pro* which is called by *sxt_decon.pro*, the program used to correct, as best we know how, for scattering wings. The default scattering slope decreases as $r^{-2}$.

All YLA level-2 and level-3 FFI data products have employed *sxt_ decon.pro* for scatter correction. PFI images can seldom be corrected for scattering because full disk intensities are not available. As the PFI images are normally of the brightest X-ray features scattering decreases signal in the brightest parts of the image.

It must be noted that scatter corrections are imperfect at best. For any given situation errors in the scattering fraction and the slope of the scattering wings are difficult to quantify. Quantitative analysis of coronal holes or other faint features when bright active regions are present requires extra care in estimating uncertainties.

### X-ray Vignetting

The grazing-incidence design of grazing-incidence X-ray telescopes makes them more subject to off-axis vignetting than traditional optical telescopes. Fuller, Lemen, and Acton (1994) present the SXT off-axis calibration measurements and derive the vignetting function used in the correction program *sxt_off_axis.pro*. The chosen vignette function is based on both laboratory and flight observations and is modeled as two non-concentric cones. This vignette correction has been applied to all YLA level-2 and level-3 data products.

Shin and Sakurai (2015a,b) numerically simulated SXT vignetting and concluded that SXT vignetting is better described by a model taking into account the fact that the optical and geometric axes of the telescope are offset. The differences between the two corrections do not appear to be large but are probably real. Unfortunately, an improved correction algorithm has not been published so the YLA products currently employ the function in Fuller, Lemen, and Acton (1994).

### Entrance Filter Failures

By far the most troublesome calibration problem for the SXT has been estimation of the change in spectral sensitivity related to entrance filter failures and correction for the stray visible light which thereafter entered the telescope. During the mission the routine stray-light monitor signals tracked stray-light levels within the instrument and provided immediate notice of new failures. Having no means to determine the fraction of filter(s) which had failed we initially assumed that an entire $30^{\circ}$ sector had failed at each stray-light step. With the detailed analysis presented in Section 7, and the availability of the entire SXT data set to study, it has proven possible to improve the estimate of the increase in spectral sensitivity for each failure epoch. Conclusions of which sectors failed at each stray-light step are presented in Table 8. Analysis of the entrance filter open area, used for SXT sensitivity computation, is summarized in Section 7.8.

Techniques for removing stray visible light from the X-ray images are discussed in Section 8.

### Analysis Filters

The only SXT analysis filter known to have changed properties was the Al.1 filter. The changes involved the opening of three or more pinholes. Fortunately, none of these pinholes were in the filter area where the solar X-ray image fell for normal pointing so they had an insignificant impact on X-ray sensitivity. However, the stray visible light diffracting through the pinholes and that passing directly through the filter material (at approximately the $10^{-6}$ level) contaminated the X-ray image at a level comparable to the quiet X-ray coronal signal. This contamination is partially corrected through the use of terminator images (see Section 8) but remains a problem for quiet Sun analysis. The signal levels of active regions and flares are sufficiently great that stray-light contamination is not usually of major importance. For browsing, the AlMg images, which are the least affected by stray light, are nearly the same in appearance as the Al.1 images.

### X-Ray Mirror

**Figure 2:**
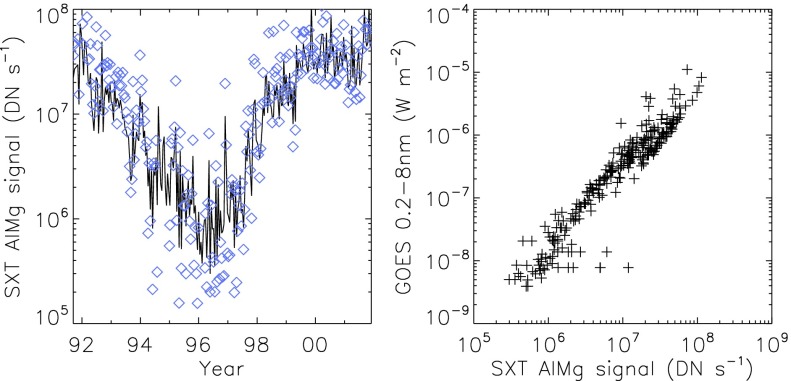
Comparison of concurrent bi-weekly samples of 0.2 – 0.8 nm GOES flux and the total signal in SXT AlMg composite images. Blue diamonds in the left panel are GOES signals multiplied by $4\times 10^{13}$.

The on-orbit contamination of SXT aspect sensor optics (Section 6) and the CCD (Section 9.4) suggests the possibility of contamination of the X-ray mirror. Such contamination would have impacted X-ray reflectivity and scatter. We have not seen evidence of changes in X-ray scatter in the data, although subtle changes could easily have escaped notice. The mission-long comparison of SXT and GOES signals in Figure 2, while only approximate because of the significantly different band passes of the two instruments, shows no indication of a drastic change in SXT X-ray sensitivity.

### CCD Contamination

Contamination of the CCD to a level that could be detected in thin-filter X-ray images was observed after a few months of operation in the form of small areas of decreased signal aligned with the CCD gate structure (along the CCD rows). The contamination artifacts disappeared when the CCD temperature was raised to about 0 °C. In 1992 the CCD was permitted to warm up to 0 °C three times and baked out to +24 °C once. Beginning in January 1993 regular bakeouts of the CCD were implemented. The dates of all CCD warmups and bakeouts are provided in the YLA. The need for and frequency of bakeouts decreased with time as *Yohkoh* outgassed. We have no evidence of residual contamination on the CCD following bakeouts.

### On-Chip CCD Amplifier Gain

The pre-launch CCD on-chip gain was set to approximately 100 electrons $\mathrm{DN}^{-1}$ to best match the saturation levels of the analog-to-digital-converter and the CCD full-well capacity. 100 electrons $\mathrm{DN}^{-1}$ was used as default thereafter. In-flight measurements of camera gain were challenging for a number of reasons but indicated a gain of about 90 electrons $\mathrm{DN}^{-1}$ (LaBonte, 1996). SXT analysis software was updated in 2014 to reflect this gain. Derivation of emission measures from SXT data accomplished prior to 2014 may therefore be low by 10 % because of this error. See Section 9.1 for further discussion of CCD on-chip amplifier gain.

### Ionization Damage to the CCD

Although damage to the CCD optical response from ionizing X-ray radiation was quickly evident in aspect sensor images we have discovered no evidence that it reached a level that affected X-ray sensitivity (see Section 9.2.3). It was observed that severe X-ray overexposure caused an increase in dark current (Section 9.2.2) which disappeared over periods of a few days. If appropriate dark frames were not acquired in and around these transient events the corrected image in overexposed areas could be either too high or too low. In general, level-2 data in the YLA have been screened to eliminate cases of severe ill correction.

### Energetic Particle Damage to the CCD

Ionization damage and Si lattice dislocations within CCD pixels from high energy space radiation cause increased dark current (so-called dark spikes or hot pixels) and loss of charge transfer efficiency (CTE) (Janesick, 2001). This happens on a pixel by pixel basis and, in the case of SXT, was not permanent with time. CCD bakeouts partially corrected some radiation damage effects as did, I believe, the every-orbit UV flood. Most dark spikes are removed from SXT images by dark-frame subtraction. The decrease in CTE of single damaged pixels is not observable in the SXT images. Properties and numbers of dark spikes are discussed in more detail in Section 9.3.

### Errors and Uncertainties

Pre-launch calibration of the effective area of the SXT (Lemen, 1993) aimed to attain an absolute accuracy of a few percent or better. SXT images are compressed from 12 to 8 bits per pixel for downlink (Tsuneta *et al.*, 1991). The uncertainty introduced by decompression of the lossy compression algorithm is always less than counting statistics and is returned by *sxt_prep.pro*. An estimate of the uncertainty introduced by counting statistics is returned by the program *sxt_dn_uncert.pro*. The uncertainty in CCD amplifier gain (Section 9.1) enters directly in the conversion of instrument units into emission measure and could be as large as 10 %. Deconvolution of scattered X-rays (Section 2.1) by *sxt_decon.pro* and correction for telescope vignetting (Section 2.2) by *sxt_off_axis.pro* are certainly not perfect but are difficult to quantify. The errors associated with stray-light correction and dark signal subtraction (Acton, 1997) have not been quantified. These unquantified errors may be substantial for faint coronal sources, *e.g.*, coronal holes, but it is certain that the corrected images are closer to the truth than if the corrections are not applied. Systematic errors from, *e.g.*, mirror or filter contamination are possible but unknown. All of the SXT data products in the YLA have been adjusted for all known and quantified instrumental effects, as appropriate.

## SXT Operational Anomalies

The SXT achieved design performance, returning excellent soft X-ray images for the duration of the mission. Unfortunately, the instrument experienced technical anomalies that complicated data reduction and analysis. It is the purpose of this document to describe and explain these issues and to detail the corrective measures that have enabled fruitful solar studies during and since the *Yohkoh* mission.

A list of SXT on-orbit anomalies is presented in Table 1.

**Table 1:** SXT operational issues.

(a)	Attitude errors requiring, when possible, manual corrections to the *Yohkoh* attitude (ATT) data base (Section 5)
(b)	A steep decrease in the signal level through the optical aspect sensor beginning immediately after launch (Section 6)
(c)	A series of failures of entrance filters during the mission which introduced visible stray light into the telescope, compromising X-ray images and altering the spectral response of the SXT (Sections 7 and 8)
(d)	Pinholes and optical transmission through the Al.1 X-ray analysis filter affected the X-ray image (Section 8)
(e)	Time-dependent X-ray radiation damage to the CCD which impacted sensitivity to visible light and, to much a lesser degree, X-ray sensitivity (Section 9)
(f)	Energetic charged particle damage to the CCD causing dark spikes and changes in dark signal (Section 9)
(g)	Condensation of some contaminant on the CCD requiring periodic bakeouts to remove it (Section 9.4)
(h)	Dark signal subtraction from the X-ray images (Section 10)
(i)	Telemetry drop outs causing gaps in SXT images

The SXT_Observation_Notes section of the YLA provides extensive analysis and illustration of these and other data anomalies (http://solar.physics.montana.edu/ylegacy/observ_notes.html). This paper will document the steps that have been taken to create the best and most accurate data products from SXT. Subsequent sections include illustrations and descriptions of the anomalies, how each affected the SXT images, and corrections applied when creating the higher-level data products.

## The SXT Optical Train

The SXT on *Yohkoh* comprises a two-element grazing incidence mirror feeding a CCD detector in the focal plane (Tsuneta *et al.*, 1991). The entrance aperture, defined by the projected area of the first element of the mirror, is an annulus 0.362 mm wide and 230.65 mm in diameter (Acton, 1999). A confocal optical telescope (called the aspect sensor) is nested within the X-ray mirror. Dual filter wheels with both X-ray and optical filters and a rotating shutter complete the instrument. Extensive technical details of the SXT, including many engineering drawings, are presented in the Soft X-ray Telescope (SXT) for Solar-A Experiment Interface Control Agreement (EICA,^1^ 1990).

**Figure 3:**
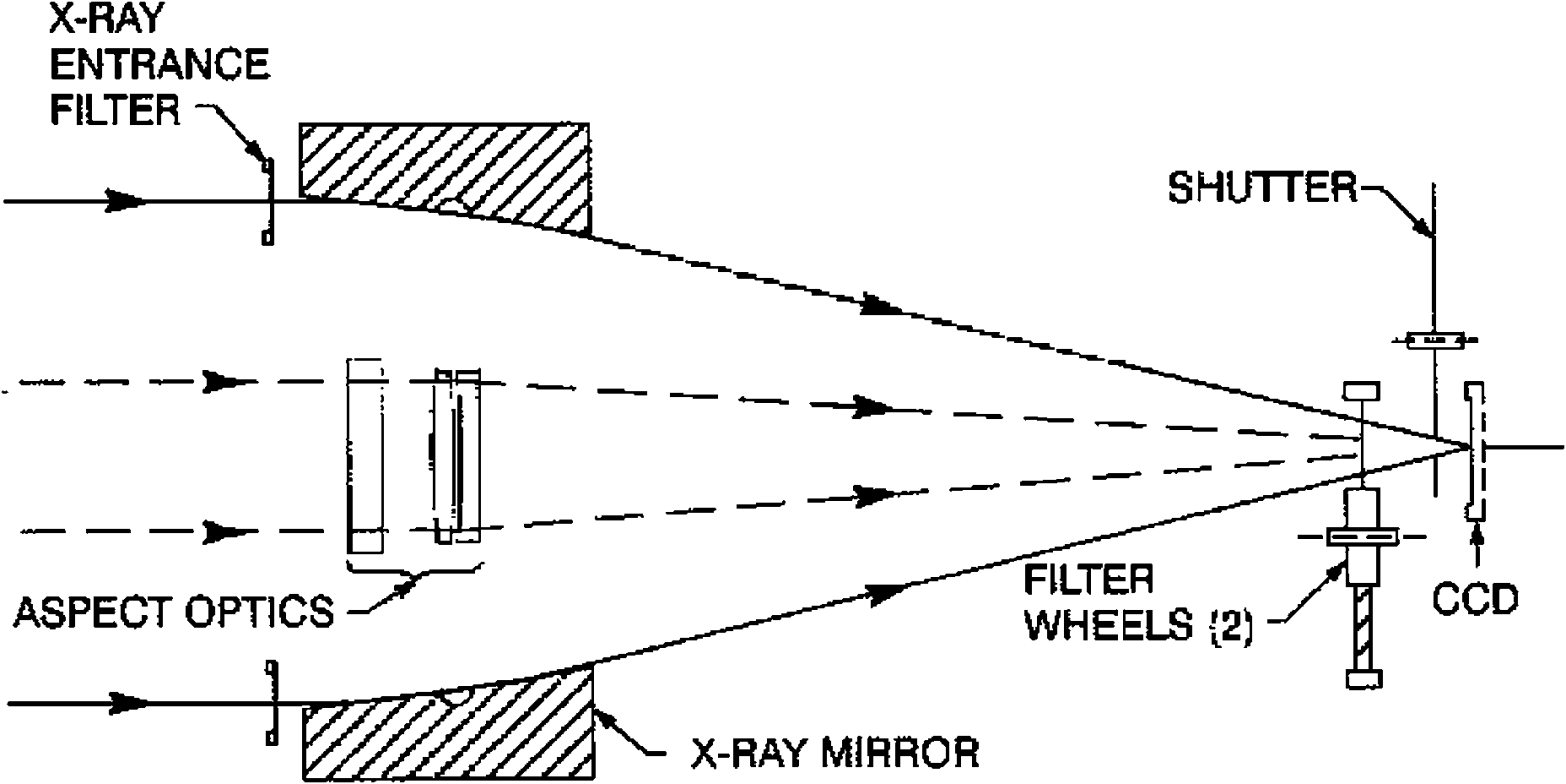
Schematic diagram of key elements of the SXT optical train.

**Figure 4:**
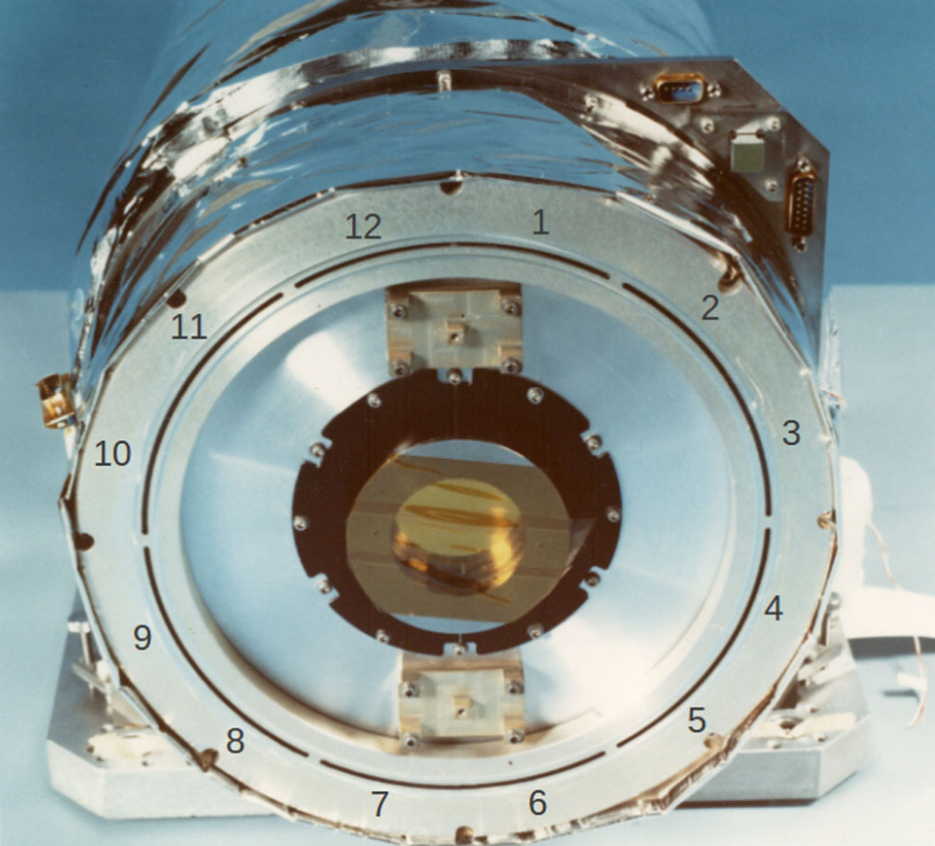
SXT entrance annulus with filter sectors numbered.

Figure 3 depicts the major components of the SXT while Figure 4 illustrates the front end of the SXT with the individual $30^{\circ}$ sectors numbered clockwise from 1 to 12. In the sections that follow we will refer to this figure in detailing the sequence of entrance filter failures throughout the operational life of SXT.

Dual entrance filters were employed on the SXT to guard against pinholes in the opaque metallic coating permitting the full solar spectrum to enter the telescope. The coatings were made as thin as possible in order to provide the greatest feasible X-ray transmission. Thus, even apart from pinholes, a single filter is not completely opaque, transmitting of the order of $10^{-7}$ to $10^{-6}$ in the visible.

**Figure 5:**
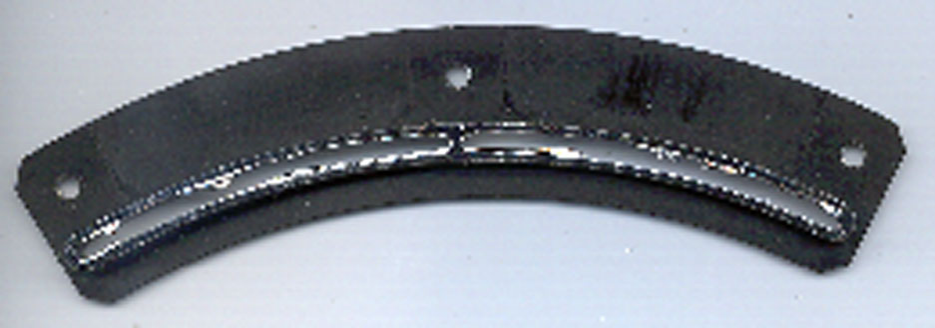
SXT entrance filter frame. The filter membrane itself has been destroyed in testing, showing the catastrophic degradation typical of the failure of thin films of this type.

**Figure 6:**
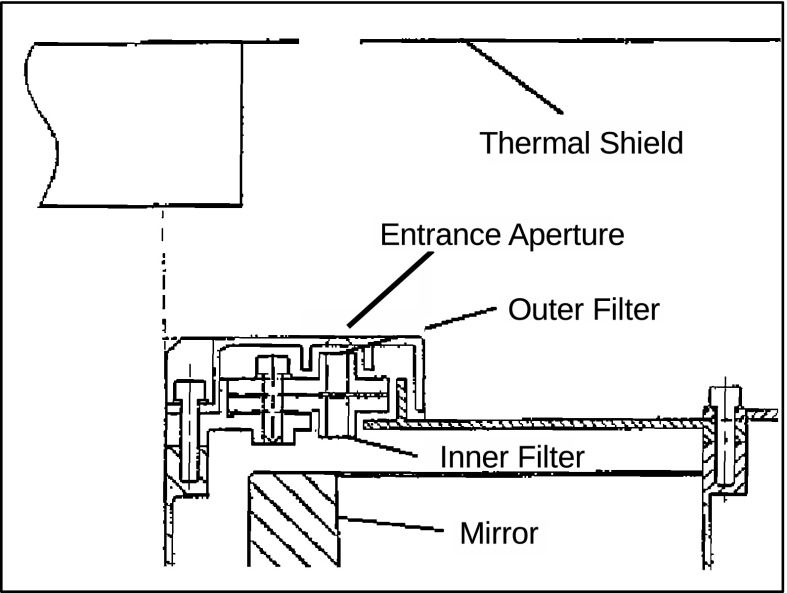
Positioning of SXT entrance filters.

The individual filters are mounted on frames each of which covers a $60^{\circ}$ sector of the entrance annulus. Each frame comprises two individual $30^{\circ}$ sections as shown in Figure 5. The filter membrane is lexan 180 nm thick covered with 70 nm of Ti and 90 nm of Al with the Al being the outer coating. As may be seen in Figure 6 the outer filter is positioned so that the Al coating faces the Sun while the inner filter has the lexan in the solar direction. This mounting scheme permitted purchasing only a single type of filter assembly but it exposed the lexan of the inner filter to the Sun and space following the failure of an outer filter.

## *Yohkoh* Pointing

The *Yohkoh* attitude control system (ACS) was designed to point at Sun-center with stability of order one $\mbox{arcsec}\,\mbox{s}^{-1}$ and several $\mbox{arcsec}\,\mbox{min}^{-1}$ (Ogawara *et al.*, 1991). The spacecraft (S/C) included two Sun sensors, a star tracker, geomagnetic sensors, and four gyroscopes for attitude determination. For on-orbit alignment determination the SXT included a small white-light telescope referred to as the aspect sensor (Tsuneta *et al.*, 1991). The HXT aspect system (HXA) employed linear CCDs, operating in the visible as limb sensors, to provide pointing information in $X$ and $Y$ to one or two arcsec accuracy (Kosugi *et al.*, 1991). The *Yohkoh* attitude (ATT) data are derived from HXA signals. Between HXA measurements the $X$ and $Y$ ATT data are computed by interpolation of S/C gyro data which is transmitted at a higher rate.

The *Yohkoh* ACS normally maintained solar north upwards on the SXT CCD independent of p-angle. That is, the solar rotation axis should always parallel the CCD pixel columns. Roll was determined from S/C gyro data with reference to the Canopus star tracker. When Canopus was occulted roll was determined from the gyros alone. Inflight calibration based on SXT visible-light aspect images and analysis of Mercury transit observations (Wuelser *et al.*, 1998) revealed an offset in roll. That is, SXT CCD columns are rotated $0.7^{\circ}$ clockwise from S/C coordinates on the SXT images. The roll angle given in ATT records is the S/C roll angle. The Yohkoh/SXT/SolarSoft program *get_roll.pro*, and all other SXT data processing programs, correct for the $0.7^{\circ}$ offset so that the SXT processed images in the YLA all have solar north straight up.

Preparation of the *Yohkoh* ATT data base is carried out as part of creation of level-0 data products from the downlinked telemetry. One ATT record is created for each and every SXT image. The records contain the coordinates of Sun-center in full-resolution pixel (FR) units ($2.455~\mbox{arcsec}\,\mbox{pixel}^{-1}$) of the SXT CCD detector. The ATT file format is detailed in the Appendix.

The ATT values incorporate a number of adjustments based upon inflight calibration (Wuelser *et al.*, 1998). ATT information is incorporated in the header of SXT FITS-format images available through the YLA. For *Yohkoh* mission-specific (XDA) format data the ATT files are separate files.

### *Yohkoh* Attitude Errors

There are several sources of error in the ATT data:

1. Imperfect illumination of the HXA for certain pointings.
2. Aging of the HXA.
3. Failure of one of the S/C gyros.
4. Increase in gyro drift rate with age.
5. Loss of fine pointing, *i.e.*, pointing not under control of the fine Sun sensors.
6. HXA samples were not taken frequently enough at medium telemetry rate to provide anchors for gyro signal interpolation at the end of orbits near sunset. This problem became evident late in the mission when gyro drift was high.
7. Loss of the SXT aspect telescope on 13 November 1992 due to failure of an entrance filter. After this time it was no longer possible to acquire aspect sensor images.
8. Corrupted or missing telemetry downlink data.

Pointing anomalies tended to occur at times of loss of fine pointing in connection with non-standard operation such as partial solar eclipses. As *Yohkoh* aged the frequency of poor ATT data increased.

**Figure 7:**
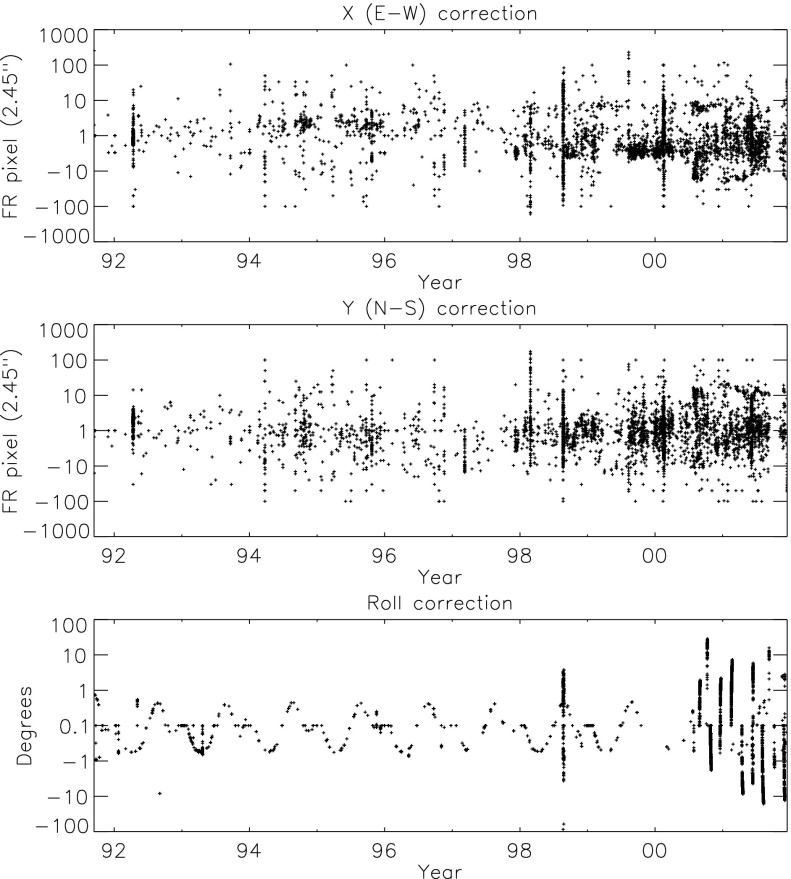
Corrections applied to the *Yohkoh* attitude data base. In order to display the broad range of ATT corrections they have been plotted logarithmically. $X\mbox{--}Y$ corrections less than one pixel and roll corrections less than $0.1^{\circ}$ are not included on the plots. The vertical axis in the upper two plots indicate the magnitude, in full-resolution (FR) pixels, that have been applied to the ATT values. The minus values are a subtractive correction. The bottom plot similarly shows the roll corrections with positive representing a clockwise rotation of the SXT image.

From time to time the ATT data base has had corrections applied as pointing maladies were identified and improved calibration became available. As of the time of this writing the ATT data base is in version 23 (ATT_23). The version in use at the termination of *Yohkoh* scientific operation on 14 December 2001 was ATT_08. FFIs are more amenable to ATT correction than PFIs because the entire solar limb is available for adjustment of the ATT records by limb fitting with the program *fit_limb.pro*. The limb is clearly evident, in quiet coronal regions, by the factor of two brightening, because of the doubling of coronal path length, as the limb is passed. In the best cases, with most of the limb unobscured by active regions, fitting accuracies of a fraction of a pixel are achieved. The corrected values are not, in general, as accurate as the good ATT values derived from telemetry but are considerably better than the original, uncorrected, values. Figure 7 illustrates ATT errors identified and corrected in the ATT_23 data base.

#### ATT Roll Errors

Because S/C roll changed smoothly and fairly slowly roll corrections for a given interval have been applied by interpolation or extrapolation to every affected roll record. Thus, roll corrections have been applied when appropriate to both SXT PFI and FFI records. In contrast, yaw ($X$ or E–W direction) and pitch ($Y$ or N–S direction) errors change quickly so corrections are only possible for FFIs where limb fitting is possible.

The large roll corrections in 1998 reflected *Yohkoh* entering safe-hold mode following the partial eclipse of 22 August 1998. Until late 2000 most roll corrections resulted from corrupted or missing telemetry. In these cases the roll value was set to the first valid roll datum following the corrupted records. The accuracy of these extrapolations are generally better than $0.1^{\circ}$. Roughly 2.1 % of SXT ATT records have been adjusted for roll.

#### ATT X–Y Errors

The SXT requires precision attitude information for every image in order to co-align X-ray images for time-lapse review and for registration of images with other sources of solar imagery, *e.g.*, magnetograms, $\mathrm{H}\alpha$ pictures, *etc*.

It has proven possible to quantify ATT-$XY$ errors for SXT FFIs because the solar limb is always recorded. Our procedure for searching for pointing problems is as follows. First, composite (level-2) images are prepared by combining short and long exposures to eliminate areas of detector saturation as much as possible. These composite images are formed from either two or three exposures depending on the duration of the longest exposure.

The next step is to clean artifacts from the composite images, logarithmically compress the signals, and rebin the quarter resolution ($9.82~\mbox{arcsec}\,\mbox{pixel}^{-1}$) images to half resolution ($4.91~\mbox{arcsec}\,\mbox{pixel}^{-1}$). These processed images are co-aligned based on the best ATT data available, collected into image cubes, and run as movies at various frame rates. In movie mode shifts of 5 – 10 arcsec are readily discernible. If active regions are seen to shift position on the disk from frame to frame, but the limb does not move simultaneously, this indicates that the ATT data for the short exposure(s) of the composite image are in error.

Images with incorrect ATT values are flagged for further processing. The necessary $X\mbox{--}Y$ corrections to ATT are derived by fitting a circle (of the proper diameter for the epoch) to the full-disk X-ray image and comparing the circle center to the Sun center position recorded in the ATT record. The ATT corrections thus derived have been incorporated into the ATT data base. Figure 7 illustrates the ATT adjustments. About 1.5 % of FFIs (4562 in $X$ and 4568 in $Y$ of 298 288 images) have had their ATT data thus adjusted. This has been an iterative process as small errors show up better after large image shifts are corrected. Small (${<}\,10~\mbox{arcsec}$) ATT errors undoubtedly still remain. However, the mission-long movie available through the YLA now runs quite smoothly, even through times of solar eclipse.

### ATT Reliability

The accuracy of *Yohkoh* ATT data has been checked by limb-fitting SXT aspect sensor images acquired prior to November 1992. Due to the 4.91 arcsec pixelization of the images it is not feasible to obtain a fitting accuracy better than about 2.5 arcsec although it appears that the best ATTs are good to 1 arcsec.

During the 3746 days of *Yohkoh* scientific operation there were 3699 days producing data with ATT records. Of these 3699 days there were 1120 days, slightly less than one-third, which required ATT-$XY$ correction. Recall that ATT correction in $X$ and $Y$ is only feasible for FFIs and has only been done for those FFIs of adequate quality to be incorporated into level-2 composite images. In most cases these ATT corrections were needed for one or a few FFIs on any given day. For such days nearly all of other ATT values (*i.e.*, ATT for the PFIs also) will be correct and the images can be co-aligned, *etc*., with confidence.

**Figure 8:**
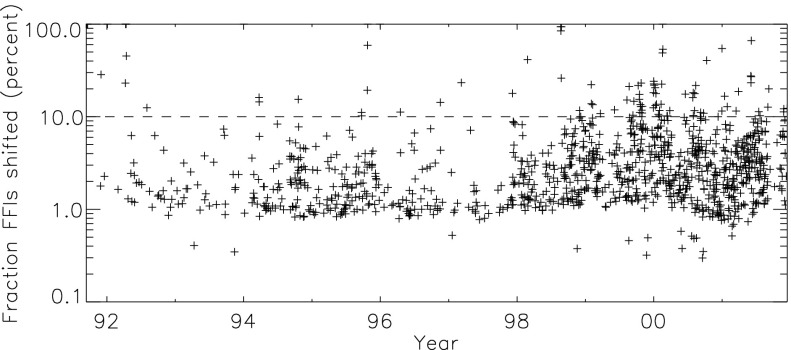
The percentage of FFIs requiring ATT adjustment in either $X$ or $Y$ for each day of the *Yohkoh* mission for which ATT adjustment was applied.

For days with more than, say, 10 % of the FFIs requiring ATT correction in $X$ and/or $Y$ extra caution should be exercised in the use of SXT pointing (ATT) data. Figure 8 illustrates, for every day of the *Yohkoh* mission for which an ATT adjustment was required (1120 days), the fraction of composite FFIs requiring ATT-$XY$ adjustment. There are 101 of 3699 days for which more than 10 % of composite FFIs required ATT-$XY$ shifts. These dates are listed in Table 2 and justify extra care for co-registration of PFIs.

**Table 2:** Dates (dd-mmm-yy) with questionable ATT.

13-Sep-91	14-Sep-91	15-Sep-91	18-Sep-91
19-Sep-91	20-Sep-91	01-Dec-91	04-Jan-92
09-Apr-92	10-Apr-92	11-Apr-92	12-Apr-92
13-Apr-92	14-Apr-92	02-Aug-92	01-Jan-94
24-Mar-94	25-Mar-94	20-Oct-94	23-Sep-95
24-Sep-95	24-Oct-95	25-Oct-95	26-Oct-95
31-Dec-95	17-Apr-96	17-Nov-96	09-Mar-97
07-Dec-97	26-Mar-98	22-Aug-98	23-Aug-98
25-Aug-98	26-Aug-98	01-Dec-98	01-Feb-99
02-Feb-99	09-Feb-99	22-Mar-99	06-Jun-99
11-Aug-99	23-Aug-99	12-Sep-99	17-Sep-99
10-Oct-99	16-Oct-99	17-Oct-99	19-Oct-99
22-Oct-99	24-Oct-99	26-Oct-99	29-Oct-99
31-Oct-99	01-Nov-99	30-Dec-99	31-Dec-99
01-Jan-00	02-Jan-00	03-Jan-00	09-Jan-00
10-Jan-00	13-Jan-00	16-Jan-00	26-Jan-00
31-Jan-00	01-Feb-00	09-Feb-00	13-Feb-00
17-Feb-00	18-Feb-00	19-Feb-00	12-Mar-00
14-Mar-00	27-Mar-00	18-Jun-00	28-Jul-00
30-Jul-00	31-Jul-00	06-Aug-00	07-Aug-00
13-Aug-00	08-Sep-00	24-Sep-00	10-Oct-00
30-Dec-00	31-Dec-00	14-Jan-01	28-Jan-01
16-Mar-01	29-Mar-01	03-Jun-01	04-Jun-01
05-Jun-01	06-Jun-01	15-Jul-01	22-Jul-01
02-Sep-01	07-Sep-01	25-Nov-01	26-Nov-01
14-Dec-01			

## SXT Aspect Sensor

The SXT aspect sensor comprised an objective group followed by the aspect sensor door, four filters in the forward filter wheel, and the CCD detector in the focal plane (Tsuneta *et al.*, 1991). The objective group included an entrance window with an attenuator coating, a bandpass filter, and a doublet lens. Technical details of the aspect sensor telescope are given by Grillot and Cruz (1990). The material properties of these optical elements are given in Table 3. All components were made of certified radiation resistant materials except for the Hoya CM-500. This glass was tested in a proton beam to 1000 times the expected three year dose at the anticipated *Yohkoh* orbit. All optical elements were anti-reflection coated.

**Table 3:** Aspect objective group components.

1	Synthetic fused silica plus 50 nm Al
2	Hoya CM-500 blue glass with far visible-near IR coating
3	Bi-convex Schott BK7-G18 (cerium stabilized) lens
4	Concave-convex LF5-G15 lens

**Figure 9:**
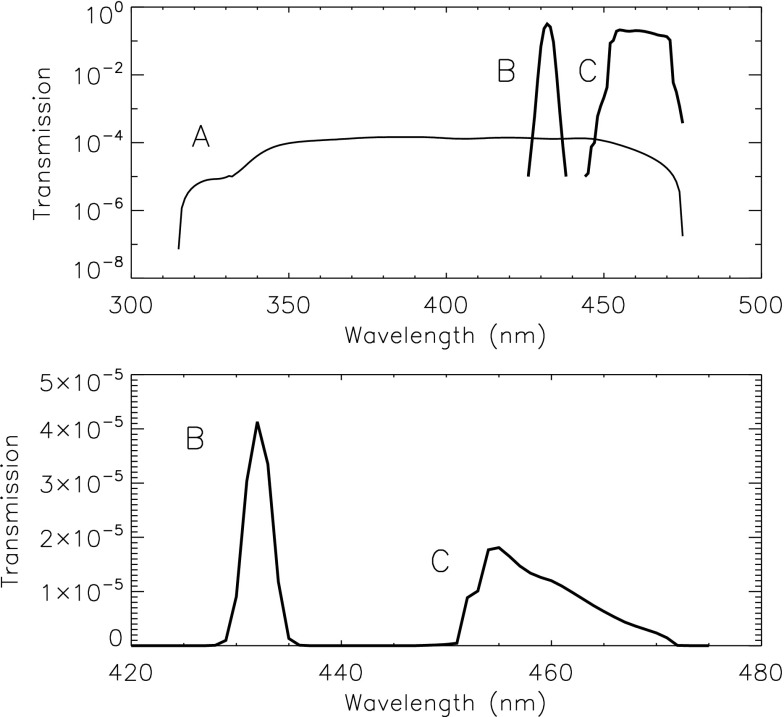
Transmission of aspect sensor optics. (A) lens assembly, (B) narrow-band filter, and (C) wide-band filter. Top panel: individual elements. Bottom panel: combined transmission.

The forward filter wheel of the SXT included four filters for use with the aspect sensor telescope. Their description is given in Table 4. The wide-band (WB) and narrow-band (NB) filters are equipped with 2.5 mm of Schott UG-5 absorbing glass on the rear side to reduce, to acceptable levels, ghosts from light reflected off of the surface of the CCD. Figure 9 presents the transmission and passbands of the optical assemblies of the SXT aspect sensor telescope.

**Table 4:** Aspect sensor filters.

1	Wide band (WB), 18.5 nm FWHM centered at 460 nm
2	Opal glass diffuser
3	Quartz defocusing lens for UV flood of CCD
4	Narrow band (NB), 3 nm FWHM centered at 431 nm

The relative alignment of the X-ray and optical images is discussed by Fuller, Lemen, and Acton (1994). The values given there were initially determined by Metcalf in 1992. The final calibration, given in the YLA and incorporated in all SolarSoft analysis software for SXT, was determined by Acton in 2008 using all available optical images. The absolute and relative offsets are given by the program *gt_sxt_axis.pro*. That is, the WB image falls $0.25\pm0.23$ full-resolution pixels east and $1.08\pm0.25$ pixels north of the X-ray axis. The NB image falls $0.90\pm0.23$ pixels east and $1.36\pm0.27$ pixels north of the X-ray axis. Offsets are in units of SXT full-resolution pixels of 2.455 arcsec.

### Aspect Signal Decline

**Figure 10:**
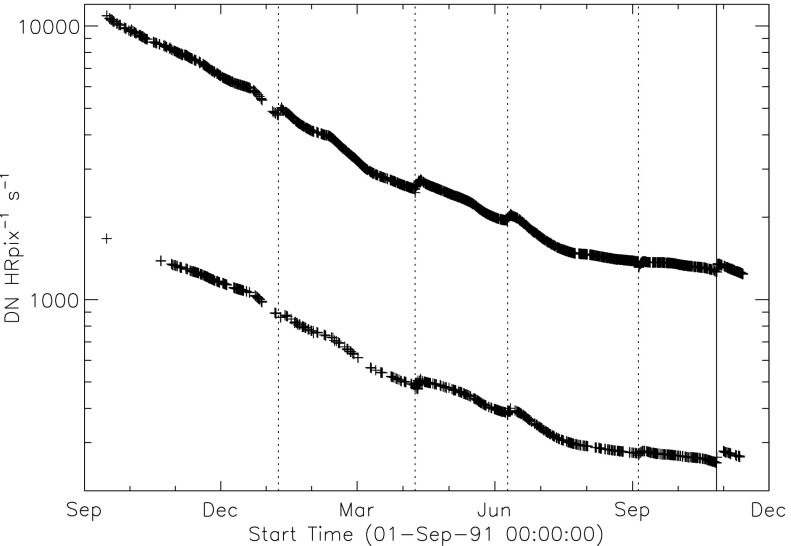
Average signal in $512\times512$ aspect sensor image. The upper curve is from the narrow band filter and lower curve from the wide band filter. Vertical dotted lines denote times of CCD bakeout. The vertical solid line indicates the time of the first failure (27 October 1992) of outer entrance sectors 6 and 7 (see Figure 4 where sectors are numbered) when a slight increase in optical signal appears. The termination of the curves falls on 13 November 1992 when the first inner entrance filter failed and the SXT was flooded with visible light.

It was quickly discovered following the launch of *Yohkoh* that the intensity of the optical images was decreasing approximately exponentially with time as illustrated in Figure 10.

**Figure 11:**
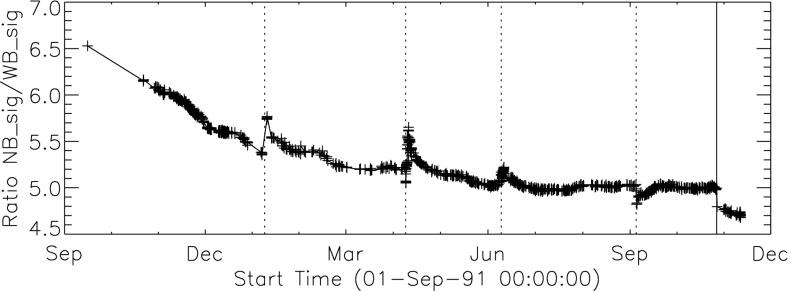
Ratio of NB to WB signal. Times of CCD warmup are indicated by dotted lines and 27 October 1992 entrance filter failure by the solid line.

Figure 10 shows that the NB and WB channels did not decay at exactly the same rate. This difference is better illustrated by the intensity ratio plot in Figure 11 which shows that the obscuring material was initially more absorbing at 431 nm than it was at 460 nm. As the layer became thicker the ratio stabilized around 5.0. For the first three CCD warmups the ratio increased (NB signal increased proportionally more) while for the fourth warmup the ratio decreased. This puzzling observation will be further discussed in the following two subsections.

#### Effect of CCD Warmup

**Figure 12:**
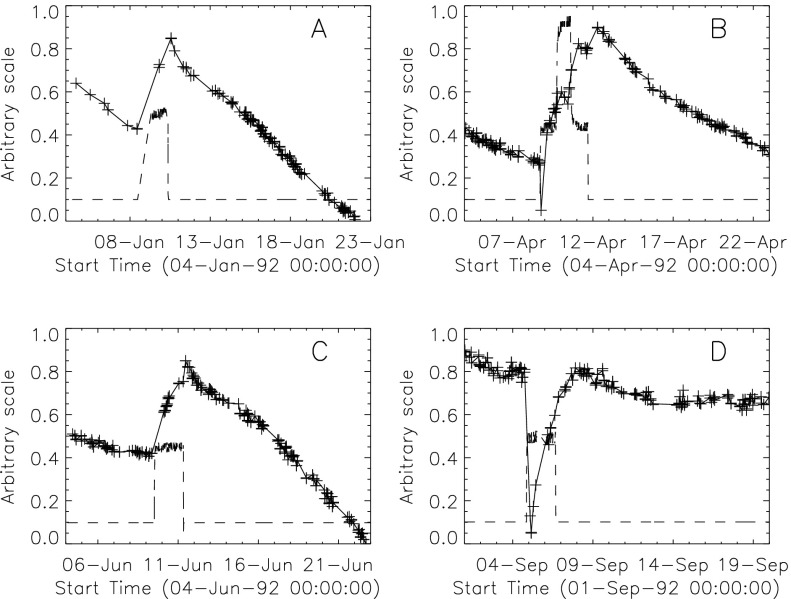
NB signal (+) and CCD temperature (broken line) for the four CCD warmups of 1992. Note that panel B illustrates a warmup (0 °C), bakeout (20 °C), and warmup (0 °C) experiment.

Figure 12 summarizes the response of the NB channel for the four CCD warmups in 1992. Note that for these warmups the increase in NB signal intensity continued for the duration of the warmup, as if an absorbing layer continued to evaporate throughout the interval. It does not appear that further increase in the bakeout temperature had much effect on the rate of evaporation as illustrated in Figure 12(B).

**Figure 13:**
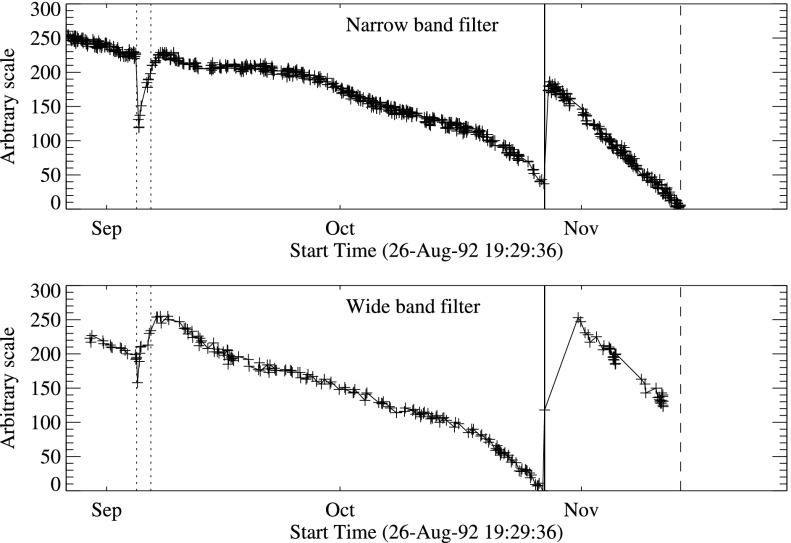
Detail of fourth CCD warmup. Dotted lines: CCD bakeout interval. Solid line: outer entrance filter failure. Dashed line: inner entrance filter failure.

The decrease in NB intensity at the fourth CCD warmup revealed in Figure 12(D) is very puzzling. As shown in Figure 13 the WB images show a quite different light curve. The effects observed here cannot, except perhaps for the first images after the beginning of CCD bakeout, be blamed on dark signal correction. Appropriate warm-CCD dark frames were used in preparation of these data. It is possible that interference effects in a thin layer of contaminant is involved as noted by Narukage *et al.* (2011) for the XRT instrument on *Hinode*.

Another feature seen in some of the aspect sensor light curves is a brief drop in signal at the very beginning of CCD warmup. This can be seen in the WB plot of Figure 13. The most probable cause is that the CCD warms up slower than the CCD camera temperature indicator so that, for early images, the software chooses dark frames, taken later in time, that are too warm, thus over-correcting the exposures. Due to the higher dark current and less accurate dark-correction, bakeout images are, in general, less accurate photometrically.

#### Uniformity of Signal Change over the CCD

**Figure 14:**
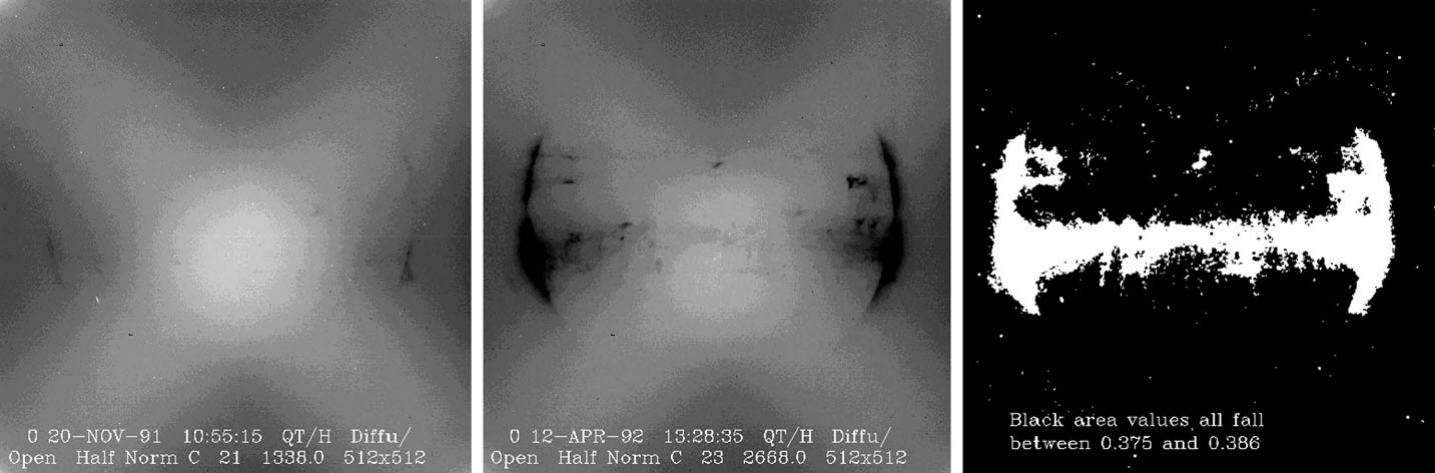
SXT diffuser images obtained 20 November 1991 (left) and 12 April 1992 (center). X-ray damaged area is evident in the center (later) image. In the rightmost panel the black portion shows the area of the CCD in which the signal decrease is very uniform, falling between 61.4 and 62.5 %.

If the decrease in visible-light signal is uniform all over the CCD this would indicate that the primary cause is in front of the focussing optics. That is, probably an absorbing layer on the front of the aspect sensor entrance window, or a uniform coating all over the CCD. As demonstrated in Figure 14 this appears to be the case.

When illuminated by the out-of-focus solar image coming from the aspect sensor optics the opal glass diffuser provides an approximately uniform illumination of the CCD. This provides a means for identifying possible areas of non-uniformity of optical response.

The results of such a comparison of SXT diffuser images, chosen to be near identical *Yohkoh* pointing, is illustrated in Figure 14. There is a notable decrease in optical response in areas (active region belt and the limbs) that were heavily irradiated by soft X-rays. However, the ratio image on the right shows very little departure from uniformity outside of the white area. In the areas indicated in black the average late/early signal ratio is $0.380\pm0.004$. The decrease in CCD sensitivity in radiation-damaged areas is another issue that is discussed in Section 9.

In order to check for uniformity of response decrease within the solar image the mean intensity in $10^{\circ}$ sectors between 0.8 and $1.05~R_{\odot}$ at the north, west, south, and east points of the disk have been compared. The signals from the four points around the limb decrease in lock step, again demonstrating that the absorbing agent is uniform over the field of view.

In order to determine if scattering of the optical light changed during the period of the mission prior to November 1992 we compared the signal in an annulus surrounding the narrow-band image with the disk-center signal. We find that the scattering of sunlight into the near-Sun above-limb annulus is constant up until 27 October 1992 when the first entrance filter failure occurred. The stability of the above-limb/disk-center ratio for more than a year establishes that whatever caused the response decrease of the aspect sensor signal did not increase the visible-light scatter of the aspect telescope.

All indications from these image uniformity analyses demonstrate that absorbing material must have been deposited on optical elements in front of the focussing lenses, on the optical elements of the forward filter wheel, or uniformly over the face of the CCD. It seems likely that any hydrocarbon-based absorbing layer on the CCD thick enough to reduce the aspect sensor signals by nearly a factor of 8.8 (an optical thickness of 2.2 at 430 nm) would have decreased the soft X-ray signal more than can be inferred from the X-ray data, although optical signal variations in sync with CCD warmups (Figures 10 – 13) indicate that detector contamination may have contributed to a minor degree. Heavy contamination of the optical elements of the forward filter wheel (Table 4) is ruled out by the fact that their transmission exhibited total stability as soon as the light was coming off of the X-ray mirror rather than through the aspect sensor optics. Thus, I conclude that a change in the transmission of the aspect sensor lens assembly is to blame for the majority of the signal decrease with CCD contamination playing a secondary role.

### Aspect Sensor Performance Summary

The aspect sensor light curves displayed in Figure 10 are not perfectly smooth, even apart from discontinuities at the times of CCD bakeout. A comparison with season (solar diameter), the temperature of the SXT forward support plate (which holds the SXT objective group including the aspect sensor optics), and epochs of heightened energetic particle fluxes from the Van Allen radiation belts revealed no obvious correlation with the loss of aspect sensor sensitivity.

Signals through all optical filters showed a similar decline and loss of signal. That is, between 15 September 1991 and 13 November 1992 the NB and diffuser signals decreased by factors of 8.8 and 9.1, respectively. Between 15 September 1991 and 27 October 1992 the WB signal decreased by a factor of 6.6. The decay curve was essentially the same for all optical images: narrow band, wide band, diffuser, or quartz CCD-flood lens. The decay of signal was uniform over the entire CCD. The CCD showed no effects (*e.g.*, from radiation damage) correlating with the decline. The optical elements were all selected for radiation insensitivity so radiation-induced color centers in the optics are unlikely to contribute to the decrease in transmission. All of these facts lead to the conclusion that the optical signal decay was caused by the accumulation of some absorptive or reflective contaminant, reaching an effective optical thickness of about 2.2 by November 1992, on or within the aspect objective lens assembly. It is true that a small fraction of the decrease is caused by ionizing-radiation damage to the CCD in localized areas but this effect accounts for only about 1 % of the total decrease in optical signal.

The small increases in optical signal at times of some CCD bakeout (Figure 10) probably indicate the removal of a small amount of absorbing contaminant from the face of the CCD.

We have not been able to determine what material could have deposited on or within the aspect sensor optics with such a large optical thickness. The thermal shield mounted to the space craft in front of the SXT (Figure 6) and the inside of the aspect sensor optical assembly itself were painted with black Chemglaze Z306, applied and baked according to NASA specifications. After more than two decades the mystery remains.

## Entrance Filter Failures and Visible Stray Light

The most serious instrumental anomalies during SXT in-flight operation were periodic failures of the entrance filters in front of the X-ray mirrors. The associated flood of sunlight into the telescope contaminated X-ray images and precluded collection of any aspect telescope images. Fortunately, it proved possible in large measure to subtract this stray-light signal from the X-ray data. Because of the importance of these effects to the scientific return of the SXT the following sections will present a detailed description of the stray-light effects and their remediation.

It has not proven possible to ascertain the exact cause(s) of these failures. As shown in Figure 6 the filters are quite well protected with a very small area and solid angle viewing space. Micro-meteorite impact cannot be ruled out but seems unlikely to account for so many failures. Most of the failures have appeared with the first exposure following orbit night so it seems likely that a cumulative degradation of the Lexan plastic film due to thermal stress at the day–night and night–day transitions may be a factor in the filter ruptures.

**Figure 15:**
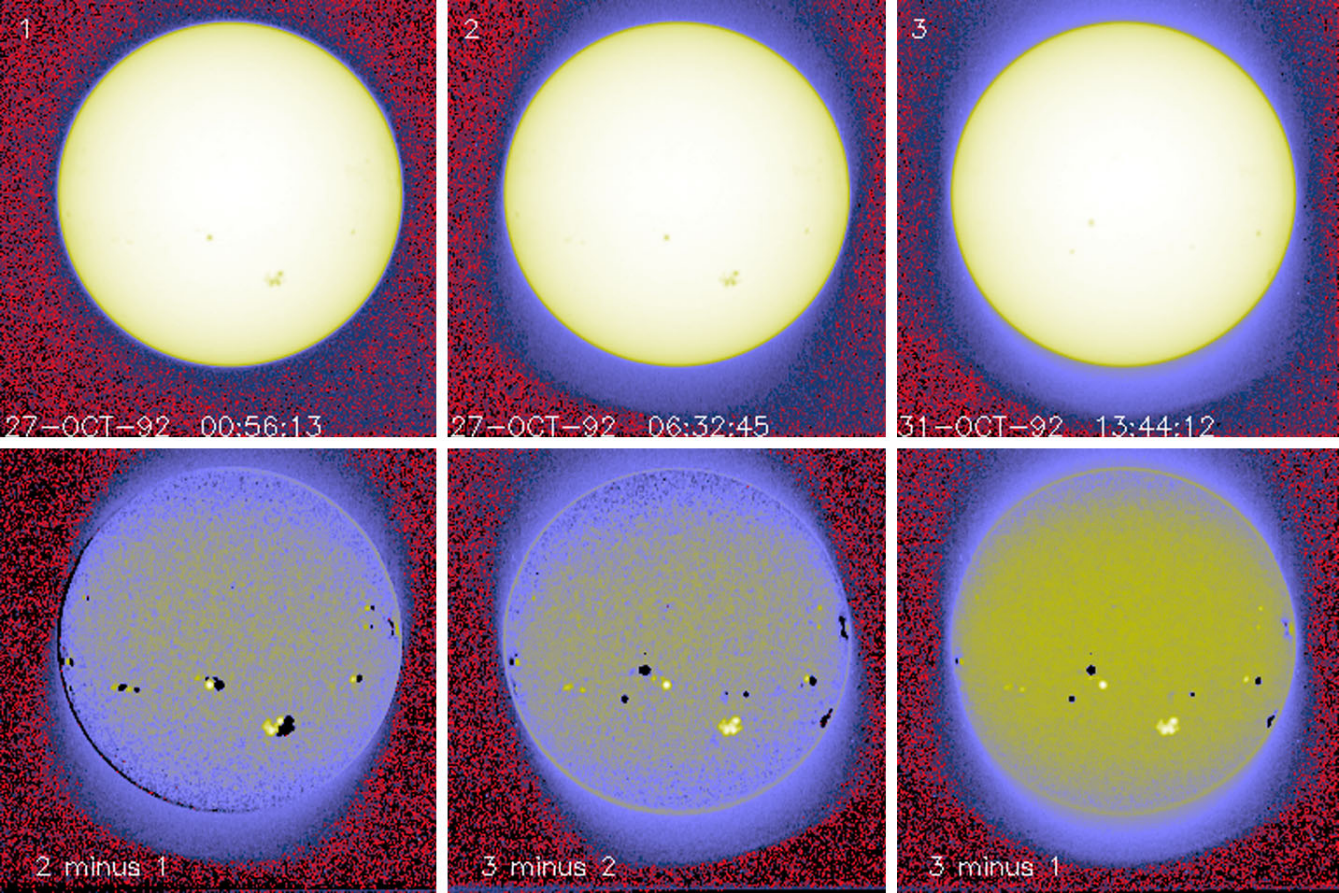
SXT wide-band aspect sensor images. The top three panels are images acquired (1) before the 27 October 1992 failures, (2) after the first failure, and (3) after the second failure. The poor on-disk contrast of these three images is caused by the logarithmic scaling. The bottom three panels are difference images detailing the effects of each failure.

**Figure 16:**
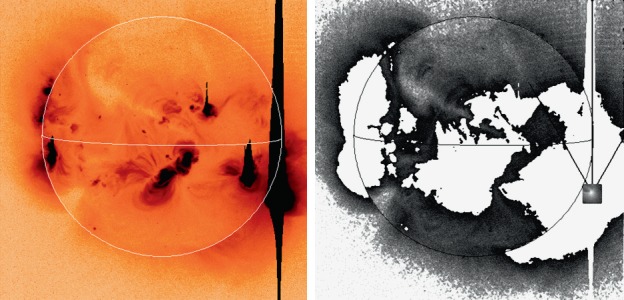
SXT 5.3 s AlMg exposure (2 November 1992, 13:24:42 UT) showing enhanced X-ray scatter in a $60^{\circ}$ sector directed northwards. Solar limb and equator drawn in for clarity. Left image is a reversed red color table, black areas show CCD saturation and charge bleed. Right image has been scaled to emphasize the striped scattered X-ray pattern northwards on the image in two $30^{\circ}$ sectors, indicated by solid lines. A nearly simultaneous 0.5 s Be-filter PFI has been inserted to show the position of the bright X-ray kernel well above the limb.

As noted in Section 4 visible light was excluded from the X-ray telescope by dual metal-coated thin plastic filters in front of the X-ray mirror. The first catastrophic failure occurred at 05:59: UT, 27 October 1992, about 13 months after launch. The adjacent outer entrance filter segment failed 79 min later. With the exception of the very first failure, most of the failures were first detected at orbit sunrise. For the single case in 1992, when we were able unambiguously to discriminate between failures of outer and inner filters, an inner filter failed shortly (17 days) after the outer filters. For the outer filters the Lexan was protected from space degradation by the Al and Ti coatings. After the outer filters failed the Lexan plastic of the inner filters was directly exposed to degradation by solar UV and atomic oxygen (see, *e.g.*, Dever *et al.*, 2012). Typically, as evidenced by Figures 15 and 16, when the filters failed the entire $30^{\circ}$ sectors opened. This is consistent with experience with pre-launch testing of these filters. In the 27 October 1992 cases both failed sectors were mounted on a common frame.

The resulting visible stray light affected all subsequent X-ray exposures to some degree and has multiple important impacts on SXT performance as listed in Table 5. The remainder of this section details our diagnosis of stray-light effects and steps taken to correct the X-ray data for stray-light contamination.

**Table 5:** Impacts of SXT entrance filter failures.

1	Light of all wavelengths enters the telescope
2	The SXT optical aspect telescope becomes unusable
3	The X-ray sensitivity of the instrument increases
4	The X-ray spectral response of the instrument is changed
5	The pattern and intensity of stray visible light appearing on X-ray images varied with mission epoch, with pointing, and was different for each SXT analysis filter
6	Pinholes in, and stray-light transmission through, the thinnest (Al.1) analysis filter cannot, in general, be corrected well enough for accurate quantitative analysis of quiet coronal features obtained through this filter after 13 November 1992
7	Solar ultraviolet (UV) light becomes available for CCD photon flood

The SXT aspect telescope is a highly filtered bandpass telescope so the direct entry of unattenuated sunlight into the SXT completely overwhelmed the optical aspect image. The SXT X-ray sensitivity and spectral response are determined in part by the X-ray transmission properties of the entrance filters so each removal from the optical path increases somewhat the X-ray sensitivity of the telescope, particularly at the longer wavelengths. These changes in X-ray response are not evident to the unaided eye in the images and, to the extent that we understand the chronology of failures, the SXT analysis software takes account of the changes in spectral response.

The seventh effect listed in Table 5 is arguably a benefit. Flooding the CCD with UV light helps to anneal soft X-ray damage to the device (Acton *et al.*, 1991). The fact that the SXT detector survived in usable condition for over a decade may be due, at least in part, to the morning UV flood strongly enhanced by the sunlight entering through the failed entrance filters.

### First Entrance Filter Failure

As a result of failures of the thin-film entrance filters stray visible light began to enter the telescope on 27 October 1992. On 13 November 1992 the second layer of one of the duplex entrance filters failed and the full solar spectrum entered the instrument by reflection off of the X-ray mirror. Aspect sensor images could no longer be acquired, even the shortest exposures were totally saturated.

#### Visible Signal Change

In late October 1992 SXT observers noted a faint increase in background intensity in solar images taken through the SXT aspect sensor. Marilyn Bruner, designer of the SXT telescope, first suggested that this might be caused by the failure of an entrance filter. As Figure 15 illustrates, the stray-light pattern is what would be expected by the failures of one, and soon thereafter a second, $30^{\circ}$ filter sector. The fact that the stray-light increase is so modest attests to the fact that the inner filters were still intact. Pre-launch testing revealed that a single entrance filter transmits about $10^{-6}$ in the visible.

The sequence and location of these first entrance filter failures is demonstrated in Figure 15. Visible light focused off of the X-ray mirror is severely diffracted in the radial direction (0.362 mm aperture) but much less in the circumferential direction where the effective aperture is much wider (${\approx}\,53~\mbox{mm}$, slightly curved). Thus, the rightmost difference image, poorly focused at one and seven o’clock and well focused at four and ten o’clock, shows that either sector 1 or 7 (see Figure 4 for sector numbers) had opened up. The middle difference image shows that the adjacent sector 6 or 12 subsequently also failed.

Failure of SXT entrance filters increases the instrument sensitivity at long X-ray wavelengths because of the removal of absorbing material in the optical path. However, the interpretation of the stray-light monitor variations (Section 7.3) in terms of changes in X-ray sensitivity is tricky because of the duplex entrance filter design of the SXT. The opening up of a filter sector in a single entrance filter layer will increase X-ray sensitivity. However, except for this early case, both layers must open to register the change in stray light. Thus, after the first inner filter failure of 13 November 1992 the enhanced stray-light signal from further outer filter failures was overwhelmed by the signal from sectors already having double-filter failures.

#### X-ray Signal Change

X-ray scattering from surface micro-roughness of a grazing incidence X-ray optic is predominantly in the axial direction, *i.e.*, perpendicular to the mirror surface (Aschenbach, 1985). The X-ray scattering wings of the SXT point spread function appear in exposures of intense solar flares (Figure 1) – in this case a useful diagnostic of entrance filter failures. When an entrance filter sector fails the X-ray signal incident on the mirror through that sector increases. Thus, the scattered X-rays in the image direction opposite to that sector are enhanced relative to other directions. This happened on one well-observed occasion between 27 October 1992 and 13 November 1992 and is illustrated in Figure 16. This AlMg image was taken late in a GOES class X10 flare when the intensity had diminished to a C9 level.

The $180^{\circ}$ ambiguity of which sectors failed 27 October 1992 is removed by this flare image. Figure 16 shows a scattered X-ray pattern to be enhanced in two sectors on both sides of the upward vertical (north). The faint arcs in the scatter area are the shadows of the AlMg filter support mesh. The inset is a Be-filter PFI obtained 1 min after the FFI. It is interesting that at this late epoch in this great flare the soft X-ray emission is concentrated in a small kernel at the top of a single high loop, like the diamond on a ring. The direction and width of the scatter demonstrates conclusively that the outer entrance filters of sectors 6 and 7, both sections of the filter frame nearest to the instrument baseplate, were the failed sectors.

**Figure 17:**
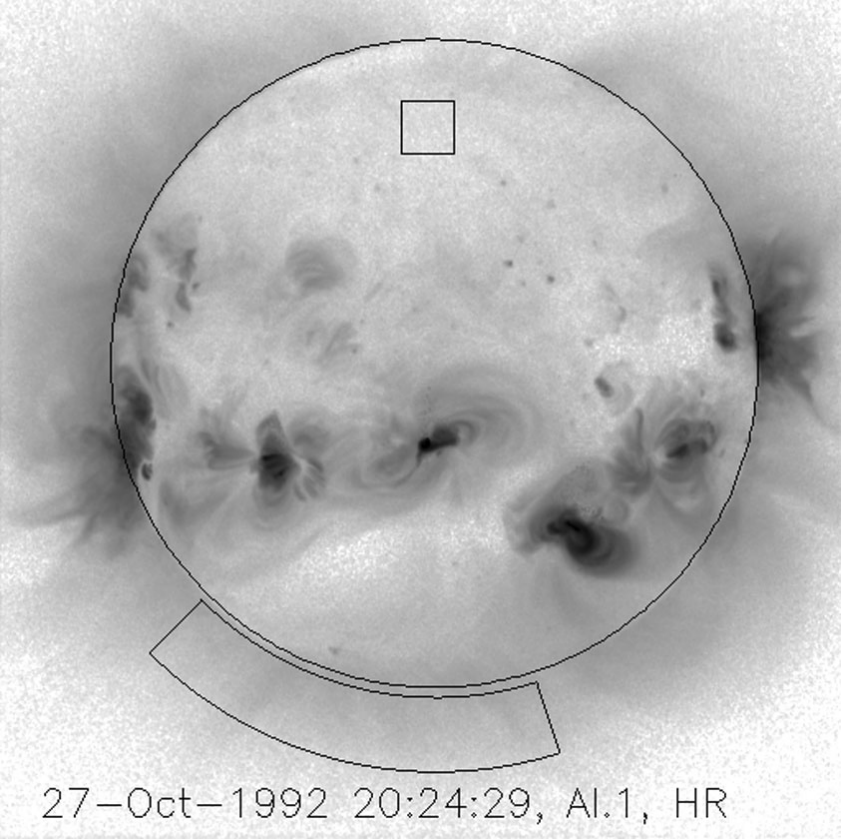
South polar and northern high latitude areas sampled for filter-failure analysis.

Because of the spectral dependence of entrance-filter transmission the coolest coronal regions (lower energy photons) should show the greatest signal increase. Figure 17 illustrates quiet regions in the north and south chosen for examination for changes in X-ray signal levels associated with the 27 October 1992 failures.

Only composite full Sun images outside the South Atlantic Anomaly (SAA) were used in the analysis so that approximate correction for scattered X-rays could be made with the SolarSoft program *sxt_decon.pro*. As this was a period of moderate X-ray flare activity care was taken to ensure that there was no correlation between GOES flux and the SXT signal in the selected area to avoid as much as possible signal enhancements associated with transient X-ray emission. Signals in these regions were sufficiently intense to minimize error in correction for X-ray scatter and also avoid the newly asymmetric scatter to the north shown in Figure 16.

**Figure 18:**
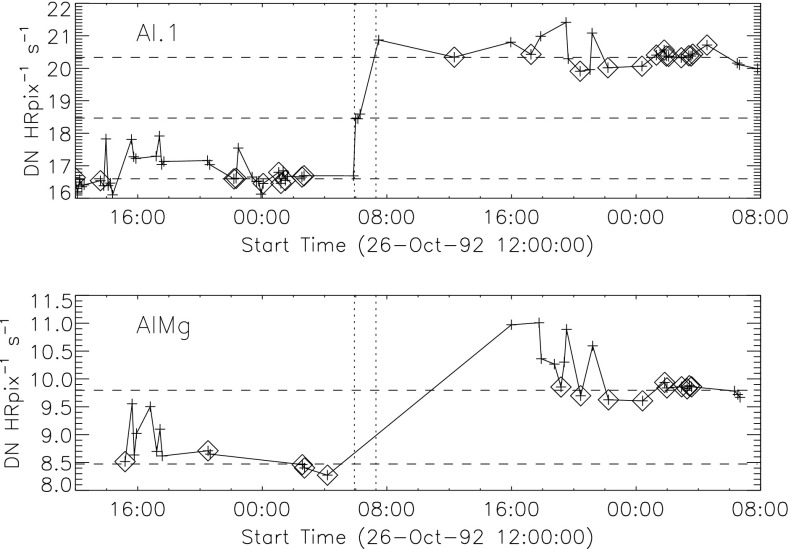
Increase in SXT signal level through the two thin analysis filters, Al.1 and AlMg, for the south pole sector of Figure 17. Dotted lines indicate the approximate times of the ruptures. Diamonds identify the data used in computing the expected signal increase.

**Figure 19:**
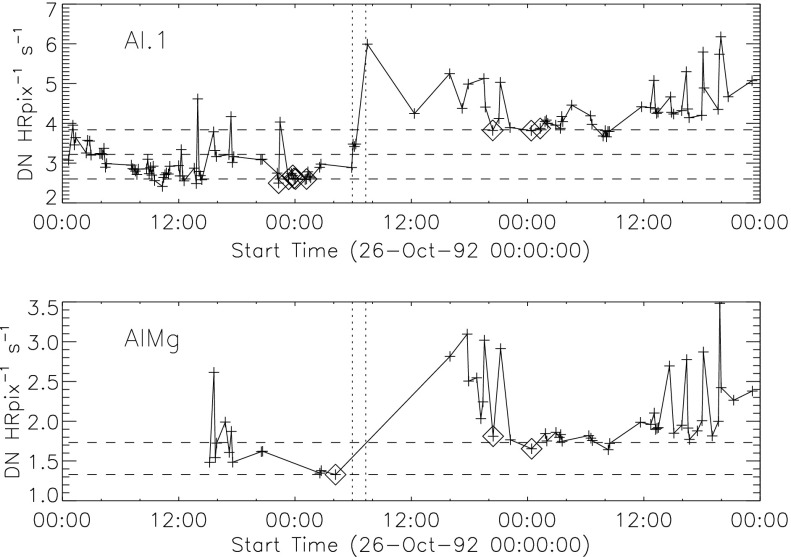
Increase in SXT signal level through the two thin analysis filters for the north high latitude region of Figure 17. Dotted lines indicate the approximate times of the ruptures. Diamonds identify the data used in computing the expected signal increase.

The signal level changes for the two thinnest SXT analysis filters are shown in Figures 18 and 19. The horizontal broken lines display the average signal levels of the data points chosen for analysis before and after the entrance filter rupture. The center broken line in each upper graph is the average of the upper and lower signal levels. For the bright south polar sector it falls precisely through the group of images acquired after the first and before the second rupture events, as would be expected if our interpretation is correct. The data from the northern high latitude region is not as definitive, probably due to weaker signals and contamination from transient X-rays.

The crosses present data from all usable images. The diamonds are the images for which the exposures were taken more than 2 min after sunrise and before sunset, for which *Yohkoh* was not in the SAA, and for which the GOES low-channel (1 – 8 Å) signal falls between $1.0\,\mbox{--}\,1.7\times10^{-6}~\mbox{W}\,\mbox{m}^{-2}\,\mbox{s}^{-1}$. For the relatively bright south pole region the Al.1 signal increased by a factor of 1.22 and the AlMg signal by 1.16. For the faint high latitude region in the north a more severe constraint on GOES signal of $0.8\,\mbox{--}\,1.3\times10^{-6}~\mbox{W}\,\mbox{m}^{-2}\,\mbox{s}^{-1}$ is used in the image selection criterion. For the north region the Al.1 signal increased by a factor of 1.46 and the AlMg by 1.30.

**Figure 20:**
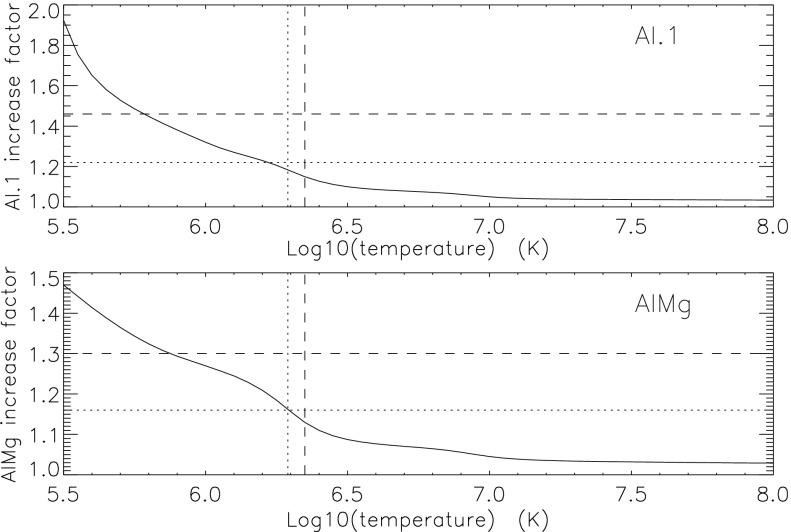
Predicted signal increase *versus* coronal temperature resulting from a $60^{\circ}$ entrance-filter rupture (*i.e.*, two filter sectors) of a single entrance filter layer for the SXT thin analysis filters. These curves utilize Chianti (version 3.03) with Mazzorta (Mazzotta *et al.*, 1998) ion equilibrium and Meyer (Meyer, 1985) abundances. The horizontal lines show the observed changes and the vertical lines the derived pre-failure filter-ratio temperatures. The dotted lines show the results for the above-limb south polar region and the dashed lines for the northern coronal hole region. (Sampled regions are indicated in Figure 17.)

To test our interpretation of the impact of the 27 October 1992 filter failures we used standard SXT analysis software to compute the fractional SXT signal increase *versus* coronal temperature. That is, for a given emission measure the signal increase caused by removal of an entrance filter will be greater for a lower temperature corona because of the preponderance of lower energy photons, which are more strongly absorbed by the entrance filter. For temperatures above about three million K the loss of a single entrance filter makes very little difference as shown in Figure 20. The results of this computation are displayed in Figure 20 along with derived results from Figures 18 and 19. A positive result would have the dotted (dashed) lines crossing on or near the solid curve in each panel. For the south pole region (dotted lines) the analysis results are consistent with the result expected for the opening of two $30^{\circ}$ outer-entrance-filter sectors. Note that these results are quite insensitive to the atomic spectral model used.

The comparable results for the weaker-signal north high latitude coronal hole region (dashed lines) are in poor agreement with the calculations. The observed signal increase is larger than expected from the analytical model. This effect may be caused by poorer counting statistics (resulting from both weaker signal and much smaller area sampled) and/or a relatively greater contamination from transient X-ray activity.

The conclusion from this analysis is that the change in sensitivity and temperature response of SXT following the 27 October 1992 entrance filter failures is consistent with expectations for the total opening of two (numbers 6 and 7) outer entrance filters sectors. The results also confirm the accuracy and applicability of SXT calibration and data analysis software.

### Stray Light Paths to the CCD

We are able to follow SXT stray-light evolution in three ways: the stray-light monitor, diffuser images, and terminator images.

SXT stray-light monitor images are obtained with the shutter closed, either the narrow-band (NB) or wide-band (WB) optical filter in place in filter wheel A, and filter wheel B in the open position. Diffuser images use the shutter and have the opal glass diffuser in place in filter wheel A with filter wheel B in the open position. Terminator images are obtained between 24 and 12 s before ephemeris sunset, a period when the light path is opaque to soft X-rays but transparent to visible light. Any one of the X-ray analysis filters in filter B may be used for terminator images. Filter wheel A is always in the open position for terminator images.

The stray-light path to the CCD is totally different for the normal X-ray images and the shutter-closed stray-light monitor images. For the case of the X-ray images the highly diffracted, poorly focused, visible image from the X-ray mirror falls upon the X-ray analysis filter in filter wheel B. Depending upon the nature of the filter surface a certain fraction of this bright light is scattered or reflected forward through the open filter hole in filter wheel A and illuminates the inside of the forward filter wheel aperture plate around its entrance hole or travels further forward into the body of the telescope.

**Figure 21:**
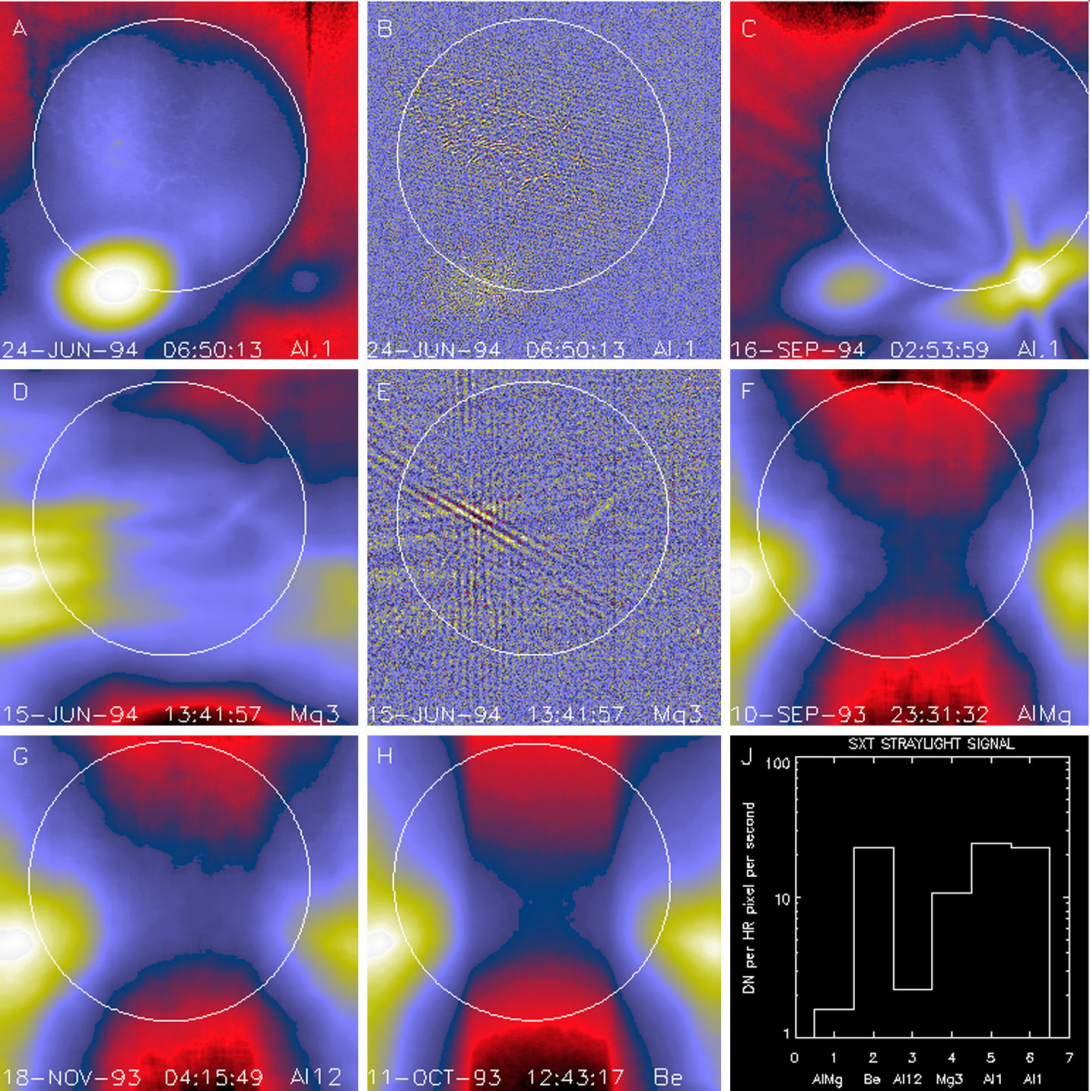
Stray-light pattern for each of the SXT analysis filters. The location and size of the solar disk is indicated by the circles. Two examples are given for Al.1 to demonstrate the effects of pointing on the Al.1 stray-light pattern. These two images (A and C) are displayed with logarithmic scaling. All of the others are scaled linearly. Image B is flattened and clipped to show some of the fine scale structure of the Al.1 stray-light image. Panel E is a correspondingly processed image for Mg3. Panel J illustrates the relative strength of the stray-light signal for each of the filters. The rightmost Al.1 datum (#6) of panel J corresponds to the image in panel C. AlMg, the filter least affected by stray light, is used, with the shutter closed, for SXT dark-frame exposures. Filter designations are given on the lower right corner of each panel.

The CCD can see the illuminated filter wheel housing aperture plate through the triangular weight-relief cut outs on each side of all filter mounting positions in filter wheels B and A. This is where the light comes from that produces the characteristic patterns along the east and west sides of the CCD image for open-shutter X-ray exposures (see Figure 21). The patterns and relative intensity of stray visible light on the CCD for the different X-ray analysis filters is illustrated in Figure 21.

This interpretation can explain not only the pattern but the variation in stray-light level for the different analysis filters. Figure 21 shows a great variation of stray-light intensity from filter to filter, but with nearly the same basic structure (except for the special case of Al.1). The intensity differences can be traced to the reflective and diffuse-scatter properties of the filters as described in Table 6.

**Table 6:** SXT analysis filters.

Filter	Components	Description
Al.1	Al(126.5 nm) + Si(1.3 nm) + Al_2_O_3_(0.3 nm) + SS^1^ mesh(0.84 %)	Shiny, transmits ≈ 10^−6^, pinholes
AlMg	Al(293 nm) + Mg(207 nm) + Mn(56.2 nm) + C(19 nm) + Si(2.9 nm) + SS mesh(0.83 %)	Shiny, reflects more than scatters
Be	Be(119 μm)	Diffuse scatterer
Al12	Al(11.6 μm) + SS mesh(0.83 %)	Shiny, reflects more than scatters
Mg3	Mg(2.52 μm) + SS mesh(0.78 %)	Uneven surface, scatters

^1^Stainless steel.

Note that, except for Be, the filters are not precisely flat so the detailed scattering/reflecting properties will be unique for each part. These conclusions are consistent with inspection of flight-spare filters and how the filters were installed in filter wheel B. Small variations in the stray-light pattern reflect differences in *Yohkoh* pointing and/or seasonal changes of the angular diameter of the solar disk.

For the case of shutter-closed stray-light monitor exposures the poorly focused visible light image from the X-ray mirror first passes through either the NB or WB optical filters (passbands shown in Figure 10) and on to the closed shutter blade. The shutter being closed, the CCD cannot see the filter wheel cutouts and the forward aperture plate as is the case for shutter-open exposures. The aperture between the shutter blade and the CCD is a 24.8 mm square with 3.2 mm radius rounded corners. The CCD sensitive area is an 18.43 mm square centered within this aperture.

The shutter-closed stray-light path into the CCD volume may be a result of the fact that the shutter does not quite cover the two lower corners of the square aperture in front of the CCD. Careful checking of drawings against an actual spare shutter shows about 1.7 mm overlap on top center and essentially 0.0 mm at the top corners. However, the bottom corners of the aperture plate are not quite covered in either shutter-closed position, leaving a gap about $1~\mbox{mm}^{2}$ in area. As shown in Figure 24 there are two stray-light monitor states, separated by about a factor of two in intensity, depending on whether the wide gap in the rotating shutter is on one side or the other of the CCD aperture. The high states are associated with shutter-encoder position 26 and low states with encoder position 30. These two positions are $90^{\circ}$ apart, centered on the open position.

**Figure 22:**
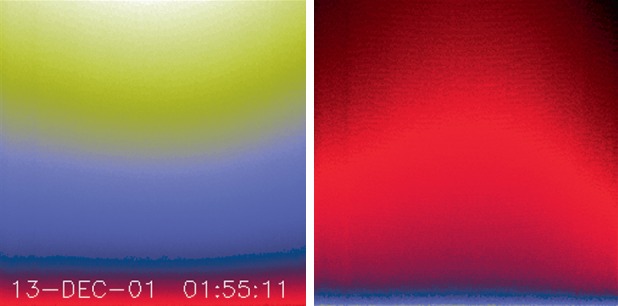
Left: WB stray-light monitor image (high state). Right: same image corrected line-by-line for exposure time to approximate the actual distribution of stray light on the CCD. The color table (IDL STERN SPECIAL, red:faint to white:bright) has been chosen to emphasize the difference between the two images.

**Figure 23:**
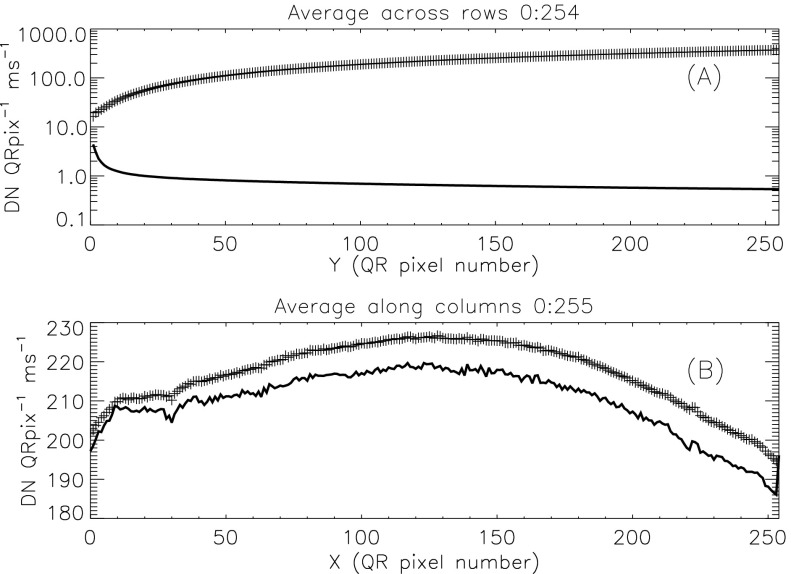
Stray-light signal. Crosses refer to the averaged signal in the actual stray-light image. The solid curves show the distribution of stray light on the CCD, corrected for the accumulation time of each row. (A) illumination in the column ($Y$, sum over $X$) direction. (B) illumination in the row ($X$, sum over $Y$) direction. In (B) the solid curve has been multiplied by 285 to facilitate comparison of the two curves.

The illumination is, within about 2 %, the same across the CCD for both shutter positions. In the high state the signal goes from 2.1 to 1.8 times the low-state value from the bottom to the top of the CCD image. Figures 22 and 23 reveal that the illumination of the CCD by the light leak is strongly concentrated along the bottom (serial register side) of the CCD. This may account for the fact that there is no sign of the radiation-damaged, low visible-light sensitivity, features in the image – even though they are quite prominent in the diffuser images. Note that the stray-light distribution across the CCD is fairly flat but concentrated toward the center. This seems to contradict the interpretation that the light enters the extreme corners of the CCD aperture plate. In any case, the pattern of stray light is well defined in CCD dark frames and available for subtraction from X-ray images.

### Stray Light Monitor

Beginning early December 1992 a routine stray-light monitor image was added to the standard FFI observing table. These were quarter resolution (QR) shutter-closed exposures using the shortest data processor exposure ($\mathrm{DPE}=2$, 7.91 ms) with filter A in position 2 (NB) and filter B in position 1 (open). In 1999, after the three entrance failures early in the year, the standard stray-light monitor exposure was approaching saturation so we began to take stray-light monitor exposures using filter A position 5 (WB). Both NB and WB exposures were taken for about a year for cross calibration. The conversion is $\mathrm{NB}=3.8*\mbox{WB}$ for dark-frame corrected images. NB images ceased to be routinely taken on 4 April 2000.

A sample stray-light monitor image is shown in the left hand image of Figure 22. The right hand image has been corrected for dwell time per row during CCD read out. Note that the stray light is concentrated toward the bottom edge of the CCD.

Figure 23 illustrates, from one dimensional sums over the stray-light image of Figure 22, the distribution of stray-light illumination on the CCD in a more quantitative fashion.

**Figure 24:**
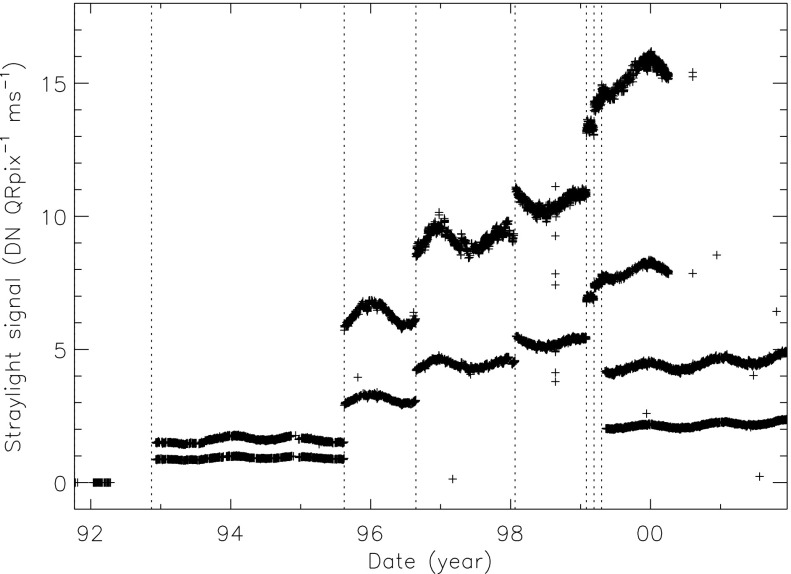
Signals from stray-light monitor exposures. The vertical dotted lines denote times of known entrance filter ruptures (Table 8). The two parallel curves correspond to the two possible shutter-closed positions, called high state and low state in the text. The low pair of curves beginning April 1999 use the less transmissive wide-band (WB) filter in filter wheel A. The $\mbox{ms}^{-1}$ unit of the Y-axis refers only to image dwell (‘shutter’) time.

Figure 24 displays the total dark-corrected stray-light signal from 11,885 stray-light monitor images. To some degree the stray-light levels are affected by *Yohkoh* pointing and seasonal solar diameter. The data points shown for 1991 and 1992 are from exposures equivalent to the stray-light monitors that were obtained as dark-frame candidates. They average to zero, *i.e.*, there was no visible stray light reaching the CCD prior to the entrance filter failures.

**Figure 25:**
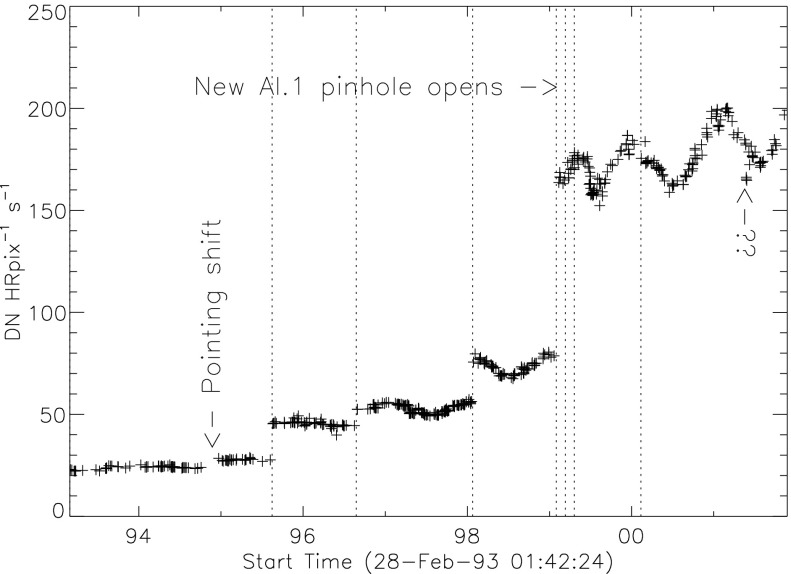
Summed signal in centrally pointed Al.1 terminator (SFC) images. Signal levels are sensitive to *Yohkoh* pointing.

The stray-light monitor exposures are taken with the shutter closed, an optical filter in place in the filter A position, and filter B in the open position. While a useful way to monitor the total stray light entering the instrument the configuration is quite different from that of the X-ray exposures, terminator leak images or diffuser images. For X-ray images filter A is normally in the open position, an X-ray analysis filter in filter B, and the shutter is open. Figure 25 illustrates the total signal in centrally pointed Al.1 terminator FFIs (terminator images are described in Section 7.5). Comparison to Figure 24 shows that, near the end of January 1999, there is a proportionally much larger step in the Al.1 stray-light signal than in the stray-light monitor signal. This is caused by a near-simultaneous entrance filter failure and the opening of a new pinhole in the Al.1 analysis filter, probably from the enhanced thermal shock.

### The SXT Diffuser

An opal glass diffuser was installed in the forward filter wheel of the SXT for use in generating CCD flat-field images on a routine basis. However, the diffuser images were by no means ‘flat’ and also recorded visible light artifacts not present in the X-ray images (see, *e.g.*, Figure 14). Thus the diffuser images were of limited use for their original purpose.

Contrary to the stray-light monitor images discussed in Section 7.3, for which the path of stray light to the CCD involved scattering and leakage past the shutter, the diffuser images are normal, shutter controlled, exposures – even after the entrance filters failed and the bulk of the light came off the X-ray mirror rather than through the aspect sensor. This makes the diffuser images better quantitative indicators of stray-light levels onto the SXT focal plane than the stray-light monitor. Furthermore, unlike the wide and narrow band optical images, the diffuser images were not saturated.

**Figure 26:**
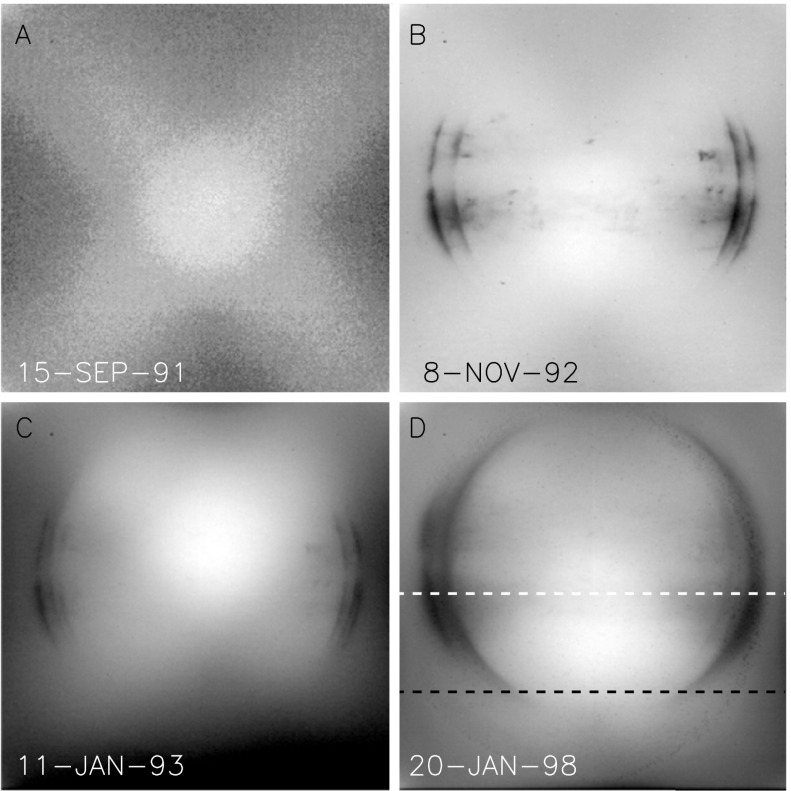
Examples of diffuser images throughout the mission, individually scaled to reveal details. (A) first diffuser image. Noisy because of eight bit compression. (B) shortly before 13 November 1992 entrance filter failure. Damage from X-ray exposure is evident. (C) two months after filter failure. About 8 % of X-ray entrance ring is open. (D) five years after (C). About two-thirds of X-ray entrance ring is open by this time. The image section between the broken black and white lines denotes the area used for producing the mission-long light curves of Figures 28 and 29.

**Figure 27:**
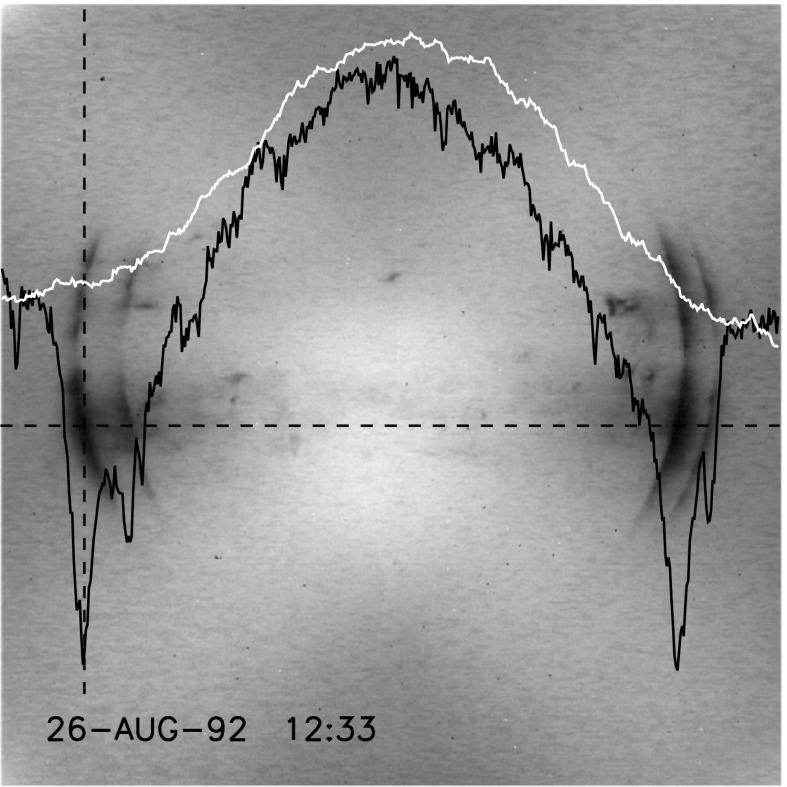
Demonstration of decrease in CCD optical sensitivity caused by soft X-ray damage after nearly one year of use. Image scaled to reveal details. Curves are described in text.

Figure 26 illustrates the evolution of the diffuser images with time. Up until the November 1992 entrance filter failure the major artifacts were the loss of optical sensitivity in the strongly X-ray irradiated areas. This impact is quantitatively illustrated in Figure 27. Here, the horizontal broken line denotes the CCD row chosen for the two intensity curves. The black curve is from this image. The upper (white) profile from the diffuser image of 15 September 1991, 17:55 UT, before any radiation damage had accumulated, has been normalized to the 26 August 1992 profile at the left and right wings of the image. At the column indicated by the broken vertical line, through the deepest part of the damaged area, the sensitivity to light coming through the SXT aspect sensor optics is decreased by 20 %. This is in rough quantitative agreement with Figure 26(B), which was obtained a bit over two months later. For comparison, the burned-in areas of Figures 26(C) and (D) are only about three and four percent, respectively. The reason for this relative improvement in optical sensitivity probably has to do with the change in short wave cut off of the every-orbit UV flood from about 340 nm to 170 nm and an intensity increase of a factor of about 625. The implications of these changes for CCD operation will be discussed in Sections 9 and 10.

It is important to emphasize that SXT X-ray images do not show sensitivity artifacts analogous to what is observed in the visible light images. This is demonstrated from in-orbit data in Section 9.2.3.

A mission-long movie of diffuser images (Acton, 2014) nicely demonstrates the evolution of the diffuser image with time. Up until 13 November 1992 radiation damage accumulated with little evidence of annealing. After that entrance filter failure most of the smaller artifacts gradually disappeared entirely and the heavily irradiated limb features became far less dominant.

**Figure 28:**
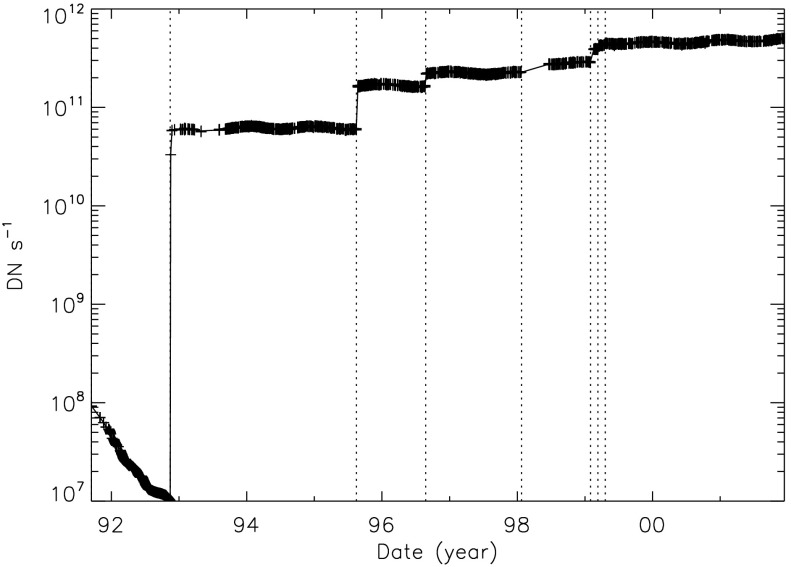
Mission-long plot of diffuser image signal.

Figure 28 presents a mission-long light curve of the total signal in CCD rows 256 to 511 and columns 1 to 1023 of each diffuser image. (The limited CCD area sampled is chosen so that full-resolution partial-frame images, acquired (to prevent saturation) after 24 January 1998 can be included.) Times of known entrance filter failures are indicated by the dotted vertical lines. Note the quasi-exponential decrease in diffuser signal up until November 1992, shared by all optical images. After this, when virtually all of the visible light entering the SXT was coming via the X-ray mirror, such a decrease was never again observed. Interpretation of the mechanisms of optical sensitivity decrease are discussed in Section 6.1.

**Figure 29:**
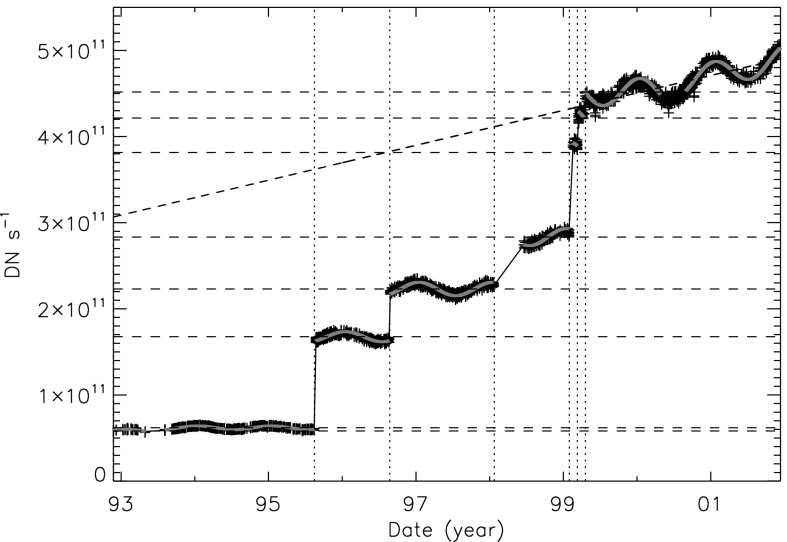
Sinusoidal fits to diffuser signal amplitudes.

Figure 29 displays, on a linear scale, the diffuser signal for the period following 13 November 1992. Between entrance filter failures the diffuser image signal varied sinusoidally in step with apparent solar diameter. In order to study the intensity changes at the steps this variation has been fitted with a sinusoidal expression of the form

1 $$ \mathrm{Sig} = A + Bt + C\sin(Dt + E), $$

where $\mathrm{Sig}$ is the diffuser signal, $A$ is the mean signal, $B$ allows for a slope to the wavetrain, $C$ is the amplitude of the sine wave, $D = 2\pi/\mbox{period}$, $E$ is the phase and $t$ is time in seconds. $A$, $C$, and $E$ were determined by fitting the interval 5 August 1993 to 15 August 1995 with $B$ constrained to 0.0 and $D$ to the period of a solar year (365.2425 days). This fitting determined the phase ($E$) and the ratio of the mean signal to the sinusoidal amplitude, *i.e.*, $C = 0.035A$. For other failure intervals only the mean signal, $A$, was determined by the fitting. Other parameters were held constant to the values determined by the 5 August 1993 to 15 August 1995 fit. The value of $A$ for each time interval is given in the ‘Diffuser signal’ column of Table 7.

**Table 7:** Stray-light amplitude.

Failure date (dd-mmm-yy)	Diffuser signal	Open sectors	Open fraction
13-Nov-92	5.822 × 10^10^	1.03	0.086
1-Aug-93	6.194 × 10^10^	1.10	0.091
16-Aug-95	1.676 × 10^11^	2.97	0.247
24-Aug-96	2.230 × 10^11^	3.95	0.329
24-Jan-98	2.832 × 10^11^	5.02	0.418
30-Jan-99	3.814 × 10^11^	6.75	0.563
12-Mar-99	4.216 × 10^11^	7.47	0.622
20-Apr-99	4.518 × 10^11^	8.00	0.667

**Figure 30:**
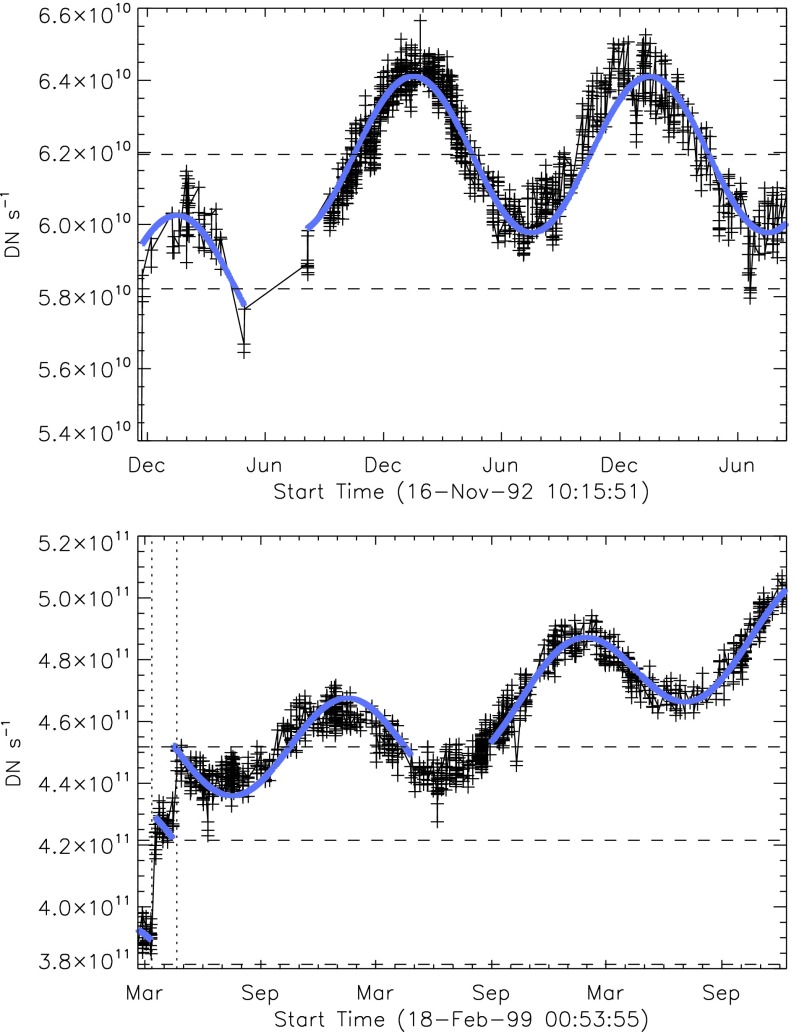
Diffuser signal and sine fits early and late in the *Yohkoh* mission. The horizontal broken lines show the base amplitude of the sine fits.

**Figure 31:**
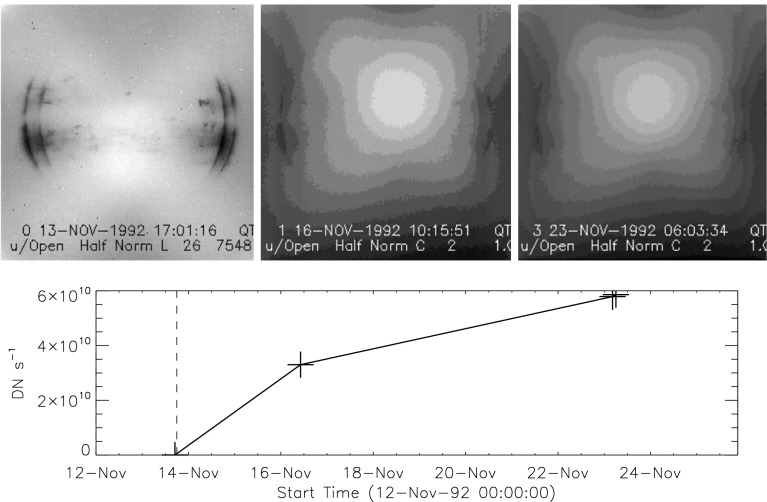
Diffuser signal around time of November 1992 entrance filter failure. The middle and right hand images are decompressed from 8-bit compressed data, accounting for the layered appearance.

Figure 30 illustrates the fitting for early and late in the mission. The two year interval on the right of the upper curve was the reference interval used for determining sinusoidal amplitude and phase. Note that for the fragmentary period prior to 5 August 1993 the signal is lower by about 6 %. This and the single low diffuser signal obtained on 16 November 1992, better illustrated in Figure 31, indicate that the diffuser signal did not attain a stable level until about September 1993. Perhaps the entrance filter did not entirely fail or there may have been flaps of filter material partially intruding into the optical path for awhile. An alternative explanation could be that the enhanced UV flood (Section 9.2.1) from full-spectrum sunlight off of the X-ray mirror took some time to increase the response of the CCD to visible light by annealing out some of the accumulated damage from ionizing radiation. In any case, we assume that the filter-ring open area derived from the diffuser data represents the open area to be used in adjusting the X-ray sensitivity of the SXT.

Figure 29 and the bottom panel of Figure 30 show that following the entrance filter failures of 1999 the stray-light signal began a linear increase of about 4 to 5 % per year that continued until the end of the mission. This increase appeared in every measure of SXT stray light; diffuser, leak monitor, and terminator images. It is not understood what caused this stray-light evolution as it is too smooth, enduring, and steady to be attributed to entrance filter ruptures. Our best guess is that the UV flood after 1999 was sufficiently strong to gradually anneal the ionizing radiation damage to the CCD that caused the evident decrease in sensitivity to visible light. As this phenomenon appears to have no impact on X-ray sensitivity the gradual change evident in Figures 29 and 30 is not taken into account in the SXT X-ray sensitivity adjustment software.

It immediately catches the eye in, *e.g.*, Figure 29 that, except for the failures of 1999, the increases in stray light are very nearly digitally incremental. That is, the steps are in 1, 2, 1, 1, 2, and 1 increments. This discovery helps to better define the changes in entrance filter open area for the purpose of SXT X-ray sensitivity calibration.

Adding up the increments suggests that there are eight totally open $30^{\circ}$ sectors in the entrance filter ring after the final failure on 20 April 1999. Why there were apparently no failures of the remaining four sectors (1, 3, 9, 10) after April 1999 is puzzling. The diffuser signal at that time was $4.52\times10^{11}~\mbox{DN}\,\mbox{s}^{-1}$ indicating a diffuser signal increase per open sector of approximately $5.66\times10^{10}~\mbox{DN}\,\mbox{s}^{-1}$. Assuming the fitted amplitudes listed in Table 7 as genuinely measuring the open filter area we can compute the fractional open area of the entrance filter ring after each failure. The results of this analysis are given in Table 7. The conversion of this information to SXT X-ray sensitivity is presented in Section 7.8.

### Visible-Light Terminator Images

The scientific return of the SXT would have been severely compromised had it not proven possible to acquire X-ray-free stray-light images for an interval of about 13 s at the end of each orbital day. During this brief period the upper atmosphere of the earth absorbs the solar soft X-rays but atmospheric refraction and extinction have not yet significantly affected the visible stray-light pattern, yielding exposures recording only the visible stray light. The images of Figure 21 were acquired in this manner. These so-called terminator images are used to correct for stray light in the X-ray images obtained after 13 November 1992, 18:00 UT.

**Figure 32:**
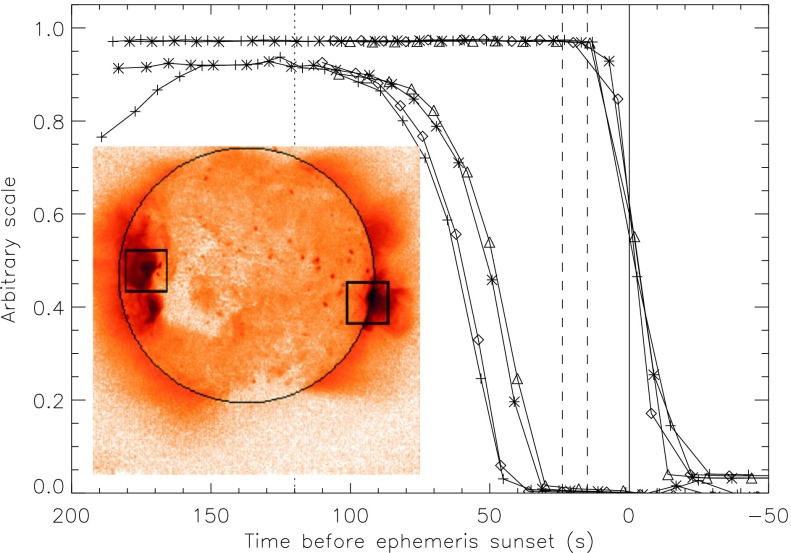
Absorption of X-rays (AlMg filter) and white light at SXT sunset. Inset, Al.1 image obtained at 17:52:30 UT, 19 December 1994, showing east and west observing regions as small squares. Upper curves, stray visible light. Lower curves, X-rays. Cross and diamond, west limb region. Asterisk and triangle, east limb region. Dotted line at 120 s, beginning of X-ray absorption. Broken vertical lines, interval (15 – 24 s) for acquiring Al.1 terminator images. Interval for other analysis filters is 12 – 24 s. Vertical solid line, time of *Yohkoh* ephemeris sunset.

Figure 32 displays results of a special calibration experiment, run in flare mode, which acquired PFIs in X-rays (AlMg filter) and stray visible light from the two regions of interest indicated on the inset image. The two sunsets were observed on 19 December 1994 05:04:24 to 05:09:04 UT (diamonds and triangles) and 18:02:30 to 18:06:38 UT (crosses and asterisks). The scaling of the curves has been adjusted to make the pre-sunset signals coincide. Note that X-ray absorption sets in for the west limb region about 12 s earlier than for the east limb region because the solar west limb leads at orbit sunset. The increase of the X-ray curve designated by crosses on the left is caused by adjustment of the SXT automatic exposure control during the first five exposures of this transit and can be ignored.

**Figure 33:**
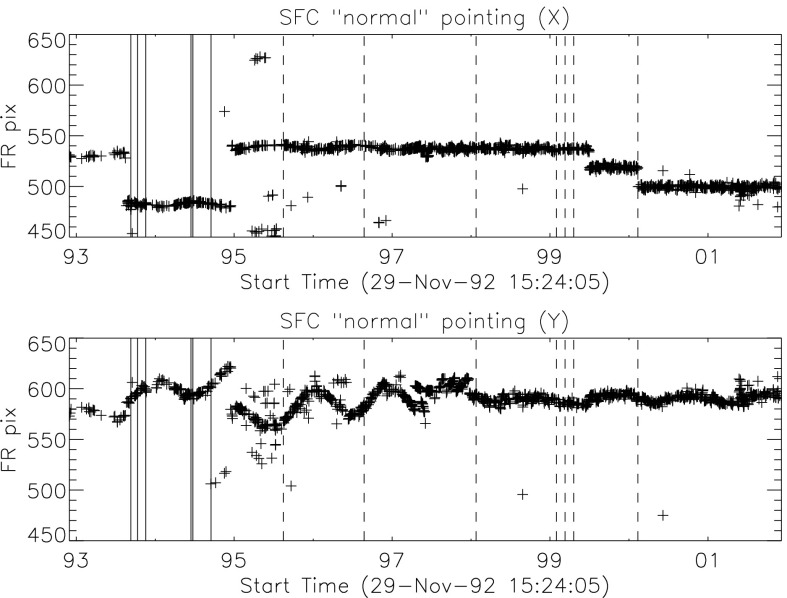
*Yohkoh* pointing history for so-called ‘normal’ pointing. The vertical axes give the full-resolution CCD pixel numbers of solar disk center in E–W (upper panel) and N–S (lower panel). The lower left corner of the CCD is pixel $(0,0)$. Vertical dashed lines denote times of entrance filter failures. Rightmost dashed line is spurious. Vertical solid lines show the times of the SFCs displayed in Figure 21.

*Yohkoh* pointing was quite variable with season. Also, from time to time, the pointing was adjusted to move the heavily irradiated limb regions to different places on the CCD. Beginning in 1998 *Yohkoh* pointing was periodically adjusted by the SXT Chief Observer to ameliorate the seasonal variation in $Y$ (north–south) in order to improve our ability to obtain improved SXT FFI calibration images (SFCs) pointing coverage. Figure 33 illustrates the pointing history throughout the portion of the mission affected by stray visible light.

The SXT is equipped with two six-position filter wheels (see Tables 4 and 6) in front of the focal plane. Each filter wheel has one open position and five filters (Tsuneta *et al.*, 1991). Presence of stray visible light within the telescope dictates that the open–open filter position, the most sensitive X-ray configuration, is unusable. The thinnest SXT analysis filter (Al.1, approximately 6 cm in front of the CCD) transmits about $10^{-6}$ of visible stray light through the thin Al layer. This filter also developed at least three pinholes, which fortuitously lie slightly off of the normal position of the solar image. Finally, the filter wheel assembly itself is not light tight and even for the thicker filters some stray light finds its way through weight relieving holes in the filter wheels and onto the CCD. The pattern and intensity of this stray light varies from filter to filter, with season (solar diameter), and with *Yohkoh* pointing. The stray-light intensity increases stepwise with each entrance filter failure. Figure 21 illustrates the pattern and relative intensity of the stray-light pattern for all five of the SXT analysis filters.

As is clearly evident in Figure 21 the stray-light patterns for the totally opaque filters AlMg, Al12, and Be are quite similar. The Mg3 stray-light patterns shown in Figures 21(D) and (E) exhibit what appear to be wrinkles and is not fully understood. This is largely irrelevant as this analysis filter was seldom used.

Figure 21(J) illustrates the relative total intensity in the terminator images on a logarithmic scale. The stray light for AlMg (column 1) and Al12 (column 3) are low because these shiny metals reflect most of the stray visible light which falls on them. The Be filter (column 2) has a diffusive surface so that much more of the stray light is scattered internal to the filter wheel assembly where it can find its way to the CCD. Most of the stray light of Al.1 (columns 5 and 6) penetrates the 126.5 nm of Al or comes through the pinholes although the characteristic stray pattern of the opaque filters, *i.e.*, coming at the sides, is also weakly present for Al.1.

**Figure 34:**
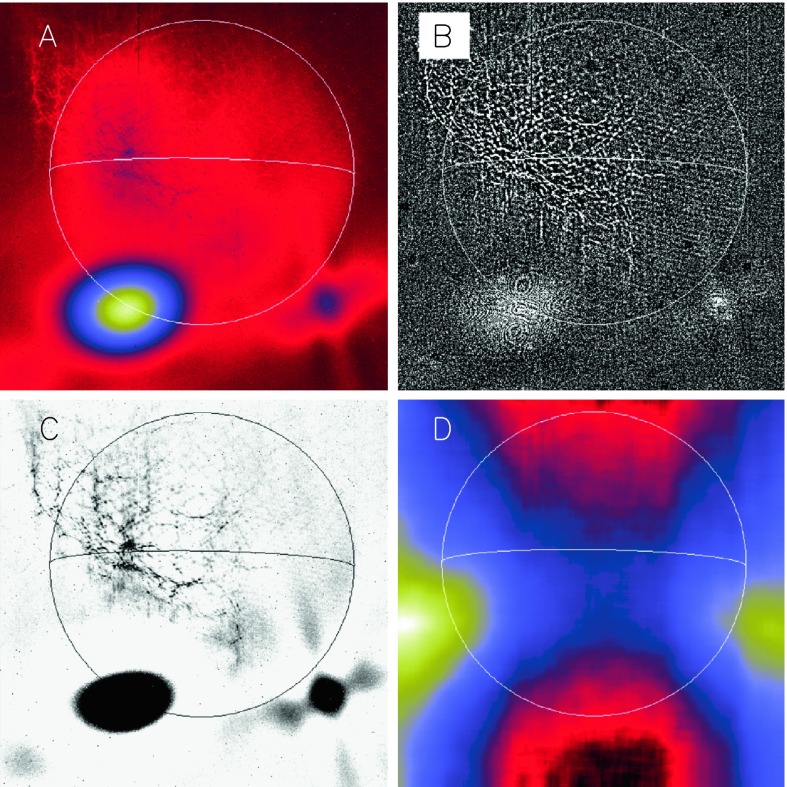
(A) Al.1 terminator image of 23 January 1993, 05:33 UT. (B) difference image illustrating the NE to SW grill pattern on the solar disk. (C) difference image showing the semi-random stray-light network. (D) AlMg terminator of 25 February 1993, 07:52 UT, shown for comparison.

Figure 34 illustrates the complex, low-level, artifacts in Al.1 images. For this reason AlMg became our primary FFI filter after 1992. Although special care must be exercised in interpreting Al.1 images of faint coronal regions the stray-light problem is much less important for the vastly brighter flare and active region features. The Al.1 stray-light network varies in detail as well as in position and intensity. It is at its brightest right after CCD bakeout. We believe that this complex pattern is caused by doubly reflected stray light that enters through the Al.1 filter pinhole(s), reflects off the face of the CCD onto the back of the Al.1 filter, and thence goes back onto the CCD where it is detected. This interpretation can account for the varying pattern as the thin Al membrane distorts under direct solar heating by sunlight focused on it by the X-ray mirror. Although this particular stray-light feature has been extensively studied (see the SXT Observation Notes section of the YLA) it has not proven possible to derive a general correction for this (faint) stray-light pattern.

### Chronology of Entrance Filter Failures

As a result of failures of the thin-film entrance filters, stray visible light began to enter the telescope on 27 October 1992. On 13 November 1992 an inner layer of the duplex entrance filter system failed and the inside of the telescope was flooded with the full solar spectrum. Aspect sensor images could no longer be acquired as even the shortest exposures were totally saturated.

**Figure 35:**
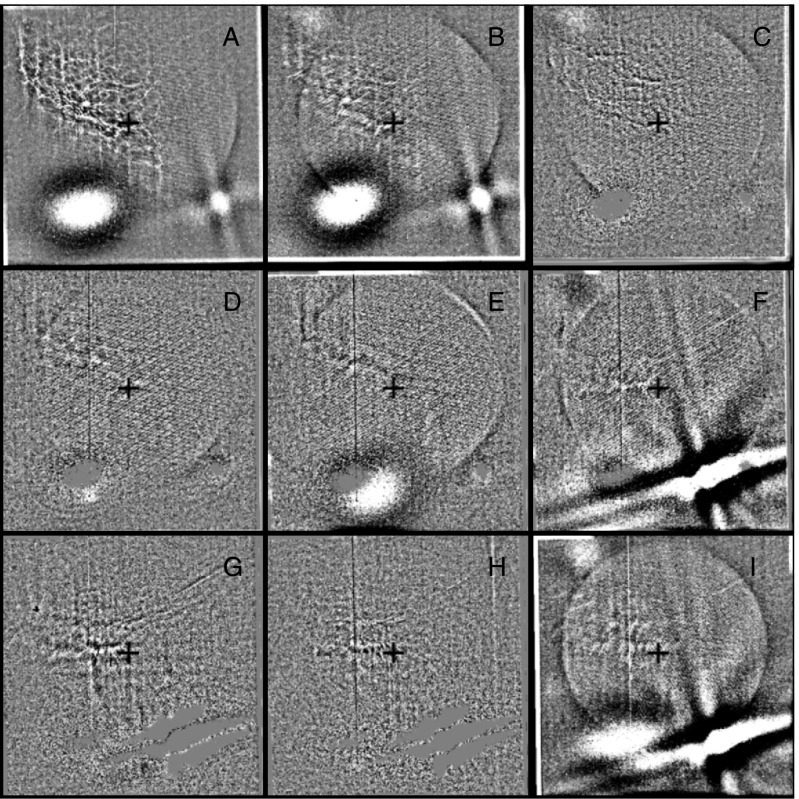
Change in Al.1 terminator at each entrance filter failure. The image times are given in Table 8. The preparation and interpretation of this figure is discussed in the text.

As discussed in Section 7.1.1 and shown in Figure 15 the stray-light image coming off the X-ray mirror is fairly well focused in the direction orthogonal to the radial direction to the failed sector. Thus, after-minus-before difference images may show a well-defined limb in this orthogonal dimension (*i.e.*, $90^{\circ}$ from the failed sector) which identifies (with an $180^{\circ}$ ambiguity) which sector has failed. Figure 35 was prepared to help identify which entrance filter sectors failed at each event. The terminator images comprising this figure have been co-aligned. The black cross indicates the location of solar disk center. All panels of Figure 35 have been prepared by subtracting a 21-pixel boxcar smoothed image from the terminator image to bring out the fine structure. Figures 35(A) and (B) were obtained shortly after the November 1992 and August 1995 failures. In Figure 35(A) a well-defined limb appears at the 04:00 and 10:00 o’clock positions, indicating failure of sector 6 or 12. As we know that the outer filter of sector 6 failed on 27 October 1992 we assume that the inner filter of sector 6 failed on 13 November 1992. Figure 35(B) shows an additional limb feature at about 01:30 and 07:30 o’clock, indicating failure of sector 3 or 9. Figure 35(C) is the difference image of the image in Figure 35(B) minus a similar image taken shortly before the failure. The new limb features are evident. Figures 35(D) – (H) are similar after-minus-before difference images for subsequent failures, not all of which show clear limb features, perhaps because of inadequate statistics. Figure 35(I), created in the same way as Figures 35(A) and (B), is the last good Al.1 terminator image of the mission, obtained on 22 November 2001, 15:18 UT. Except for angles around 12 o’clock a nearly complete limb feature is displayed. The limb gap around 12 o’clock is consistent with sectors 3 and 9 not having failed. Our conclusions of which sectors failed and when are summarized in Table 8 where the letters in column two correspond to the panels of Figure 35.

**Table 8:** SXT entrance filter events.

Event	Figure 35 panel	Date, time (UT) (dd mmm yy hh:mm)	Failure	Change
0		27 Oct 1992 05:59	Outer sector 6	First failure
1		27 Oct 1992 07:18	Outer sector 7	Second failure
2	A	13 Nov 1992 18:00	Inner sector 7	PH^1^ in the SE
3	B, C	16 Aug 1995 08:04	Sectors 5 and 11	New PH in the NE
4	D	24 Aug 1996 07:00	Sector 2	
5	E	24 Jan 1998 00:00	Sector 8	Expanded PH in the SE
6	F	30 Jan 1999 23:17	Sectors 6 (inner) and 12	PH in the SW
7	G	12 Mar 1999 02:00	Part of sector 4	Expanded PH in the SE
8	H	20 Apr 1999 19:02	Rest of sector 4	Now 8 open sectors
9	I	11 Feb 2000 13:00		*X*-shift enhances SE PH
10		14 Dec 2001 21:12		End of mission

^1^Pinhole.

### Details of Stray-Light Changes at the Steps

As the SXT entrance filters failed the stray light increased stepwise within the instrument. In addition, on at least two occasions, a new pinhole appeared in the Al.1 analysis filter at or near the same time as the stray-light step. This is presumably due to the increased thermal shock to the Al.1 filter. We were extremely fortunate that there were no pinholes on the image of the solar disk in normal *Yohkoh* pointing.

**Figure 36:**
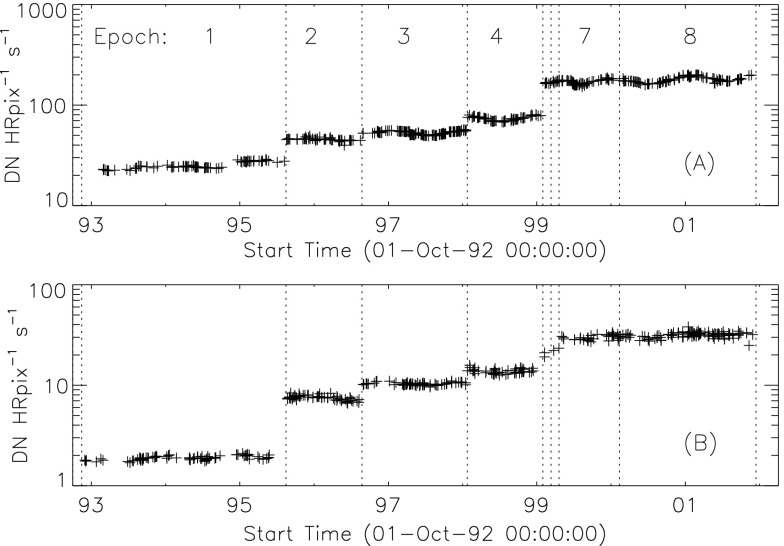
Stray-light signal in terminator (SFC) images: (A) Al.1 filter, (B) AlMg filter.

**Figure 37:**
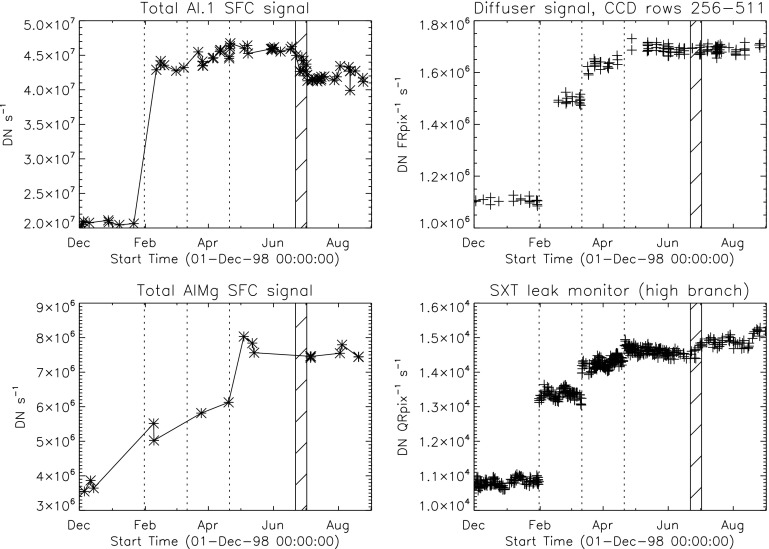
Comparison of stray-light signals from all four monitoring systems in 1999. The cross-hatched interval denotes when *Yohkoh* exhibited a 10 FR pixel ($25''$) drift to the north, moving the solar image farther away from the Al.1 pinholes.

There are four means of monitoring stray visible light in SXT. These are the Al.1, and AlMg terminator images (SXT FFI calibration images; SFCs), the leak monitor images (see Figure 22), and weekly exposures obtained using the opal glass diffuser. The amplitude and evolution of stray light as recorded by the four different techniques are illustrated in Figures 36 and 37. The latter figure shows details of the differences in the stray-light changes for the interesting interval in 1999 spanning leak epochs 4 to 7. Vertical lines in all these figures denote the time of recorded stray-light changes.

### Diagnosis of Open Filter Area

Table 8 presents conclusions of which filters opened at each stray-light step. Clearly, there are details not grasped by this difficult analysis. For example, it is very hard to believe that both front and rear filters in two sectors $180^{\circ}$ from each other (5 and 11 or 6 and 12) would all open simultaneously. Furthermore, examination of Figure 29 shows that filter sectors may not always fail entirely so, lacking better information, a direct measure of stray-light levels from analysis of the diffuser signal will be used as a proxy for entrance filter open area.

Assumptions are (1) that the intensity of the diffuser signal after 13 November 1992 is a faithful measure of the fraction of the entrance filter ring for which both outer and inner filters are open, (2) sector 6 of the outer filter ring opened on 27 October 1992 but sector 6 of the inner ring did not fail until 30 January 1999 and (3) as of 20 April 1999 eight and only eight sectors had completely opened. The second assumption results from the analysis of Figure 35 which seems to indicate that inner sector 6 failed on 30 January 1999. Assumption (3) is buttressed by the semi-digital nature (Table 7) of the failure steps, adding up to eight. We have no way of knowing if additional outer-ring-only failures occurred after the first failures in October 1992.

Given these assumptions, a model treating each (outer and inner) filter ring separately, and the diffuser intensities given in Table 7, it is straightforward to compute the increases in SXT spectral sensitivity at each failure step.

**Figure 38:**
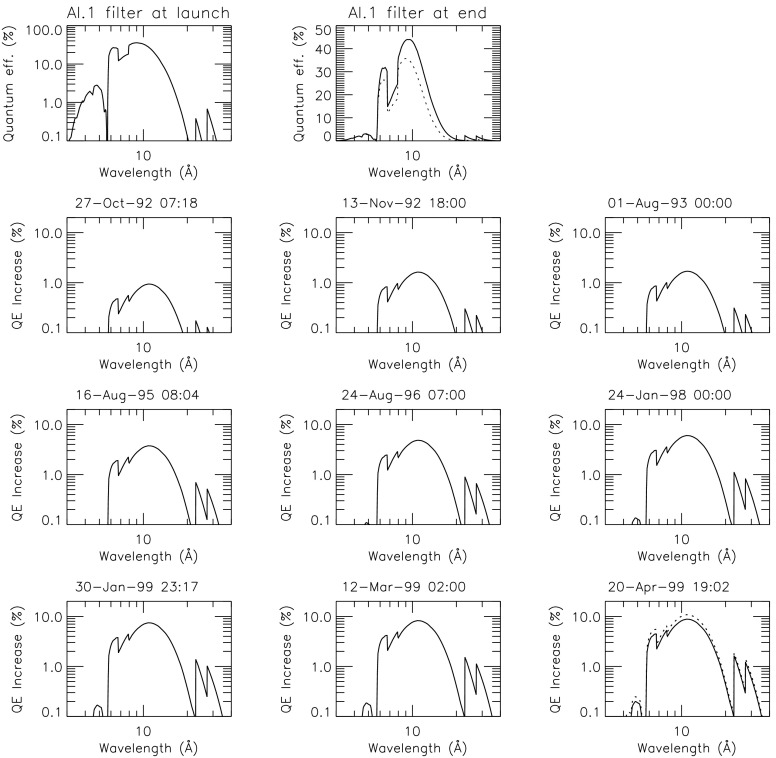
Upper left: quantum efficiency (QE) of the SXT with the thin aluminum (Al.1) analysis filter at launch. Upper middle: Al.1 QE at end of mission on a linear scale. Dotted line shows launch QE. Other panels: step by step absolute increase in SXT spectral QE due to entrance filter failures for the Al.1 analysis filter. The total QE at any time would be the sum of the upper left curve plus the appropriate failure date curve. The dotted curve in the lower right panel shows the increase for the hypothetical case where all outer filters are open.

All of the SXT programs for deriving physical parameters from instrumental units treat the entrance filters as a single ring, *i.e.*, combining outer and inner. In order to leave these programs unchanged an effective single ring open area has been derived which yields an SXT spectral sensitivity equivalent to the full calculation with two filter rings. This permits incorporating the new calibration results by the simple updating of a single *Yohkoh* data base. A comparison of the old and revised open entrance filter fraction is given in Table 9. The increase in spectral sensitivity for the Al.1 analysis filter at each of the nine failures is illustrated in Figure 38. The increases in sensitivity for all of the thicker filters will be less than for Al.1.

**Table 9:** Entrance filter open fraction.

Event time (UT) (dd-mmm-yy hh:mm)	Old open fraction	New open fraction
30-Aug-91 01:30	0.000	0.000
27-Oct-92 07:18	–	0.071
13-Nov-92 18:00	0.083	0.123
1-Aug-93 00:00	–	0.128
16-Aug-95 08:04	0.167	0.284
24-Aug-96 07:00	0.250	0.366
24-Jan-98 00:00	0.333	0.455
30-Jan-99 23:17	0.417	0.563
12-Mar-99 02:00	0.500	0.622
20-Apr-99 19:02	0.583	0.666

Plasma temperature diagnostics using the filter ratio method is only an approximation at best because one ratio can only return one temperature whereas the Sun will usually have a range of temperatures along any given line of sight. With multiple filters the ability to model multi-thermal plasmas is improved. In any case, the emission-measure-weighted average temperature returned by the two-filter method is often useful (Acton, Weston, and Bruner, 1999). We believe that, with the filter-failure adjustments to SXT quantum efficiency (*i.e.*, spectral sensitivity) discussed in this section, the usefulness of SXT data for temperature studies is, for bright X-ray features, essentially as reliable as if the entrance filters had not failed.

## SXT Stray Light Correction

**Figure 39:**
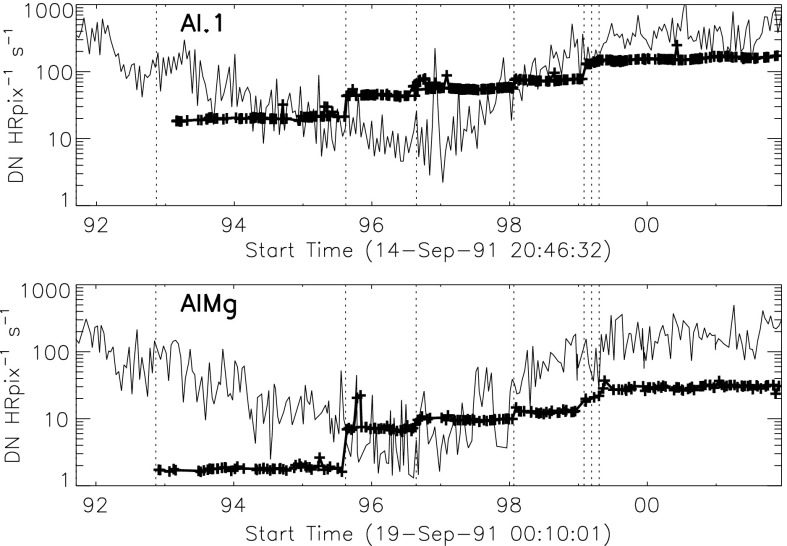
Comparison of mean X-ray (thin lines) and stray-light (heavy lines) signals for the two thinnest SXT analysis filters. Dotted lines indicate times of filter failures. Vertical excursions of stray-light points are from SXT offpoints.

The comparative full-image signal levels of X-rays and visible stray light are illustrated in Figure 39. For this comparison the on-disk signals of each half resolution (HR) image were normalized to $\mbox{DN}\,\mbox{HRpix}^{-1}\,\mbox{s}^{-1}$. For convenience in plotting the X-ray signals from the selected SXT science composite (SSC) images are about 14 days apart. All of the stray-light Al.1 terminator (SFC) images are included. For the Al.1 SFCs the disk area sampled does not include the pinholes to the south. These pinholes dominate the total stray-light signal but contribute little to on-disk stray light.

It is clear from Figure 39 that, for disk-averaged signals, the stray-light corrections are significant, especially for Al.1. During solar minimum the stray visible light exceeds the X-rays. However, this is not the whole story because the SXT X-ray images have very high contrast. For example, a typical active region X-ray signal exceeds $20\mbox{,}000~\mbox{DN}\,\mbox{HRpix}^{-1}\,\mbox{s}^{-1}$, quiet coronal loops are of the order of $100~\mbox{DN}\,\mbox{HRpix}^{-1}\,\mbox{s}^{-1}$ while coronal holes have signal levels of only $10~\mbox{DN}\,\mbox{HRpix}^{-1}\,\mbox{s}^{-1}$. It is indeed fortunate that it has proven possible, in most cases, to correct the X-ray images for visible stray light by subtracting a properly chosen and adjusted SFC from the X-ray exposures. This is more successful for the AlMg images than for the Al.1 exposures because of the more complicated, time varying, nature of the stray-light patterns through the Al.1 analysis filter. Thus, extreme care must be taken in interpretation of Al.1 images of the quiet corona and, especially, coronal holes obtained after 13 November 1992.

The following sections describe our efforts to obtain the best possible stray-light correction throughout the SXT mission.

### Terminator Images

Terminator images were collected at every opportunity to get good coverage over a range of spacecraft pointings and solar diameter. The conditions for acquiring these images, called TermSFC, are described in Section 7.5. Periodically over the course of the *Yohkoh* mission failures of the front entrance filters occurred as detailed in Table 8. Over each of the epochs a set of terminator images were collected with as complete a coverage of pointing and solar diameter as possible. Examples of terminator SFCs collected during leak epoch one are shown in Figure 21. For a variety of operational reasons the coverage is necessarily incomplete. Special observations such as *Yohkoh* offpoints are treated on a case-by-case basis with special terminator images acquired at the time.

The correction is applied by selecting the terminator image for a given leak epoch which best matches the X-ray image in *Yohkoh* pointing and solar diameter. For each X-ray image the SFC is exposure-normalized and subtracted from the X-ray exposure to remove the white light contamination. For Al.1 there is an additional step of subtracting the grill pattern (see Figure 34) from the on-disk portion of the image. This correction does not alter the total intensity as the net intensity of the grill correction pattern equals 0.

### Synthetic Terminator Images

While the correction of the white light leak is adequate in many cases, there are instances where the nearest terminator image is not sufficiently close in pointing and solar radius and the correction is poor. The SXT team (Shirts *et al.*, 2003) derived a multi-dimensional algorithm to interpolate, pixel by pixel, available terminator images to provide parameters for creation of synthetic terminator images for a finer grid of spacecraft pointings and solar radii. These are called SynSFC.

The generation of parameters for synthetic terminator images is done on an epoch by epoch basis corresponding to successive failures of the front entrance filters. Table 8 details the epochs. Throughout each epoch, terminator images are obtained as often as possible.

#### Application of the Interpolation Algorithm

**Figure 40:**
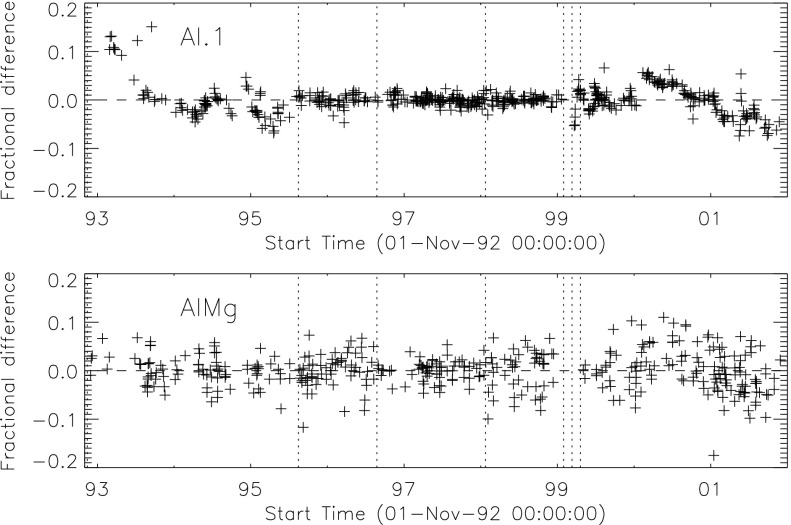
Fractional difference, $(\mathrm{SynSFC}-\mathrm{TermSFC})/\mathrm{SynSFC}$, of the interpolation algorithm produced by generating interpolated images at the same pointing, *etc*., as an actual terminator image for the two thin SXT analysis filters. The vertical lines indicate the times of entrance filter failures.

We have tested the interpolated terminator images by generating a synthetic terminator at the same coordinates and date as the actual terminator. The results are shown in Figure 40 as the ratio of the mean difference between the synthetic terminator and the actual terminator divided by the brightness of the synthetic terminator. This analysis demonstrates that the synthetic method is sound although better in some epochs than in others. The scatter in the figure is indicative of the natural variation of the terminator images. It is much larger for AlMg due to the statistical variation of the fainter AlMg leak images. This variation is in the form of an overall scale factor and does not unduly affect the morphology of the leak pattern. This residual variation is removed by using a second order leak correction (*sxt_deleak.pro*) which adjusts the overall scale of the leak or synthetic leak image to a particular X-ray data image until the lower left corner of the corrected X-ray image is consistent with the expected scattered X-rays from the solar disk.

### Choice of Stray-Light Correction Image

Neither terminator nor synthetic SFCs always provide the optimum correction for stray light, especially for the Al.1 analysis filter. A procedure is required to choose which type of SFC to use for correcting a given SXT image.

**Figure 41:**
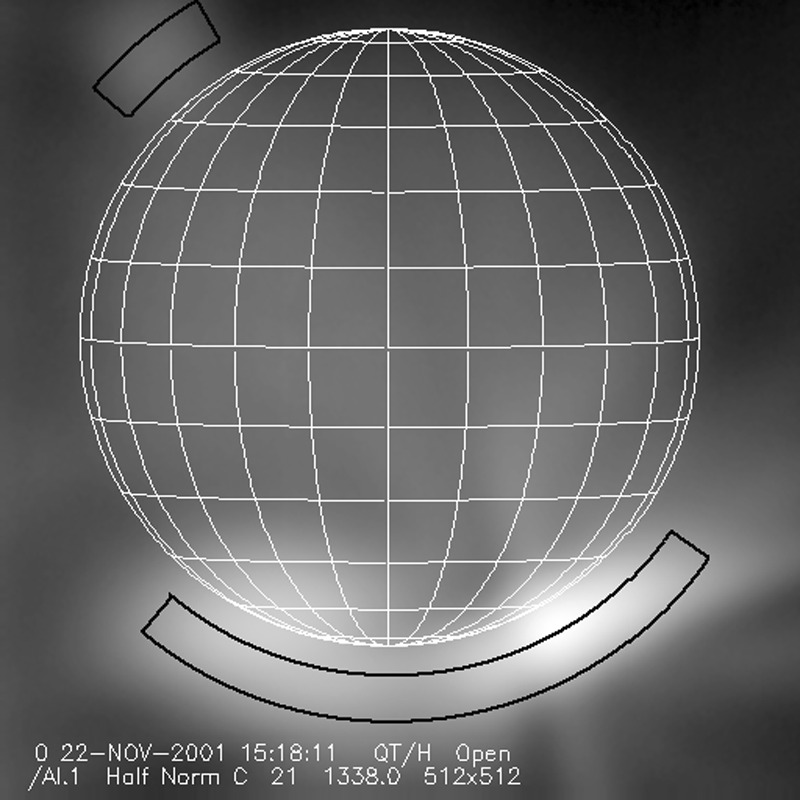
Image areas sampled for stray-light correction analysis. The areas are fixed with respect to the solar image, *i.e.*, not as absolute CCD coordinates.

The signal in off-disk areas most affected by stray light, namely $1.1\,\mbox{--}\,1.25~R_{\odot}$ for azimuths $125^{\circ}\,\mbox{--}\,220^{\circ}$ and $310^{\circ}\,\mbox{--}\,330^{\circ}$, for all Al.1 level-2 FFIs have been prepared for comparison with AlMg FFIs taken near in time. For images taken close together in time, the best-corrected Al.1 image is that for which the signal in the sample areas is closest to twice the signal (under most conditions the Al.1 FFI images have an integrated signal very close to two times the equivalent AlMg image with the same exposure) in the same area for the AlMg image. The areas sampled for this comparison are shown in Figure 41.

Making this comparison for all Al.1 level-2s corrected with SynSFC and the same Al.1 FFIs corrected with TermSFC shows where, on average, SynSFC or TermSFC are better. The chosen SFC type boundaries are listed in Table 10. The SXT analysis software employs this choice for both the Al.1 and the AlMg observations.

**Table 10:** Choice of SFC.

Start time (UT) (dd-mmm-yy hh:mm)	SFC type
*Yohkoh* launch	No SFC required
13-Nov-92 18:00	TermSFC
24-Jul-93 00:00	SynSFC
16-Sep-94 00:00	TermSFC
24-Aug-96 07:00	SynSFC
30-Jan-99 23:17	TermSFC
20-Apr-99 19:02	SynSFC

## The SXT CCD Detector

The CCD camera for the SXT utilizes a $1024\times1024~\mbox{pixel}$ virtual phase (also called uniphase) CCD with 18.3 μm pixels manufactured especially for SXT by Texas Instruments (TI) at their Miho, Japan, facility. The principle of operation of this type of CCD is described by Janesick (2001). A TI virtual phase CCD was also flown on the ESA *Giotto* mission. The performance of that CCD after seven years in interplanetary space has been discussed by Kramm, Thomas, and Keller (1993).

**Figure 42:**
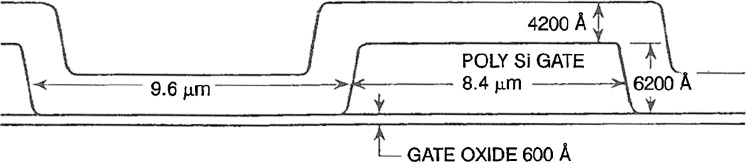
Schematic sketch of the structure of the Texas Instrument uniphase CCD used on the SXT. Not to scale, thicknesses in this figure are approximate. The actual thicknesses of the layers for the SXT flight CCD are: $\mathrm{SiO}_{2}$ overcoat, 487 nm; polysilicon gate structure, 770 nm; $\mathrm{SiO}_{2}$ gate oxide, 63 nm.

The structures overlying the sensitive volume of the CCD are illustrated in Figure 42. The selling points of this CCD for SXT were its relatively good soft X-ray sensitivity in the half of each pixel not covered by the polysilicon gate (Figure 46). Shin and Sakurai (2014) have discussed the subtle effect that the CCD pixel structure has on the point spread function of the SXT. Note that, in use, the sensitivity of the device longward of 100 nm was eliminated by metallic analysis filters (Table 6).

### Amplifier Gain of the SXT CCD Camera

The 12 bit data numbers (DN) from the camera analog-to-digital converter (ADC) were normally compressed to eight bits for downlink through a look-up algorithm described in detail by Tsuneta *et al.* (1991). The algorithm is linear with ADC output up to $\mathrm{DN}=64$ and goes approximately as the square root of the ADC number beyond that. $\mathrm{DN}=255$ decompresses to 4095. This corresponds to approximately 960 photons of 8 Å wavelength.

**Figure 43:**
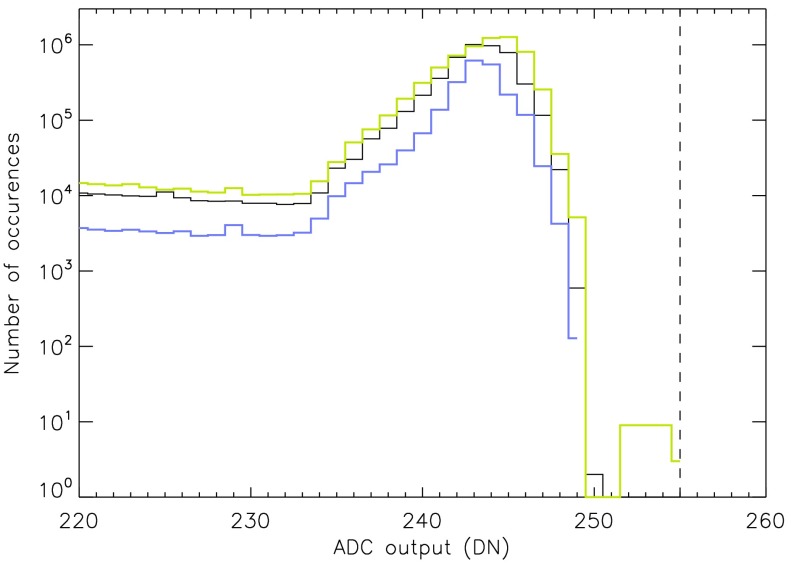
Histograms of DN values (after compression) of saturated full-resolution SXT pixels. The data have been divided into three equal mission time intervals indicated by the color of the histograms. Black: first third (460 images), blue: second third (102 images), yellow: final third (445 images). The broken vertical line denotes the maximum output (compressed) of the ADC at 255 DN.

If the CCD camera gain changed during the *Yokoh* mission this would result in an over or under estimation of the X-ray flux. The electronic gain of the SXT CCD camera was set to approximately 100 electrons per DN so that full-well capacity (about $2.5\times10^{5}~\mbox{electrons}$) of the CCD would reach saturation (in FR mode) before the 12-bit ADC saturated. In $2\times2$ and $4\times4$ summed modes the ADC saturates first. The gain was set pre-launch to provide a full-well compressed signal for an average CCD pixel of about 245 DN. Figure 43 presents histograms of the high-end signals of the pixels in 789 long-exposure FR images taken throughout the mission. The peak near $\mathrm{DN}=244$ demonstrates that the CCD camera gain was set properly. The width of the peak shows the pixel-to-pixel variation in full-well capacity while the few cases with DN values above 250 are from anomalous pixels. The histograms for the early, mid, and late phases of the mission prove that the gain was stable throughout the mission. A shift of one DN at 244 DN would represent about a 1 % change in gain.

**Figure 44:**
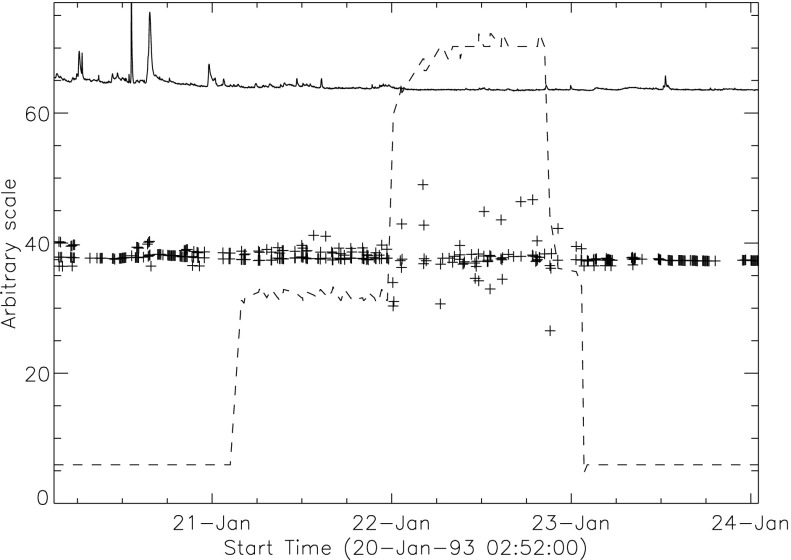
Demonstration that the CCD on-chip amplifier gain is not temperature dependent. Broken line: CCD temperature (−20 °C, 0 °C, 20 °C). Solid curve: GOES low channel signal on a linear scale. Crosses, mean signal (unsaturated pixels, $\mbox{DN}\,\mbox{s}^{-1}$) in AlMg full frame images. Increased scatter during +20 °C interval is caused by enhanced dark signal correction.

An independent check of the temperature stability of the CCD on-chip amplifier is given in Figure 44, which shows the mean quiet corona signal (saturated pixels eliminated) taken with the AlMg filter through a CCD bakeout in January 1993. The increased scatter due to an enhanced dark signal when the CCD was warmed to 20 °C does not mask the evidence for X-ray signal stability.

The preferred means of measuring CCD camera gain is by the photon transfer method (Janesick, 2001) whereby an extrapolation of a log–log plot of signal variance *versus* signal, for a range of exposures, yields the gain. An optical diffuser (Section 7.4) was incorporated into the forward filter wheel of the SXT in order to provide CCD illumination for this calibration. Unfortunately, radiation damage (Section 9.2) increased fixed pattern noise and so compromised the visible-light response of the CCD that photon transfer experiments were difficult. Photon transfer experiments were executed in October 1992, April 1993, and twice in April 1996 (LaBonte, 1996). All of these experiments were consistent with a CCD camera gain of approximately 90 electrons $\mbox{DN}^{-1}$. The 1996 experiments by Barry LaBonte had a one sigma statistical error of a few percent.

It was not until June 2014 that the default CCD gain was changed from 100 to 90 electrons $\mbox{DN}^{-1}$ in SXT analysis software. This change results in a 10 % decrease in X-ray fluxes and emission measures derived from SXT observations.

### CCD Damage from Ionizing Radiation

#### UV Flood

During CCD testing (only a bit more than one year before launch) Jim Janesick of JPL discovered that these devices suffered damage from ionizing radiation (X-rays in particular), which, when sufficiently severe, caused increased dark current, flat-band shift and, ultimately, unpinning of the device. Janesick interpreted this phenomenon and determined that increasing the camera drive voltage (to $-6~\mbox{V}$ beyond the inversion point) plus exposure of the CCD to UV photons could, to some degree, ameliorate the problem (Acton *et al.*, 1991; Janesick, 2001). The flight results reported in Section 9.2.2 demonstrate that, with sufficient overexposure, the problem persisted but the UV flood appears to have corrected it.

**Figure 45:**
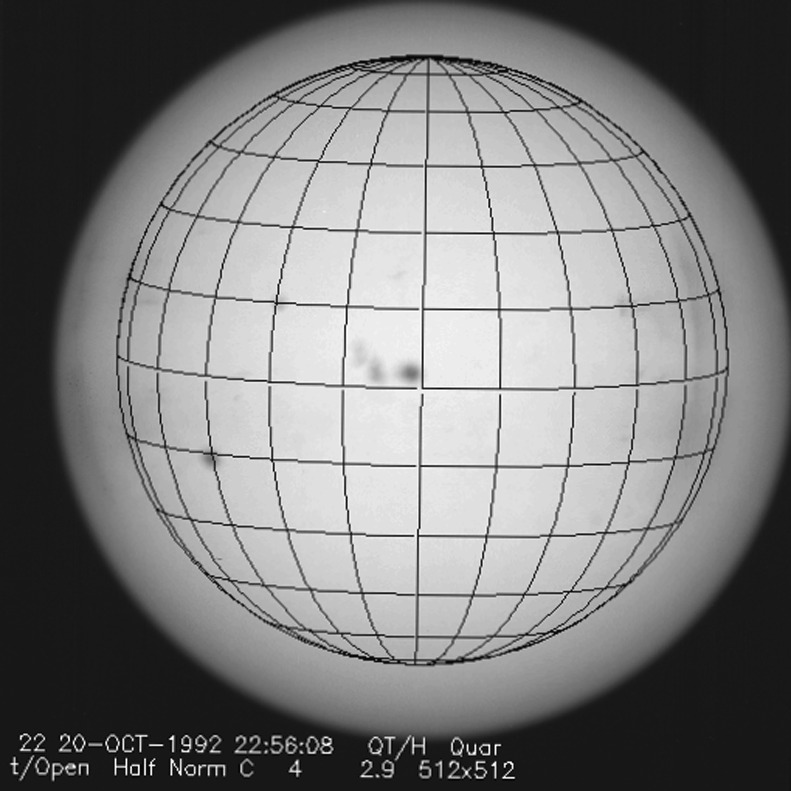
Picture using quartz defocus lens. Overlay shows position and size of an in-focus solar image.

As a result of Janesick’s discovery a slightly negative quartz lens was incorporated in the forward SXT filter wheel to illuminate the CCD with an oversized ($1.2~R_{\odot}$) out-of-focus 330 – 450 nm image of the Sun from light passing through the aspect sensor optics (Figure 45). This blue-light flood, although lesser in photon energy than ideal, was intended to help neutralize the damage caused by the X-rays. The first 3 – 5 min of each daylight pass of *Yohkoh* was devoted to a blue-light flood of the CCD. As the transmission of the aspect sensor optics diminished this blue-light flood became correspondingly less intense. While all optical images showed clear evidence of CCD radiation damage (see Section 7) by November 1992 there was as yet no corresponding evidence of enhanced dark signal in CCD dark frames.

After the 1992 November 13 entrance filter failure the blue-light flood became a much more effective UV flood, at least for the half of each pixel not covered by the polysilicon gate structure. As illustrated in Figure 47 intensity through the quartz lens on the CCD became nearly $10^{11}$ times higher than before. This was determined by modeling the CCD response from 0.1 to 1000 nm using absorption coefficients from Drummond (1936), Green and Keevers (1995), Henke, Gullikson, and Davis (1993), Kitamura, Pilon, and Jonasz (2007), Narukage *et al.* (2011), Palik (1985), Philipp (1985), Tan, Lemon, and French (2003). It is probable that, in the absence of the entrance filter failures, accumulated CCD damage from ionizing radiation would have, in time, rendered the most heavily irradiated portions of the CCD unusable.

Roughly 1 % of the soft X-rays incident on the CCD were absorbed in the $\mathrm{SiO}_{2}$ gate oxide layer, producing electron-hole pairs and the resulting radiation damage. As detailed by Acton *et al.* (1991) a certain fraction of these positively charged holes migrate to the oxide-silicon interface, upsetting the voltage potential of the pixel. Laboratory experiments demonstrated that UV radiation more energetic than the silicon valence band (292 nm), which is absorbed in the bulk silicon very near the oxide-silicon interface (UV flood), results in photo-emission of electrons from the bulk silicon into the oxide – neutralizing the positive charge of the holes produced in the oxide by ionizing radiation. Radiation shortward of the conduction band but longward of the valence band, which we termed a blue-light flood, is believed to be less effective but better than nothing.

**Figure 46:**
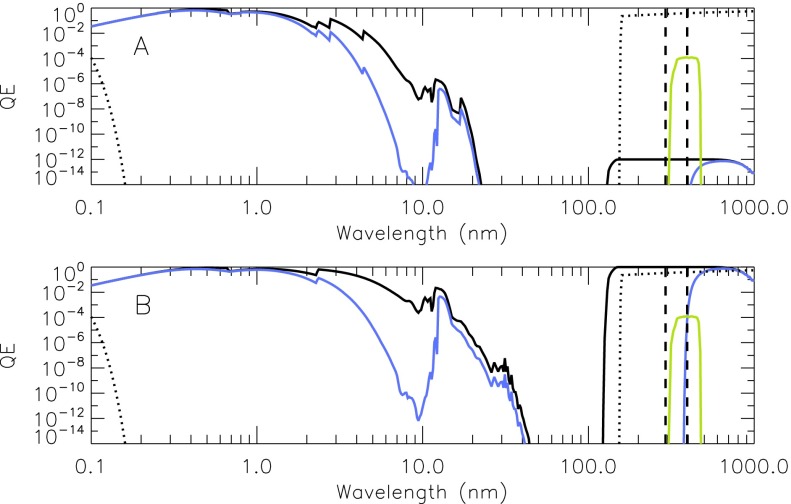
Spectral quantum efficiency (QE) of the CCD plus optics with (A) and without (B) an entrance filter. The blue curve denotes portions of the CCD pixel covered by the polysilicon gate. The black curve is the part of the pixel with no overlying gate structure. The dotted curve is the transmission of the quartz defocus lens (4 mm thick). The yellow curve shows the transmission bandpass of the SXT aspect sensor optics. The vertical broken lines show the positions of the valence (left) and conduction (right) bands of the bulk silicon beneath the insulating oxide layer.

**Figure 47:**
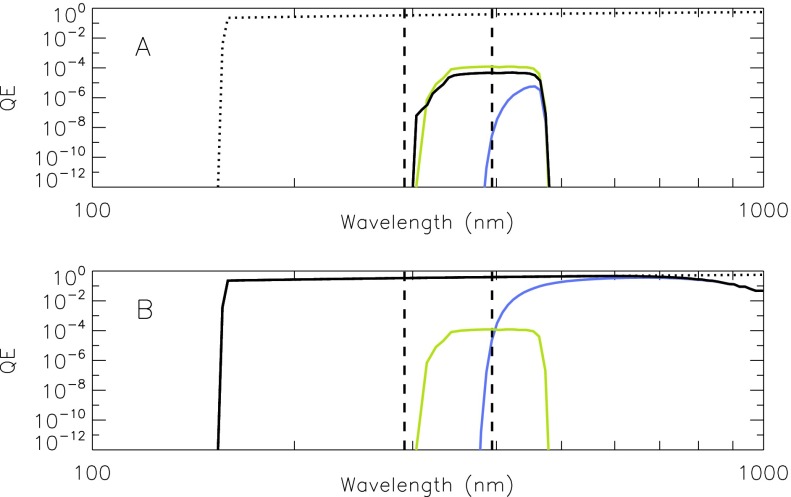
Detail of the spectral response of the optical system (CCD plus optics) in the ultraviolet with (A) and without (B) the aspect sensor optics in place and with the quartz defocus lens included in the computation. Description of curves are the same as for Figure 46. The dotted line in A, transmission of the quartz lens, is shown for reference only as this optical element plays no role in system transmission when the aspect sensor assembly is included.

Figures 46 and 47 clearly demonstrate the response differences of the two halves of each CCD pixel. Figure 46 does not include the effect of SXT aspect optics and the quartz defocus lens, whereas Figure 47(A) does include both and Figure 47(B) the quartz lens alone. Figure 46 shows that, in the visible range beyond 400 nm, the two halves of the CCD are similar. However, the blue-light and UV floods, especially after 13 November 1992, should be much more effective for the X-ray sensitive half of the CCD than for the gate half. Perhaps this accounts for the radiation damage features in the optical images persisting at the 1 % level throughout the mission.

#### CCD Overexposure Glitches

**Figure 48:**
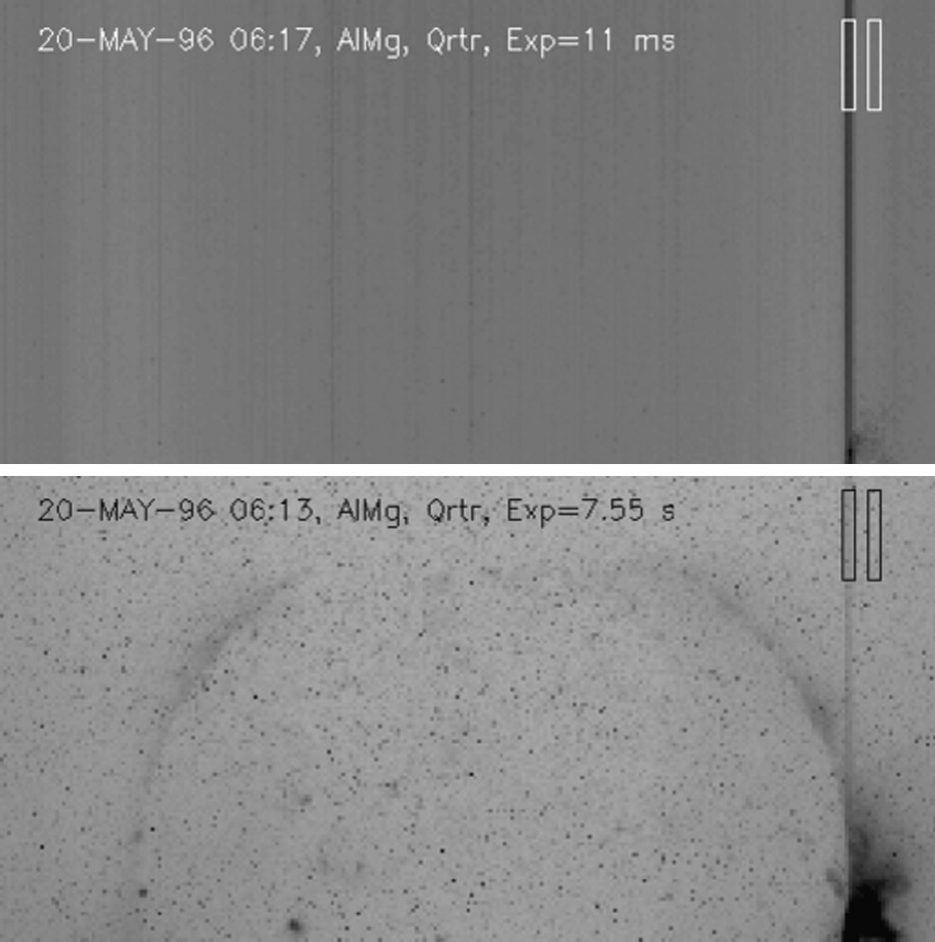
Long and short exposure QR images illustrating the large glitch of May 1996. The boxes show the sampling locations for the light curves of the following figure.

Severe X-ray overexposure of the CCD creates a temporary area of ionization damage that releases charge as the CCD is read out, producing a vertical trail of signal enhancement, a ‘glitch’, above and below the overexposed region in CCD images. This effect is caused by the partial unpinning of the implant portion of the virtual phase CCD (Acton *et al.*, 1991) by ionization of the implanted boron atoms. A strong example is illustrated in Figure 48. Note that the intensity of the glitch, while it appears stronger in the short exposure, is actually almost independent of exposure duration. This is because the signal enhancement is caused by charge pickup from the damaged area as the image is clocked out. There will be a small dependence upon exposure time because additional X-ray exposure has a tendency to ‘charge up’ the traps.

**Figure 49:**
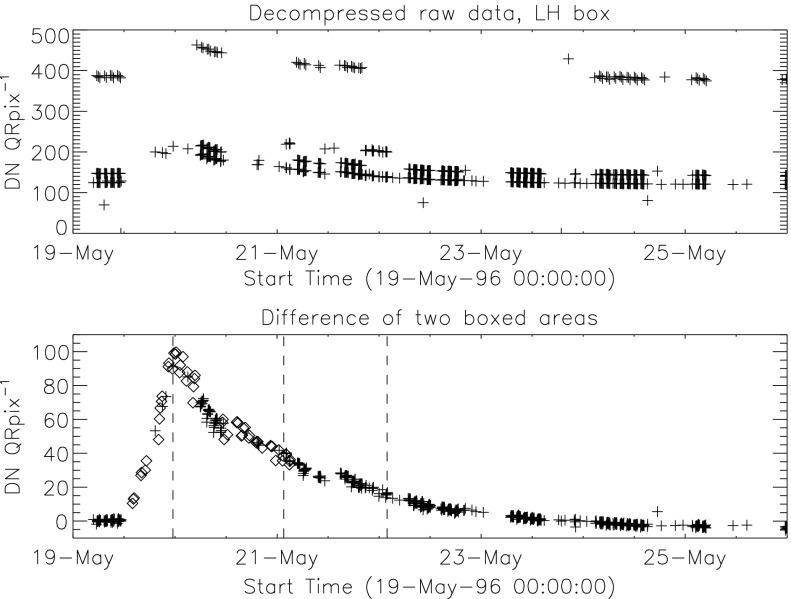
Rise and decay of the May 1996 glitch. The upper panel shows the light curves from QR dark images for the leftmost (glitch) rectangle of Figure 48 with exposures of 8 ms (lower curve) to 7.5 s (upper curve). In the lower panel the diamonds are glitch data from HR exposures shifted upwards by a factor of 2.4 to fill in the glitch-curve gap. Vertical dashed lines are times of table uploads from Kagoshima Space Center.

The constancy with exposure is demonstrated in Figure 49 where the top panel shows the average signal within the glitch for quarter resolution (QR) exposures ranging from 8 ms to 7.6 s, uncorrected for stray light or normal dark current. Note that the glitch enhancement is not proportional to exposure but is within a factor of 2.5 in DN for all exposures whereas the exposure times are different by a factor of almost 1000. The lower panel shows signals (from the ${<}\,1~\mbox{s}$ exposures) with the average signal in the adjacent box subtracted. The negative values reflect the fact that these data are not corrected for stray visible light. The box on the right in Figure 48 is in a slightly higher leak-intensity area than the box to the left.

The lower panel of Figure 49 reveals some interesting properties of glitch creation and decay. This particular glitch resulted from the upload of a PFI table on 18 May 1996, 04:02 UT that kept the shutter open most of the time. Throughout this period the GOES level was in the A range or below, and falling. Note that the glitch first appeared at about noon on 19 May, some 32 h after the table upload. The dark current signal gradually built up over the next 12 h until a standard table was uploaded at 23:30 UT, 19 May 1996, indicated by the leftmost vertical dashed marker on the plot. These data demonstrate that there is some threshold level of ionizing radiation damage beyond which a dark current glitch begins to appear.

**Figure 50:**
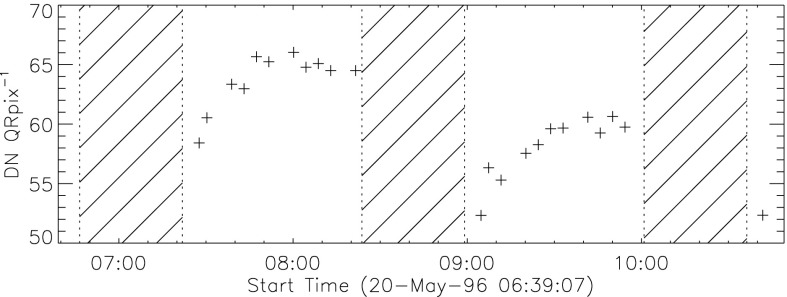
Signal in the glitch rectangle of Figure 48 minus the signal in the adjacent non-glitch rectangle showing the orbital recharging of the glitch following the morning interval. Quarter resolution, 668 ms exposures. Cross-hatched intervals are orbit night.

The decay of the glitch is interrupted a couple of times by some observing activity which pumped the damaged area back up a bit. As shown in Figure 50, after each orbit night the glitch is substantially reduced by the morning UV flood. During the day the X-ray exposure steadily increases the glitch intensity. This is in contrast to the normal dark signal amplitude (see Section 10.2), which is high immediately after the UV flood and decreases through orbit day.

Normal SXT operation precluded exposures so long as to create glitches. Their frequency in the data is unknown but is not large. The example shown in Figure 48 is the most intense observed. This study was undertaken to try to find a way to improve the dark correction of glitches. Given the time variability of glitching there seems to be no obvious way to automatically correct for glitches.

#### X-Ray Sensitivity

It was discovered during pre-launch tests that ionizing radiation damage to the CCD, if sufficiently severe, caused a decrease in charge transfer efficiency and enhanced dark current. However, as reported by Acton *et al.* (1991) the two halves of the virtual phase CCD pixel react differently to visible light and X-rays so that impact on visible light and X-ray images may be different. It is thus important to ascertain, in orbit, if ionizing radiation damage as observed in visible light images is reflected in X-ray sensitivity.

**Figure 51:**
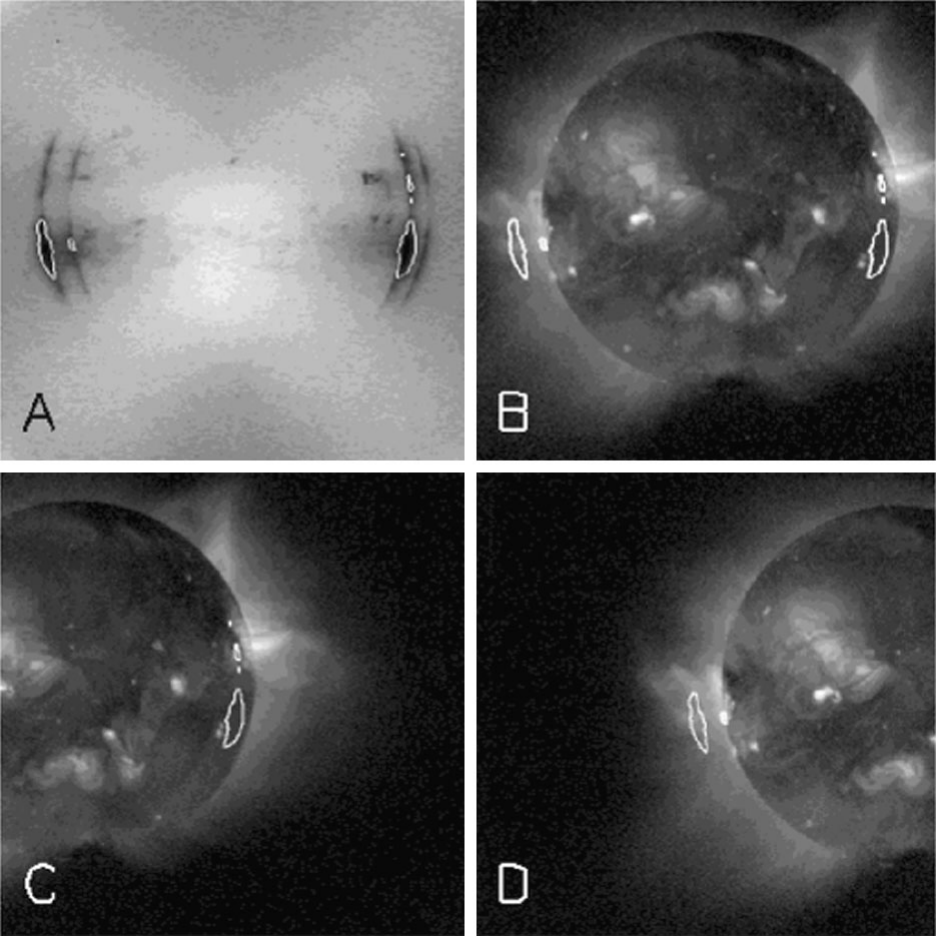
Sample areas for test of SXT X-ray quantum efficiency in optical burn areas. (A) diffuser image of 26 August 1992, 19:25 UT. Contours denote areas of strongest ionizing radiation damage (burns). (B) log-scaled X-ray image (Al.1 filter) obtained at 19:50 UT with burn contours. (C) X-ray image taken at 21:25 UT during a *Yohkoh* offpoint to the west. (D) X-ray image of 22:58 UT during offpoint to the east. Burn contours in the X-ray images show the image areas sampled for the signal level plots of Figure 52.

For the diffuser image of Figure 51 the burned areas have a signal decrease of about 20 % as noted in Section 7.4. If the X-ray sensitivity in the burned areas was similarly decreased the features would be immediately obvious in the X-rays images, which they are not.

**Figure 52:**
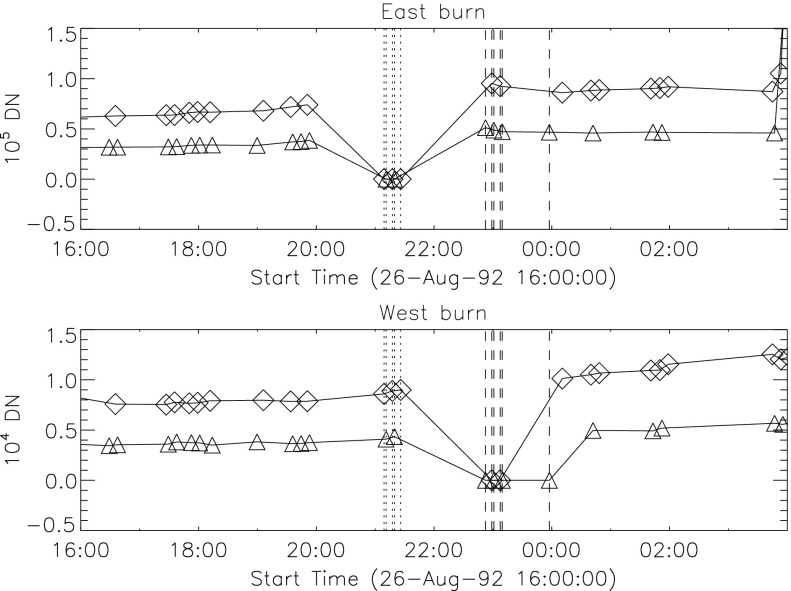
Time sequences of the X-ray intensity in the burn areas shown in Figure 51. Diamonds show signals from Al.1 and triangles from AlMg analysis filters. The upper panel plots data for the eastern (above limb) burn and the lower for the western (on disk) quiet corona burn. Dotted lines mark images acquired during the west offpoint and broken lines the east offpoint.

A more stringent test is to determine the intensity of the same portion of the solar image when it is on a burned area of the CCD *versus* when it lies on an unburned area. The periodic offpoints of *Yohkoh* provides the opportunity to do this as illustrated in Figures 51 and 52. Although Figure 52 shows minor changes in X-ray intensity in the east and west areas over the 12 h observing interval there are no discernible differences associated with the sample area being recorded by the burned or unburned area of the CCD.

We conclude that, at the level of ionization damage seen in this example and throughout the mission, the X-ray recording properties of the CCD were not impacted by the ionization damage. In the rare cases when the damage was so severe as to cause the glitches in dark current discussed in Section 9.2.2 we are not certain that X-ray recording was not temporarily impacted.

### CCD Damage from Energetic Particles

Ionization and lattice damage to CCDs from energetic particles is a subtle and complex study (Janesick, 2001) far beyond the scope of this paper. For our purposes it is sufficient to note that protons and electrons from the earth’s trapped radiation belts and, to a lesser degree, galactic cosmic rays caused noticeable changes in the virtual phase CCD employed on the SXT. The observed changes were the creation of so-called ‘hot pixels’ or ‘dark spikes’ (interchangeable terms used by different authors), *i.e.*, increased average dark current. Reference (Gburek and Sylwester, 2002) describes and illustrates the dark spike issue.

According to Janesick (2001) charge transfer efficiency (CTE) of the damaged pixel is also impacted by radiation damage. Modest decreases in CTE of a single pixel of the image readout chain will have very little effect on the data. SXT images and derived X-ray fluxes are not noticeably different at the end of the ten year mission than at the beginning.

**Figure 53:**
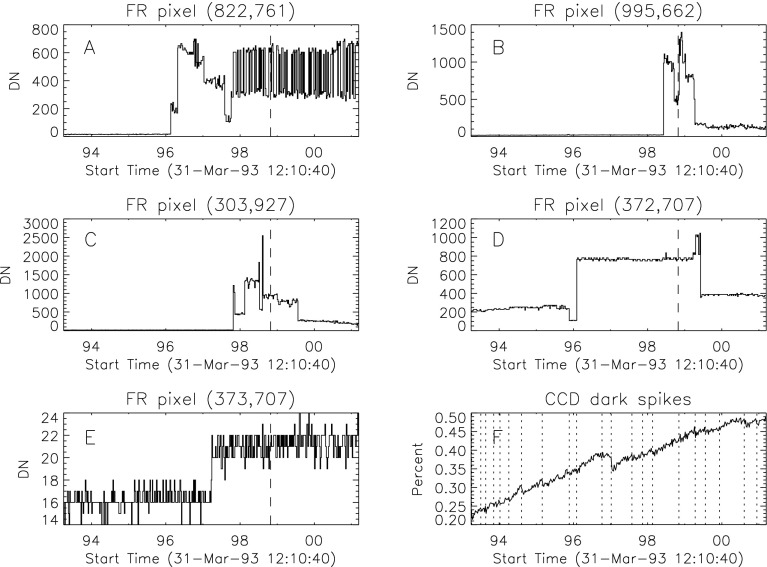
Dark signals in individual SXT CCD pixels of full-resolution (FR) dark frames with 30 s exposure. Panels A – D present the dark current signal in randomly chosen hot pixels. Panel E shows the dark signal in a normal pixel adjacent to the pixel of panel D. Broken vertical lines denote the time of the randomly chosen dark frame from which the dark spike pixels were selected. Panel F demonstrates the increase in fraction of hot pixels throughout the mission. The plots begin in 1993 because prior to that time 30 s FR dark frames were not acquired. The vertical dotted lines in panel F denote times of bakeout of the CCD.

Dark spikes are pixels for which the dark current is much larger than would be expected from dark current statistics. For this analysis I have arbitrarily defined a dark spike as any pixel whose dark current is more than five standard deviations larger than the average dark current. Figure 53 illustrates the mission-long dark signal of four randomly chosen hot pixels and one normal pixel adjacent to a dark spike. Note that the dark current of SXT hot pixels is not, in general, stable over long time spans. During SXT operations dark frames were acquired on a weekly basis. Thus, the data samples comprising Figure 53 are approximately weekly. To the extent that dark spikes persist for weeks or months they are removed from SXT images by dark-frame subtraction.

Figure 53(E) shows the dark current signal of a normal pixel immediately adjacent to a dark spike. Note the step in dark current from minor high energy particle damage. It is this kind of damage that caused the average dark current to continue to increase as the mission progressed. CCD bakeouts caused a modest decrease in dark current.

Panel F of Figure 53 illustrates the percentage of CCD pixels identified as hot pixels *versus* time. After a decade in space about 0.5 % of the pixels have become dark spikes. However, note that dark spikes switch on and off and change dark current level as time progresses. Also note that the long CCD bakeout of January 1997 caused a temporary decrease in the number of dark spikes.

### CCD Bakeouts

**Figure 54:**
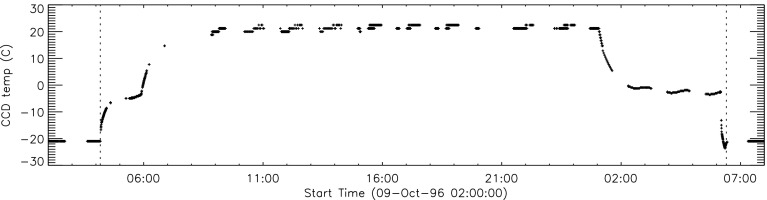
CCD bakeout of October 1996. The vertical dotted lines denote the commanded bakeout interval.

**Figure 55:**
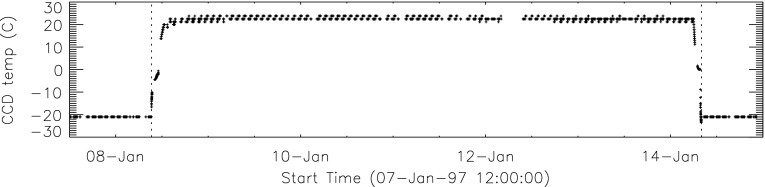
Extra-long CCD bakeout of January 1997. The vertical dotted lines denote the commanded bakeout interval.

Early in the *Yohkoh* mission it was inferred, from absorption features appearing in SXT full-resolution (FR) images, that contamination was collecting on the surface of the CCD. In January 1992 a series of warmings of the CCD to room temperature (+20 °C, termed ‘bakeouts’) was initiated to evaporate off the contaminating material. Careful examination of FR PFI images indicate that the contaminating material tended to disappear at a temperature of 0 °C although the extremely low ambient pressure would seem to rule out water ice as the contaminant. Bakeouts continued throughout the mission although late in the mission the evidence for contamination was never evident in the X-ray images. It took a very long time for the outgassing products to escape from the SXT and spacecraft. The intervals and duration of CCD bakeout, and the maximum CCD temperatures achieved are listed in http://solar.physics.montana.edu/ylegacy/yo_dates/warm_ccd.html. The time profile of typical bakeouts are illustrated in Figures 54 and 55.

The January 1997 CCD bakeout decreased dark current as illustrated in Figure 61(A). It is not clear that the shorter routine bakeouts had much effect on dark current. The dark current accumulation time is set by exposure time plus time added by image readout at a rate of $131\mbox{,}072~\mbox{pixels}\,\mbox{s}^{-1}$. On-chip pixel summation simply increases the dark current signal per pixel by the number of FR pixels summed, *i.e.*, four for HR dark frames and 16 for QR.

## SXT Dark Signal Removal

**Figure 56:**
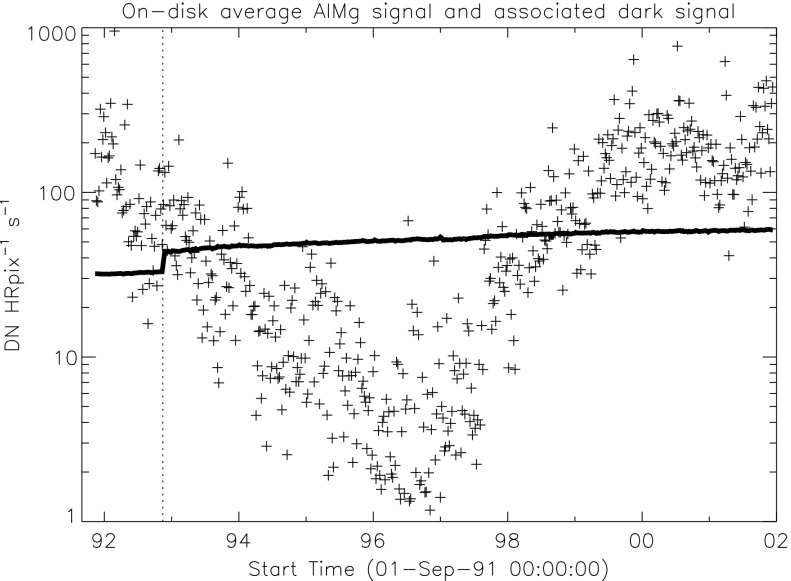
Comparison of mean AlMg on-disk X-ray intensity (crosses) and CCD dark signals (heavy line). The dotted vertical line indicates the time of the first entrance filter failure on 13 November 1992. These HR images were chosen at a cadence of one per week from the YLA level-2 data base.

**Figure 57:**
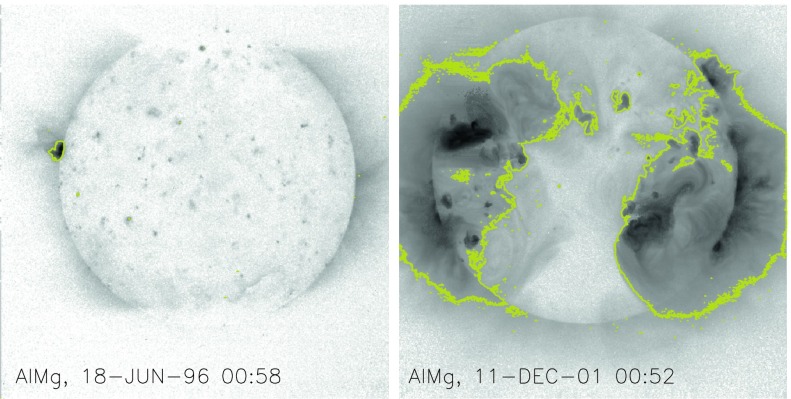
Log scaled SXT reverse color table images obtained at sunspot minimum (left) and near the peak of solar cycle 23 (right). The yellow contours illustrate the CCD dark signal levels for the two images.

**Figure 58:**
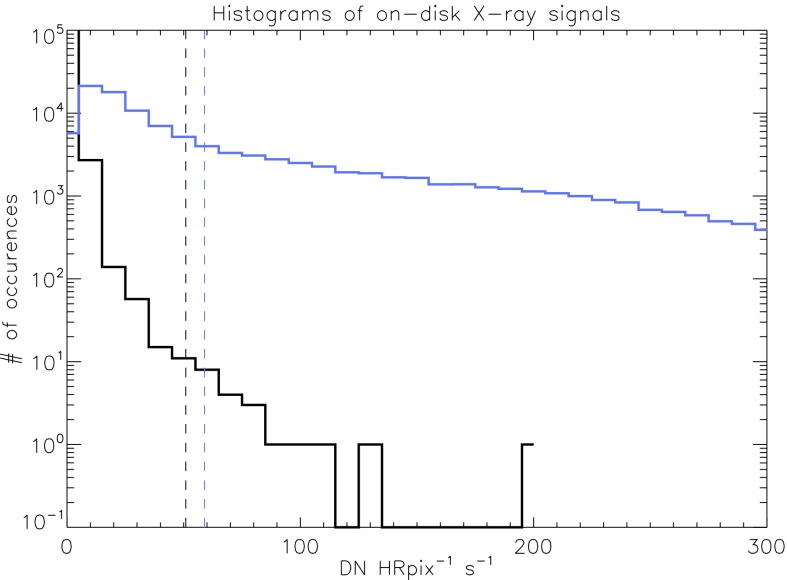
Histograms of the on-disk portion of the X-ray images shown in Figure 57. Most of the bright east limb feature of the solar minimum image lay above the limb so is not included in the black histogram. Even in the case of the solar maximum image (blue histogram) more than half of the pixels are less than the dark signal level. The dark signal levels for the two cases are indicated by the broken vertical lines.

Although the X-ray signal from flares and active regions are much larger than the dark signal from the SXT CCD camera the same is not true for the quiet portions of the X-ray image. For faint parts of the images the signal is of the order of, or even less than, the CCD dark-frame signal. Even in active times, coronal hole signals may be only of the order of $10~\mbox{DN}\,\mbox{HRpix}^{-1}\,\mbox{s}^{-1}$. Near sunspot minimum the average on-disk X-ray signal is well below the dark signal because of the long exposures required to record the quiet corona. These realities are illustrated in Figures 56, 57, and 58. Even in Figure 57 image obtained near the peak of sunspot cycle 23 much of the X-ray signal is below the CCD dark signal. Thus, it is important to do the best possible dark-frame subtraction from SXT images.

SXT dark images (called SDC for SXT dark current) are simply shutter-closed ‘exposures’ with filter wheels set for best stray-light rejection. After the entrance filters began to fail we always employed filter wheel A in the open position and filter wheel B at the AlMg position. SDCs were acquired once per week using a standardized observing table beginning about April 1993. Prior to that time acquisition of SDCs was less standardized but adequate. For special observing conditions or experiments such as during CCD bakeouts or coordinated observing campaigns SDCs were also acquired as part of the science observing table. The SDC database comprises those SDC FFIs which are 100 % complete and not taken in the South Atlantic Anomaly. Long and short dark exposures are gathered for FR (both halves of the CCD), HR, and QR resolutions. The program *dark_sub.pro* interpolates between the long and short exposures to create an SDC appropriate for each X-ray picture.

LaBonte (1994) carefully studied the properties and errors of SXT dark-frame correction. Unfortunately, some of his recommendations could not be implemented because of data rate limitations.

### SXT Dark Frame Properties

**Figure 59:**
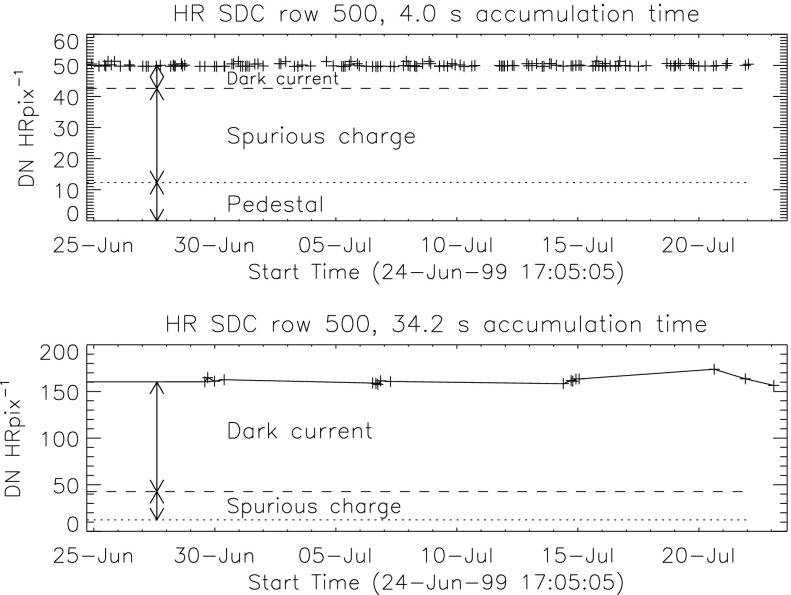
Examples of HR dark signal for very short (7.91 ms, $\mathrm{DPE}=2$) and very long (30.2 s, $\mathrm{DPE}=30$) ‘exposure’ times. The time to read out the image to row 500 takes 3.9 s, accounting for the longer accumulation times.

The SXT dark signal comprises three distinct components as discussed in general by Janesick (2001) and for the SXT by LaBonte (1994) and illustrated in Figure 59. The pedestal (called bias by LaBonte) is a fixed offset from zero of about 12.5 DN. As it is set in the CCD camera this is not expected to vary. The spurious charge (also called read noise) is created in the CCD readout process. For FR images it is not possible to separate the pedestal and spurious. In summation modes the spurious increases by factors of 4 and 16 for HR and QR, respectively. For a given resolution (pixel summation) the pedestal and spurious are, in principal, fixed and independent of exposure and show no orbital variation. For SXT the pedestal+spurious increased sharply following the first entrance filter failure and exhibited mild secular variation thereafter. Finally, the dark current signal reflects damage to the CCD from energetic particles, scales with accumulation (exposure plus readout) time, and increased approximately linearly throughout the mission.

**Figure 60:**
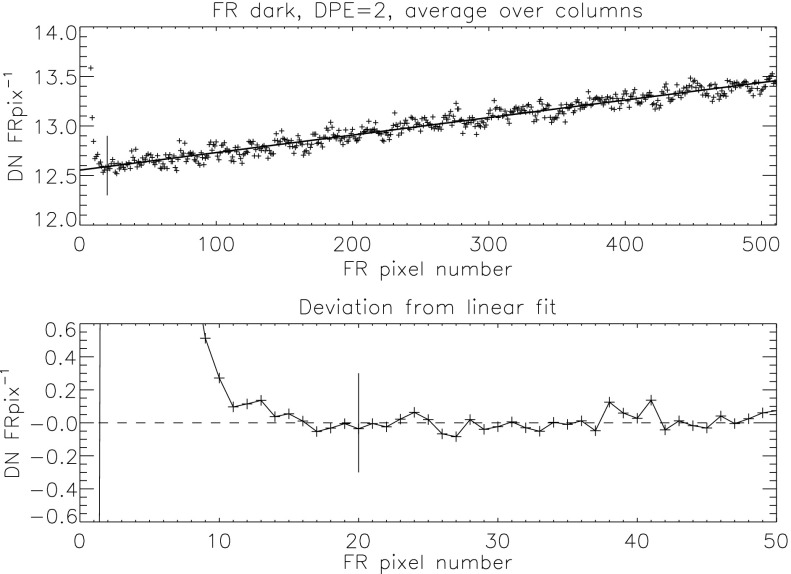
Upper: fit to dark current wedge from the average of three FR dark images obtained before entrance filter rupture (26 October 1992, 27 October 1992, and 11 November 1992). Lower: deviation of signals from fit for bottom 50 rows. The vertical bar at row number 20 indicate where the CCD pedestal+spurious has been measured.

Because of the time to read out each row the dark current part of the dark signal increases as a wedge from the bottom to the top of each CCD image. This is illustrated in Figure 60.

Our best estimate of the pedestal+spurious signal can be obtained by analysis of $\mathrm{DPE}=2$ dark images. For the bottom rows of the image there is negligible time to accumulate a dark current signal. However, the first $n$ rows must be avoided because of charge bleed back from the serial register. Figure 60 demonstrates that row 20 is free from bleed back for FR images. The intercept of the fit, 12.55 DN, is close to the expected pedestal. The slope of the fit ($0.001767~\mbox{DN}\,\mbox{FRpix}^{-1}\,\mbox{row}^{-1}$) gives the dark current rate ($0.226~\mbox{DN}\,\mbox{FRpix}^{-1}\,\mbox{s}^{-1}$). It takes 0.0078125 s to read out a single 1024 pixel row at $131\mbox{,}072~\mbox{pixels}\,\mbox{s}^{-1}$. By the end of the mission the FR dark current rate had increased to an average of $1.051~\mbox{DN}\,\mbox{FRpix}^{-1}\,\mbox{s}^{-1}$, a factor of 4.2. By this time approximately 1 % of the CCD pixels were so-called dark spikes with a dark current rate many times higher than expected statistically. See Section 9.3 for a discussion of SXT dark spikes.

For HR and QR dark frames row 15 has been chosen for pedestal+spurious measurement based upon similar analyses.

#### Quarter Resolution Case

**Figure 61:**
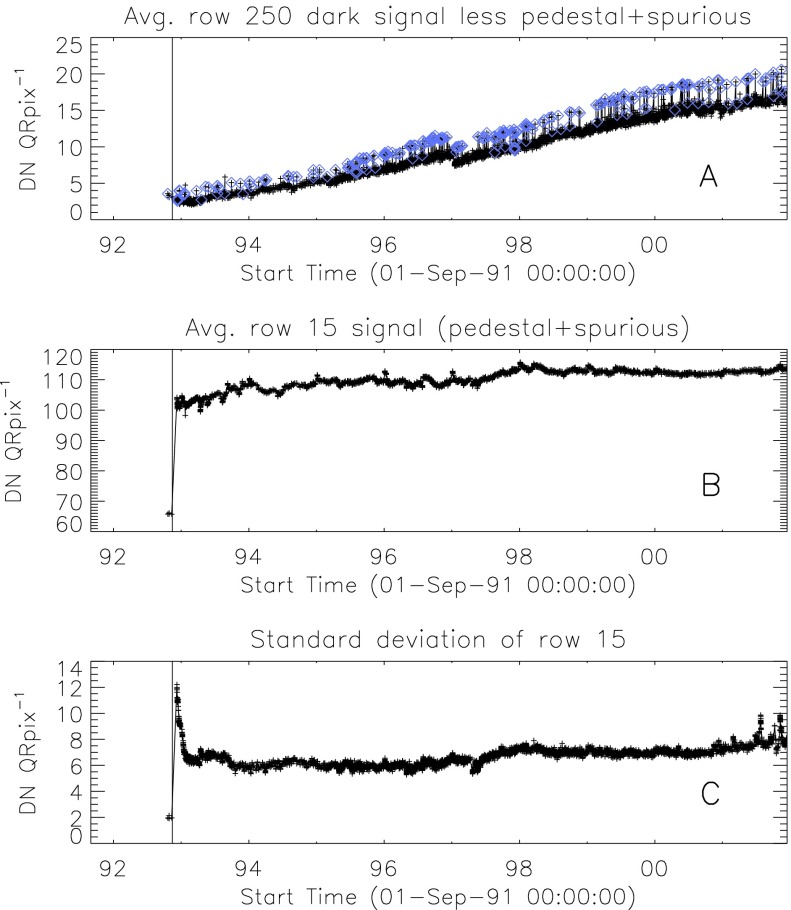
Signals in quarter resolution dark frames with 7.91 ms accumulation times. Vertical line marks the time of the 13 November 1992 entrance filter failure. The data marked with blue diamonds in panel A are all from dark frames acquired less than 3.5 minutes following the end of UV flood.

Quarter resolution (QR) images sum, during CCD readout, the $1024\times 1024$ CCD image into a $256\times256$ output image with a pixel resolution of about 9.8 arcsec. QR images were acquired when data rate limitations precluded the use of larger images. Sixteen FR pixels are clocked into a single QR pixel so the QR dark signal is approximately 16 times greater than for FR images. QR dark frames with 7.91 ms exposure were routinely acquired throughout the mission as dark signal monitors. Thus, they are particularly useful for secular analysis. For example, there are 7734 QR dark frames represented in Figure 61. The corresponding number for HR is 3106 and for FR it is 1674.

The large increase in dark signal demonstrated in Figure 61(B) is all due to an increase in spurious charge somehow stimulated by the more intense and spectrally different UV flood (Section 9.2.1). Figure 61(A) shows little if any change in dark current at the entrance filter-failure event.

**Figure 62:**
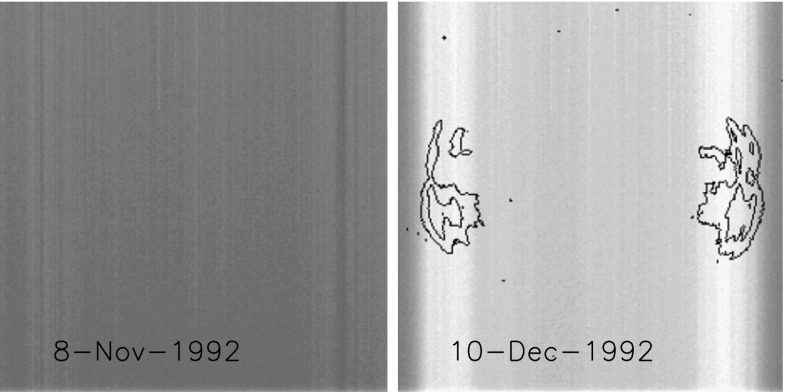
Change in QR 7.91 ms dark frame caused by the entrance filter failure of 13 November 1992. Dates of the two dark images are shown. The relative intensity scales of the two images have been adjusted to reveal the real change in dark signal. The contours in the right hand image are taken from Figure 26(B) and clearly demonstrate the spatial relation between areas damaged by ionizing (X-ray) radiation and increased spurious charge.

Our biggest surprise was the sharp spike in the standard deviation of row 15 signals evident in Figure 61(C). It took several months for the CCD to reach a new equilibrium. The standard deviation increase is not random read noise but is caused by a definite fixed pattern related to the CCD areas of greatest ionizing radiation damage (Section 7.4) as illustrated in Figure 62. It appears that following the entrance filter failure and change in the spectrum and intensity of the UV flood increased spurious charge was produced as CCD rows were clocked through the radiation damaged areas. This spurious remained in the pixels below the damaged areas following readout or CCD flush so the enhanced dark signals appears uniformly from bottom to top of the dark image. That is, every row of the CCD image has passed through the damaged area either before or after a given exposure. This increase in spurious associated with damage by ionizing radiation and the effects of the UV flood may well may be related to the virtual phase architecture of the SXT CCD (Janesick, 2001).

**Figure 63:**
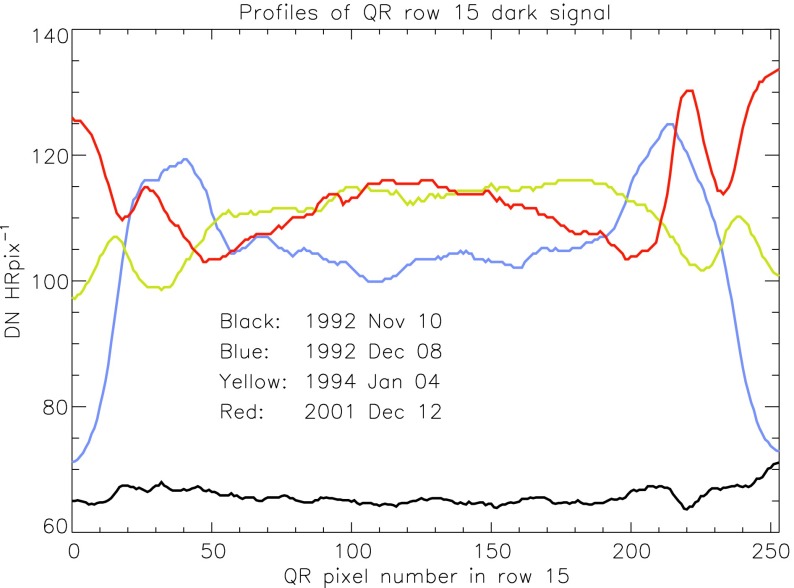
Evolution of CCD spurious charge through the mission. All accumulation times were 7.91 ($\mathrm{DPE}=2$). The curves have been smoothed with an eight pixel boxcar averaging.

The evolution of spurious charge across QR row 15 throughout the *Yohkoh* mission is illustrated in Figure 63. Note the spurious increases at the edge columns of the CCD by mission end. As shown in Figure 45 the edges of the CCD receive less UV flood than the center. The HR and FR dark frames show a similar pattern of evolution of spurious charge.

Thankfully, this complex and time varying CCD dark signal is well controlled in the X-ray images by appropriate dark-frame collection and subtraction procedures.

#### Half Resolution Case

SXT HR images bin $2\times2$ FR pixels into a single HR pixel producing a $512\times512$ image with a $4.9~\mbox{arcsec}\,\mbox{pixel}$ resolution. This is the preferred mode for SXT full-disk images and was used throughout the mission with Al.1 and AlMg analysis filters for solar monitoring. All dark-frame conclusions described in Section 10.1.1 are also true for HR dark frames.

**Figure 64:**
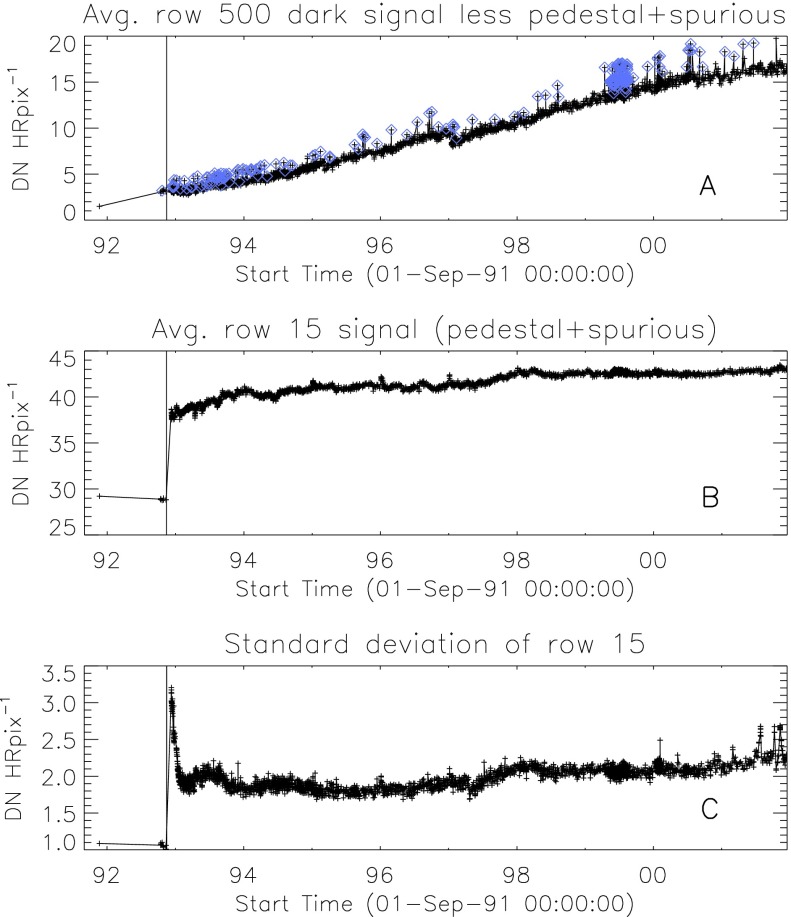
Signals in half resolution dark frames with 7.91 ms accumulation times. Labels and markers are the same as in Figure 61.

Note the clump of blue, high, signals in Figure 64(A). These are all from dark frames acquired within a few min of the end of the UV flood. This block of dark frames in mid-1999 resulted from a command table error that provided a nice data set for preparation of Figure 66.

#### Full Resolution Case

**Figure 65:**
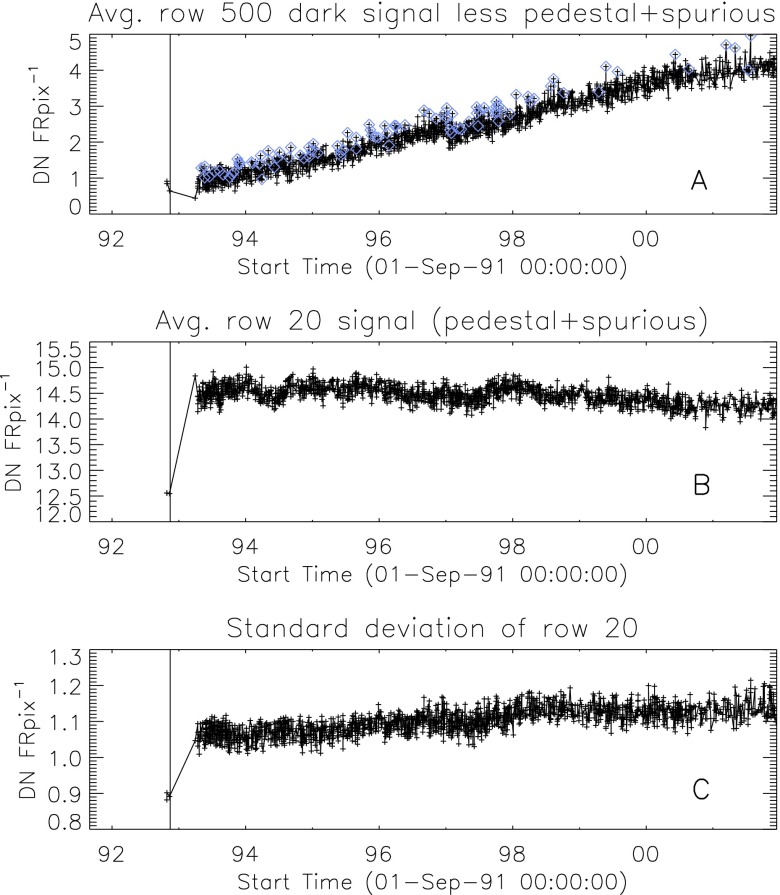
Signals in full-resolution dark frames with 7.91 ms accumulation times. Labels and markers are the same as in Figure 61.

Full-resolution (FR) FFIs were not routinely taken because of telemetry limitations. The *Yohkoh* data processor buffers could only handle up to a $1024\times512$ image so FR FFIs had to be taken in two shots. Likewise the FR dark frames required two separate exposures to cover the entire CCD. FR $\mathrm{DPE}=2$ dark frames were not routinely acquired before the end of March 1993. Fortunately, three such images of the bottom half of the CCD were taken shortly before the 13 November 1992 entrance filter failure and are included in Figure 65. This figure is based entirely on bottom-half dark frames. All conclusions presented in Section 10.1.1 apply equally to the FR dark frames.

### CCD Dark Current Orbit Correction

**Figure 66:**
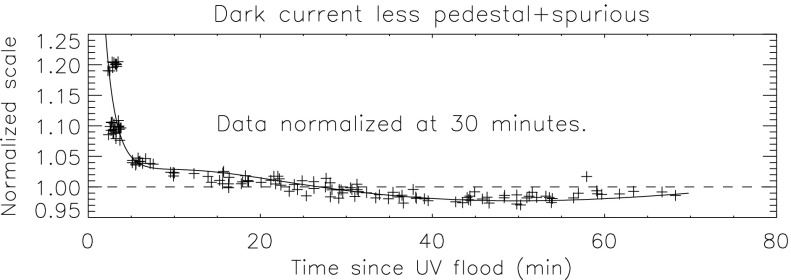
Variation of CCD dark current with time since UV flood. HR, $\mathrm{DPE}=2$, dark frames obtained in 1999. Solid line is a fifth order polynomial derived by Acton (1994) from other, specially acquired and more extensive, observations.

There is a drift in CCD dark current throughout each daylight pass of the SXT orbit, engendered by the UV flood at each orbit sunrise (Acton, 1996). This effect is illustrated in Figure 66 which gives the dark current *versus* time since UV flood. The fitted curve is an orbit-correction algorithm derived in 1994 (Acton, 1994) from special SXT observations. Knowing the time since UV flood of the dark frame and the corresponding time of the X-ray image it is possible to use this algorithm to adjust the dark current part of the dark signal to what it would have been had the dark-frame and X-ray image been taken at the same time. The corrections are generally small compared to other sources of error. The algorithm is adequate beyond 4 or 5 min past the end of the UV flood. For routine analysis, dark frames acquired earlier than 4.5 min after orbital sunrise are not used. SXT analysis software, *e.g.*, *sxt_prep.pro* include orbit-dependent dark signal correction by default.

Very unfortunately, beginning in 1998, routine SXT data reduction mistakenly applied this orbit correction to the ENTIRE dark signal rather than just to the dark current part. In general the quantitative impact of this error is difficult to assess. It will be small for analysis of active regions and flares as in these cases the dark signal is much smaller than the X-ray signal. As illustrated in Figure 59, for very long exposures, the dark current dominates the dark signal so the error will be less – except for the fact that the X-ray signal in such cases will be smaller than the dark signal so the percentage error in the derived X-ray intensity could be substantial. For any given case the error will depend on the relative orbital timing of the dark and X-ray images.

We judge, but have not demonstrated in general, that few investigations employing SXT data will be significantly affected by this unfortunate data processing error. As of early 2016 the level-1 and level-2 SXT data in the YLA will have been reprocessed with repaired orbit-correction software.

## Summary

By any standard, *Yohkoh* was an eminently successful scientific mission as evidenced by the more than 1500 refereed publications traced to it, reported in http://www.lmsal.com/~aschwand/publications/yohkoh.html. X-ray observations of the active Sun and quiet corona, taken by the *Yohkoh* SXT from October 1991 until December 2001, will be scientifically valuable on into the future. They are unique because of the epoch of the observations, the quality of the images, and the spectral response of the telescope. The S-054 X-ray photographs from Skylab (14 May 1973 to 8 February 1974) (Batchelor, 1995) dramatically demonstrated the worth of X-ray movies for studies of solar activity. The SXT carried on that tradition with greatly improved resolution, cadence, quantitative accuracy, usability, and accessibility. The purpose of this paper has been to document many important aspects of SXT data and their preparation for serious future users. Issues such as outgassing problems, filter failures, and CCD performance have been treated in sufficient detail to, hopefully, be of benefit to future space experimenters.

## Appendix: ATT File Format

The ATT data base comprises weekly files named, *e.g.*, att99_33.18. Here, 99 refers to the year 1999, 33 designates week 33 of that year, and 18 identifies the version number. These weekly files are not strictly calendar weeks because *Yohkoh* daylight passes are not permitted to be broken by a week boundary. Thus, att99_33.18 could begin up to 90 min after Saturday midnight and include data up to an hour into week 34.

There is one record in the ATT file for each and every SXT image. The records are in the form of IDL structures. For example,

** Structure ATT_SUMMARY_REC, 9 tags, length=24, data length=24:

   TIME            LONG          78564340

   DAY             INT           4639

   DP_MODE         BYTE       141

   DP_RATE         BYTE       128

   PNT             LONG      Array[3]

   STATUS1         BYTE         2

   STATUS2         BYTE        12

   ADS             BYTE         1

   SPARE           BYTE      Array[1]

Here the tag PNT contains the attitude information, *i.e.*:

-   PNT(0)/100. is the $X$ coordinate of Sun center in CCD pixels,
-   PNT(1)/100. is the $Y$ coordinate of Sun center in CCD pixels, and
-   PNT(2)/36000. is the S/C roll angle in degrees (positive clockwise).

TIME and DAY are time codes and the other tags provide housekeeping information.

## Footnotes

1 http://solar.physics.montana.edu/ylegacy/documents/SXT_EICA.pdf.
